# Rare-Earth Molecular Cluster Aggregates with Sandglass-like Core Topology as Surrogates for Minor Actinides in Immobilization within Alkaline-Earth Manganites

**DOI:** 10.1021/acs.inorgchem.5c02206

**Published:** 2025-07-25

**Authors:** Rafał Petrus, Adrian Kowaliński, Tadeusz Lis, Miłosz Siczek, Piotr Sobota

**Affiliations:** † Faculty of Chemistry, Wrocław University of Science and Technology, 23 Smoluchowskiego, 50-370 Wrocław, Poland; ‡ Faculty of Chemistry, 49572 University of Wrocław, 14 F. Joliot-Curie, 50-383 Wrocław, Poland

## Abstract

In this study, a series of rare-earth molecular cluster aggregates with the formula [M_9_^RE^(μ_4_-OH)_2_(μ_3_-OH)_8_(sal-R)_16_]­X (M^RE^(III) = Y (**1**), Eu (**2**), Dy (**3**), Tm (**4**), Yb (**5**), Lu (**6**); X^–^ = Cl^–^ (**1**–**2**, **4**–**6**), or DyCl_4_^–^ (**3**); Hsal-R = alkyl salicylate with R = Me (**2**, **4**–**6**), Et (**3**), or Me and Et (**1**)) were synthesized via the direct reaction of rare-earth chlorides (M^RE^Cl_3_) with in situ generated lithium or zinc methyl salicylate salts in a ROH/tetrahydrofuran (THF) solution (ROH = MeOH, EtOH) under controlled moisture conditions. Complexes **1**–**6** were investigated as surrogates for minor actinides, with the aim of immobilizing these species in alkaline-earth manganites. The manganites were obtained by thermal decomposition of mixtures comprising either [Ca­(sal-Et)_2_]*_n_* (**7**) or [Ba­(sal-Et)_2_(THF)]*_n_* (**8**) with [Mn_2_(μ-OMe)_2_(sal-Me)_4_] (**9**) at 850 or 1100 °C. Thermolysis of compounds **7** and **9** in the presence of 0.22 or 1.11 mol % **1**–**6** at 1100 °C yielded M^RE^-doped CaMnO_3_ as the major phase, with variable amounts of CaMn_2_O_4_. Under analogous conditions, decomposition of **8** and **9** produced BaMnO_3_:M^RE^(III); transmission electron microscopy (TEM) also identified nanorod-like crystallites of M^RE^MnO_3_. Alkaline-earth manganites, prepared via the aryloxide route, effectively incorporate M^RE^(III) ions at the Ca­(II)/Ba­(II) sites.

## Graphical abstract

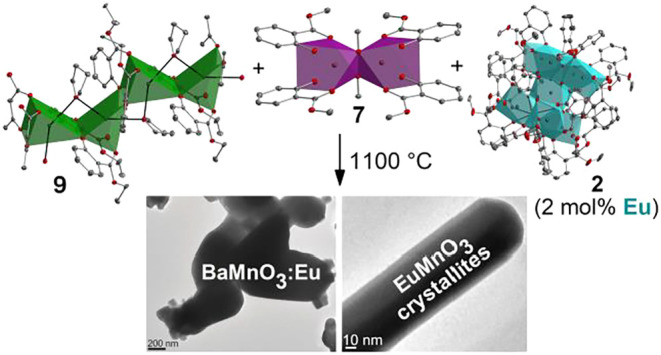

## Introduction

Over the past few decades, lanthanide molecular cluster aggregates (MCAs), composed of multinuclear metal cores encapsulated by organic ligands, have attracted considerable attention due to their intriguing solid-state structures and diverse applications in areas such as single-molecule magnetism,^1,2^ magnetic refrigeration,^3,4^ luminescent sensing/probing,^5–7^ and catalysis.^8–10^ More recently, MCAs have emerged as a new generation of highly efficient optical materials that combine the advantageous properties of both lanthanide nanoparticles (NPs) and molecular complexes. Owing to the chemical similarities among Ln­(III) ions, MCAs allow for facile compositional tuning and energy transfer processes analogous to those observed in NPs. Unlike nanoparticles, the well-defined molecular architecture of lanthanide clusters permits precise control over the local symmetry of Ln­(III) ions, resulting in enhanced homogeneity of optical properties.

Additionally, the bonding of organic ligands to the Ln­(III) centers promotes efficient ligand-mediated intramolecular energy transfer (antenna effect) without requiring surface modification, which is typically necessary in NPs.^11^ Consequently, heterometallic MCAs offer a platform for fine-tuning energy transfer pathways and emission properties, making them promising candidates for applications in luminescent thermometry, anticounterfeiting technologies, and molecular upconversion systems.

A common synthetic strategy for forming high-nuclearity lanthanide clusters involves the controlled hydrolysis of lanthanide salts LnX_3_ (X^–^ = NO_3_^–^, ClO_4_^–^) in the presence of auxiliary ligands. An alternative approach involves the substitution of alkoxy groups in [Ln_5_(O)­(OiPr)_13_] with ligands containing NH_2_, OH, or COOH functional groups. In these systems, hydrophilic groups (O^2–^, HO^–^) act as bridges between metal centers to form the central core, while hydrophobic terminal organic ligands stabilize the structure by preventing further aggregation.^12^ The resulting cluster architecture is highly sensitive to the nature of the Ln­(III) ion, the choice of organic ligand, solvent polarity, base strength, and the pH of the reaction medium.^13,14^ In such syntheses, anions like Cl^–^, NO_3_^–^, ClO_4_^–^, and CO_3_^2–^ often play a crucial templating role in guiding the formation of specific cluster topologies.^15,16^ A wide variety of lanthanide complexes have been synthesized, with nuclearities ranging from {Ln_4_},^17–19^ {Ln_5_},^20–23^ {Ln_6_},^24,25^ {Ln_7_},^26–28^ {Ln_8_},^29,30^ {Ln_9_},^31,32^ {Ln_11_},^33^ {Ln_12_},^34–36^ {Ln_14_},^37–39^ {Ln_15_},^40^ to {Ln_16_}.^41^ However, the synthesis of higher-nuclearity clusters remains challenging, and such species are relatively rare. Notable examples of high-nuclearity lanthanide clusters include [Ln_104_(μ_4_-O)_30_(μ_3_-OH)_168_(OAc)_56_(ClO_4_)_6_(H_2_O)_112_]­(ClO_4_)_22_ (Ln­(III) = Nd, Gd),^42^ [Er_60_(μ_6_-CO_3_)_8_(μ_3_-OH)_96_(μ-O)_2_(l-thr)_34_(H_2_O)_18_]­Br_12_(ClO_4_)_18_ (l-thr = l-threoninato),^43^ K_2_[Ho_48_(μ_5_-O)_2_(μ_4_-OH)_4_(μ_3_-OH)_84_(IN)_46_(OAc)_4_(H_2_O)_14_(CO_3_)­Br_2_] (IN^–^ = isonicotinato),^44^ {[Er_48_(μ_4_-OH)_6_(μ_3_-OH)_84_(N_3_)­(NA)_44_(H_2_O)_24_]­(NO_3_)­(Cl)_8_}*_n_* (NA^–^ = nicotinato),^45^ [Gd_48_(μ_4_-O)_6_(μ_3_-OH)_84_(CAA)_36_(NO_3_)_6_(H_2_O)_24_(EtOH)_12_(NO_3_)­Cl_2_]­Cl_3_, [Gd_38_(μ_8_-ClO_4_)_6_(μ_3_-OH)_42_(μ-O)­(CAA)_37_(H_2_O)_36_(EtOH)_6_]­(ClO_4_)_10_(OH)_17_ (CAA^–^ = chloroacetato),^46^ [Ln_33_(μ_5_-OH)_4_(μ_5_-CO_3_)­(μ_4_-CO_3_)_3_(μ_3_-OH)_36_(EDTA)_12_(OAc)_2_(H_2_O)_38_] (Ln­(III) = La, Sm),^47^ and [Ln_27_(μ_5_-CO_3_)_8_(μ_3_-OH)_32_(OAc)_20_(ClO_4_)­(H_2_O)_40_]­(ClO_4_)_12_ (Ln­(III) = Gd, Dy).^48^ Our focus centers on {Ln_9_} clusters due to their unique structural properties and promising potential in catalysis and materials chemistry. Nonanuclear Ln­(III) hydroxo clusters typically consist of two vertex-sharing square pyramidal units. These structures are stabilized by two μ_4_-OH, eight μ_3_-OH bridges, and 16 organic or inorganic ligands, yielding a sandglass-like central topology. A wide array of ligands has been employed in the construction of such clusters, including β-diketonates,^49,50^ alkoxides,^51^ aminoalkoxides,^52^ aryloxides,^53^ calixarenes,^54^ hydroxy benzamides,^55^ and Schiff bases.^56,57^ Nonanuclear motifs of this kind have been reported for coordination cations, anions, and neutral complexes. Examples include [Ln_9_(μ_4_-OH)_2_(μ_3_-OH)_8_(acac)_16_]­[HM_2_(CO)_10_] (M­(0/I) = Mo, Cr and Ln­(III) = Sm, Eu, Gd, Dy, Yb),^58,59^ (Me_3_NH)­[Eu_9_(μ_5_-O)_2_(μ_3_-OH)_8_(mpp)_16_] (mpp^–^ = 4-methyl-2-propanoylphenolato),^60^ and [Ln_9_(μ_4_-O)­(μ_4_-OH)­(μ_3_-OH)_8_(acac)_16_] (Ln­(III) = Eu, Tb; acac^–^ = acetyloacetonato).^61^ Among sandglass-shaped {Ln_9_} clusters, salicylate derivatives have proven to be particularly useful. A series of isostructural terbium­(III) complexes [Tb_9_(μ_4_-OH)_2_(μ_3_-OH)_8_(sal-R)_16_]­(NO_3_) ({Tb_9_(sal-R)}, where sal-R = alkyl salicylate; R = Me, Et, Pr, or Bu) show significant optical Faraday effects, relevant to fiber-optic telecommunications.^62^ The compound {Tb_9_(sal-Hex)} displays photosensitized Tb­(III) luminescence via ligand-to-metal energy transfer, with a quantum yield of 90%.^63^ Mixed-metal clusters such as {Tb_*x*_Gd_9–*x*_(sal-Bu)} (*x* = 0–9) reveal reduced back energy transfer between Tb­(III) ions, enhancing luminescence efficiency.^64^ Similarly, {Yb_*x*_Ln_9–*x*_(sal-Bu)} (Ln­(III) = Gd, Lu; *x* = 0–9) serve as the model system to evaluate spin–orbit coupling effects on energy transfer.^65^ Lanthanide clusters also serve in luminescence-based sensing applications for environmental and security purposes. For instance, (H_4_N)­[Tb_9_(μ_4_-O)_2_(μ_3_-OH)_8_(acac)_16_] detects nitroaromatic explosives,^66^ while (H_3_O)­[Tb_9_(μ_5_-O)_2_(μ_3_-OH)_8_(L)_8_(DMF)_8_] selectively senses Fe­(III), CrO_4_^2–^, and Cr_2_O_7_^2–^.^67^ [Ho_9_(μ_4_-OH)_2_(μ_3_-OH)_8_(L)_8_(NO_3_)_8_] (L^–^ = 2-(((pyridin-2-yl)­methylidene)­amino)­ethanolato) displays photoresponsive Y­(III) recognition.^68^ Yttrium complex [Y_9_(μ_4_-O)­(μ_4_-OH)­(μ_3_-OH)_8_(acac)_16_] serves as an efficient host matrix for doping with optically active Ln­(III) ions to form {Y_9–*x*_Ln_*x*_} clusters (*x* = 0.23 Pr, 1.95 Tb, 1.98 Dy, 1.66 Yb, 0.40–4.35 Eu), revealing a clear relationship between luminescence behavior and dopant distribution.^69^

Recently, lanthanide-based MCAs have emerged as promising building blocks for constructing multidimensional frameworks with novel physical properties, although this remains an innovative and relatively unexplored synthetic strategy.^12,70^ Lanthanide complexes have also been employed as single-source molecular precursors for the synthesis of functional inorganic materials;^71–73^ however, examples of such applications remain limited. One notable case involves a series of isostructural compounds, [M_4_^RE^(μ_3_-OH)_2_(accp)_10_] (M^RE^(III) = Y, Gd; accp^–^ = 2-acetylcyclopentanoate), which were used as spin-coating precursors to deposit thin M_2_^RE^O_3_ layers.^74^

In materials chemistry, studying radioactive materials presents significant challenges; therefore, nonradioactive lanthanide elements are often used as surrogates for minor actinides due to their similar chemical properties, outer electron configurations, valence states, and ionic radii.^75^ For example, Ce­(III) is commonly used to model Pu­(III), while La­(III), Nd­(III), and Eu­(III) are employed as surrogates for Ac­(III), Cm­(III), and Am­(III), respectively.^76^ The reprocessing of spent nuclear fuel produces high-level radioactive waste (HLW), consisting primarily of fission products (e.g., ^135,137^Cs­(I), ^87^Rb­(I), ^90^Sr­(II), ^93^Zr­(IV), ^99^Tc­(IV), ^129^I, and lanthanides) and transuranic elements such as ^239^Pu­(IV) and minor actinides (e.g., ^237^Np­(IV), ^241,243^Am­(III), and ^243,244,245^Cm­(III)), all of which exhibit long-lived radioactivity. Immobilizing these radioactive ions in highly durable and stable solid matrices is a key strategy to prevent nuclear waste migration and environmental contamination. Since the 1970s, heterometallic calcium- or barium-based oxide ceramic systems such as synthetic rock (Synroc) materials, including zirconolite (CaZrTi_2_O_7_), hollandite (Ba_1.2_(Al,Ti)_8_O_16_), perovskite (CaTiO_3_), and rutile (TiO_2_) have been developed for HLW immobilization.^77^ These alkaline-earth minerals can incorporate up to 20% HLW, by trapping and solidifying radionuclides into the periodic structures of the appropriate crystalline phases. However, due to their incongruent melting behavior, Synroc ceramics cannot be fabricated by using simple melting and casting techniques. Instead, the alkoxide route is employed to enhance homogeneity between the HLW and matrix components prior to thermal treatment. The resulting slurries are typically dried, calcined under reducing conditions, and hot-pressed in graphite dies. Both minor actinides and lanthanides can be effectively incorporated into the alkaline-earth sites of Synroc phases without inducing significant structural changes.^78^

In this work, rare-earth cluster aggregates with the formula [M_9_^RE^(μ_4_-OH)_2_(μ_3_-OH)_8_(sal-R)_16_]­X (M^RE^(III) = Y (**1**), Eu (**2**), Dy (**3**), Tm (**4**), Yb (**5**), Lu (**6**); X^–^ = Cl^–^ (**1**–**2**, **4**–**6**), or DyCl_4_^–^ (**3**); Hsal-R = alkyl salicylate with R = Me (**2**, **4**–**6**), Et (**3**), or a mixture of Me and Et (**1**)) were synthesized via direct reaction of trivalent rare-earth chlorides (M^RE^Cl_3_, where M^RE^(III) = Y, Eu, Dy, Tm, Yb, Lu) with in situ generated [Li_6_(sal-Me)_6_][Bibr ref79] or [Zn_4_(sal-Me)_8_][Bibr ref80] in a ROH/tetrahydrofuran (THF) solution (ROH = MeOH, EtOH) in the presence of trace amounts of water. The resulting clusters **1**–**6** were investigated as M^RE^(III) precursors for simulating minor actinides in high-level nuclear waste, subsequently immobilized in alkaline-earth manganites formed by thermal decomposition of mixtures of [Ca­(sal-Et)_2_]*_n_* (**7**) or [Ba­(sal-Et)_2_(THF)]*_n_* (**8**) with [Mn_2_(μ-OMe)_2_(sal-Me)_4_] (**9**) at 850 or 1100 °C.

## Results and Discussion

### Syntheses and Structural Study of Rare-Earth Molecular Cluster Aggregates

The reaction of 2 equiv of M^RE^Cl_3_ (M^RE^(III) = Y, Eu, Dy, Tm, Yb or Lu) with 1 equiv of [Li_6_(sal-Me)_6_] or 0.75 equiv of [Zn_4_(sal-Me)_8_] in a MeOH­(EtOH)/THF solution afforded crystalline oxo-aryloxo clusters of the formula [M_9_^RE^(μ_4_-OH)_2_(μ_3_-OH)_8_(sal-R)_16_]­X (M^RE^(III) = Y (**1**, 46%), Eu (**2**, 57%), Dy (**3**, 64%), Tm (**4**, 58%), Yb (**5**, 75%), Lu (**6**, 43%); X^–^ = Cl^–^ (**1**–**2**, **4**–**6**), or DyCl_4_^–^ (**3**); Hsal-R = alkyl salicylate with R = Me (**2**, **4**–**6**), Et (**3**), or a mixture of Me and Et (**1**)), and LiCl or ZnCl_2_, respectively (Scheme 1). These compounds were isolated as crystalline materials with a low solubility in conventional organic solvents. Single-crystal X-ray diffraction (XRD) analysis revealed that complexes **1**–**6** are ionic species composed of a nonanuclear [M_9_^RE^(μ_4_-OH)_2_(μ_3_-OH)_8_(sal-R)_16_]^+^ cation and a Cl^–^ (**1**–**2** and **4**–**6**) or DyCl_4_^–^ (**3**) counterion (Figures 1 and S1–S5).

**Figure 1:**
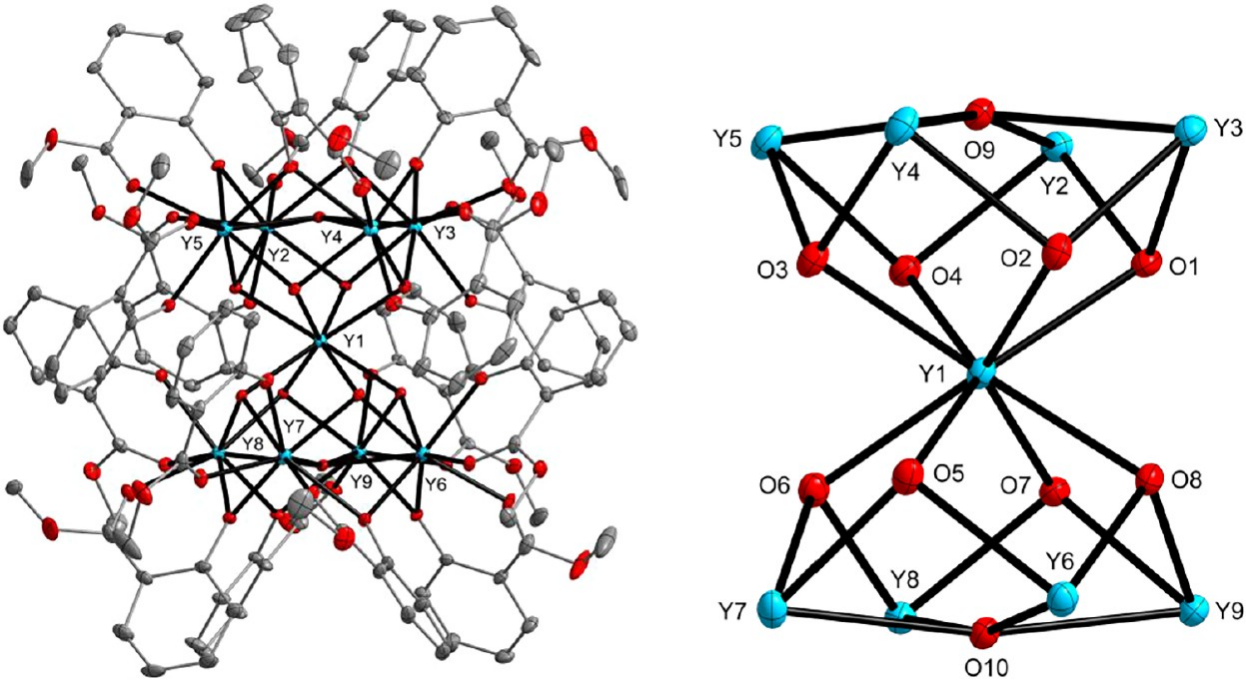
Molecular structure of [Y_9_(μ_4_-OH)_2_(μ_3_-OH)_8_(sal-R)_16_]­Cl (**1**) (for R = Me (0.70), Et (0.30)). Displacement ellipsoids are drawn at the 10% (left) and 20% (right) probability levels. Hydrogen atoms and disordered counterparts of aromatic ligands are omitted for clarity.

**Scheme 1:**
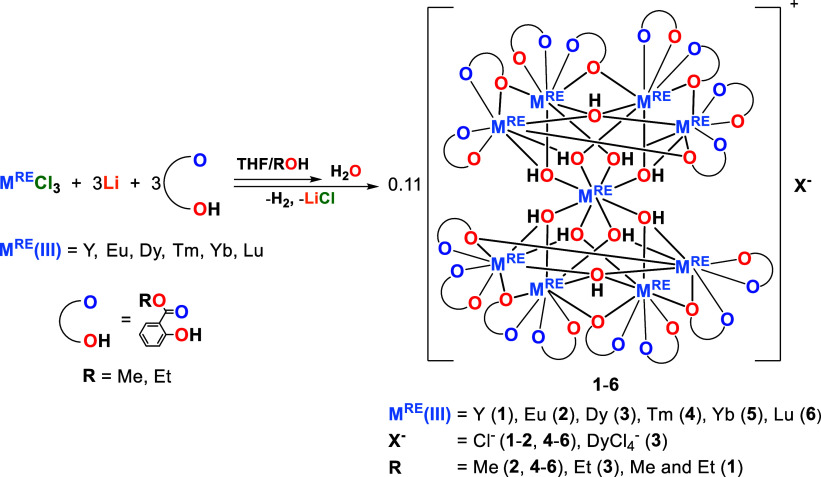
Synthesis of **1**–**6**

The use of EtOH alongside MeOH during synthesis improved solubility and crystallization efficiency but also led to partial or complete transesterification of sal-Me ligands in compounds **1** and **3**.

In **1**–**6**, the nine M^RE^(III) ions are bridged by two μ_4_-OH and eight μ_3_-OH groups, forming a central sandglass-shaped motif, {M_9_^RE^(μ_4_-OH)_2_(μ_3_-OH)_8_}^17+^. This core is encapsulated by 16 chelating sal-R ligands that stabilize the structure and inhibit further aggregation. Structurally, the M^RE^(III) centers form two {M_5_^RE^} square pyramidal units that share a common apex at the M^RE^1 center. These square pyramids are rotated approximately 45° relative to each other. Each of the eight M^RE^(III) ions in the square planes is connected to the neighboring metal ions through one μ_4_-OH, two μ_3_-OH groups, one bridging chelating sal-R ligand, and the aryloxo oxygen atom of another sal-R ligand. The coordination environment of these peripheral M^RE^(III) centers is completed by a terminal sal-R ligand, resulting in distorted biaugmented trigonal prismatic geometries. In contrast, the central M^RE^1 ion adopts a square antiprismatic coordination geometry, surrounded by eight μ_3_-OH oxygen atoms (Table S2).

In the coordination chemistry of M^RE^(III) complexes, a comparable central core topology has been observed in only 65 crystal structures to date, including 9 Y­(III),^69,81^ 1 Pr­(III),^54^ 3 Sm­(III),^58,59^ 11 Eu­(III),^82,83^ 10 Gd­(III),^51,52^ 11 Tb­(III),^63,84,85^ 10 Dy­(III),^49,86^ 3 Ho­(III),^67,68^ 4 Er­(III),^60,87^ and 2 Yb­(III) clusters.^6,58^ Notably, compounds **4** and **6** represent the first reported examples of Tm­(III) and Lu­(III) hydroxo clusters, respectively, featuring a sandglass-like topology. Moreover, compound **4** is the first Lu­(III) complex reported with a nuclearity greater than five. Achieving isostructural motifs across the M^RE^(III) series is uncommon due to the lanthanide contraction effect, which causes a gradual decrease in the ionic radii and alters coordination preferences. The consistent formation of nonanuclear clusters in **1**–**6** is attributed to the templating effect of the {M^RE^(μ_3_-OH)_8_} unit, which robustly supports the central sandglass motif despite variations in 8-coordinate M^RE^(III) ionic radii from 1.206 Å for Eu­(III) to 1.117 Å for Lu­(III).^88^

Continuous shape measurements (CShM) of the coordination environments in **1**–**6** revealed that the central M^RE^1 atoms exhibit minimal deviation from an ideal square antiprismatic geometry, with shape parameter values *S*(SAPR) ranging from 0.025 to 0.067.^89^ In contrast, the eight peripheral M^RE^(III) centers display more significant distortions from ideal biaugmented trigonal prismatic geometry, as indicated by the *S*(BTPR) values of 1.842–2.411 for **1**, 1.774–2.421 for **2**, 1.735–2.617 for **3**, 1.792–2.295 for **4**, 1.497–2.475 for **5**, and 1.721–2.470 for **6** (Table S2).

The M^RE^–(μ_3_-OH) bond lengths range from 2.262(5) to 2.407(5) Å in **1**, 2.321(3) to 2.455(3) Å in **2**, 2.278(4) to 2.420(4) Å in **3**, 2.241(6) to 2.380(5) Å in **4**, 2.224­(6) to 2.383(6) Å in **5**, and 2.208(8) to 2.379(8) Å in **6** and are approximately 0.15–0.36 Å shorter than the corresponding M^RE^–(μ_4_-OH) distances (2.497(6)–2.594(6) Å in **1**, 2.564(4)–2.680(3) Å in **2**, 2.520(4)–2.572(4) Å in **3**, 2.481(6)–2.532(5) Å in **4**, 2.469(7)–2.532(7) Å in **5**, and 2.447(8)–2.544(8) Å in **6**). Both types of interactions exhibit bond lengths comparable to those reported for structurally related hydroxo clusters such as [Y_12_(μ_3_-OH)_16_(dmpa)_8_(H_2_O)_12_]­(ClO4)_12_ (dmpa^–^ = 3-hydroxy-2-(hydroxymethyl)-2-methylpropanoato),^90^ [Eu_16_(μ_6_-O)_2_(μ_3_-OH)_24_(tfac)_20_(MeOH)_8_] (tfac^–^ = 1,1,1-trifluoroacetylacetonato),^91^ [Dy_17_(μ_4_-O)­(μ_4_-OH)­(μ_3_-OH)_16_(dcd)_16_(H_2_O)_8_] (dcd^2–^ = 3,3-dimethylcyclopropane-1,2-dicarboxylato),^92^ [Tm_14_(μ_4_-OH)_2_(μ_3_-OH)_16_(*o*np)_24_] (*o*np^–^ = *o*-nitrophenolato),^93^ [Yb_5_(μ_4_-OH)­(μ_3_-OH)_4_(Iphacac)_10_] (Iphacac^–^ = 1,3-bis­(4-iodophenyl)-1,3-propanedionato),^94^ and [Lu_5_(μ_4_-OH)­(μ_3_-OH)_4_(phacac)_10_] (phacac^–^ = 1,3-diphenylpropane-1,3-dionato).^95^ The coordination modes of the sal-R ligands in **1**–**6**, along with the M^RE^–O_(sal‑R)_ distances 2.243(6)–2.409(6) Å for **1**, 2.285(4)–2.456(4) Å for **2**, 2.233(4)–2.412(4) Å for **3**, 2.212(6)–2.374(6) Å for **4**, 2.200(7)–2.373(7) Å for **5**, and 2.213(9)–2.398(8) Å for **6** are comparable to those typically found in M^RE^(III) β-diketonate complexes.^39,96,97^ The nonbonding M^RE^···M^RE^ distances within the basal planes of the pyramidal motifs range from 3.549(2) to 3.598(2) Å for **1**, 3.651(2) to 3.680(2) Å for **2**, 3.562(2) to 3.595(2) Å for **3**, 3.513(2) to 3.536(2) Å for **4**, 3.507(3) to 3.526(2) Å for **5**, and 3.461(4) to 3.507(3) Å for **6**. These values are significantly shorter than the corresponding distances between the basal M^RE^ atoms and the central M^RE^ atom located at the apex: 3.644(2)–3.665(2) Å for **1**, 3.703(2)–3.739(2) Å for **2**, 3.640(2)–3.666(2) Å for **3**, 3.602(2)–3.608(2) Å for **4**, 3.588(2)–3.614(2) Å for **5**, and 3.572(2)–3.617(3) Å for **6**.

The obtained crystalline materials **1**–**6** were also studied by powder XRD (PXRD; Figures S6–S8). The observed differences between the PXRD patterns of compounds **1**–**6**, simulated from single-crystal data collected at −173 or −123 °C, and those of powder samples measured at room temperature may be attributed to phase transitions, preferred orientation effects, or solvent loss.

Analytical methods, infrared (IR) spectroscopy, ^1^H, and ^13^C NMR spectroscopy were used to characterize **1**–**6** (Figures S9–S18). ^1^H-diffusion-ordered spectroscopy (^1^H-DOSY) NMR analysis of **1** and **6** confirmed the presence of nonanuclear species in solution, with estimated formula weights and hydrodynamic radii (**1**: FW = 3513 g/mol, rH = 13.05 Å; **6**: FW = 4250 g/mol, rH = 13.9 Å), which correlate well with data from X-ray structures (Figures S19 and S20). Fourier transform infrared-attenuated total reflection (FTIR-ATR) spectra presented in Figure 2 reveal that all compounds share similar transmittance profiles. Broad absorptions in the 3600–3030 cm^–1^ range correspond to ν­(OH) stretching vibrations, while δ­(OH) bending modes appear at 1341–1338 and 1325–1320 cm^–1^. The characteristic vibrational bands of the sal-R ligands include ν­(C=O) at 1666–1661 and 1636–1633 cm^–1^; ν­(C=C) at 1601–1599, 1545–1542, 1472–1469, 1451–1448, and 1436–1435 cm^–1^; ν­(C­(=O)–O) at 1232–1226 cm^–1^; ρ­(CH_3_, C_2_H_5_) at 1157–1155 cm^–1^; δ­(C_Ar_–H) at 1142–1140 and 1034–1032 cm^–1^; ν­(O–CH_3_/C_2_H_5_) at 1088–1086 cm^–1^; ν­(C_Ar_–H) at 956–950 cm^–1^; γ­(C_Ar_–H) at 868–866, 826–823, and 799–796 cm^–1^; ν­(Ar) at 755–752 cm^–1^; γ­(Ar) at 706–705 and 533–532 cm^–1^; δ­(Ar­(X)) at 661–660 cm^–1^; and δ­(Ph) at 561–555 cm^–1^. Chelate ring deformations (δ ring) and metal–ligand stretching vibrations ν­(M^RE^–O) are observed in the low-frequency region at 589–585, 475–451, and 425–408 cm^–1^.

**Figure 2:**
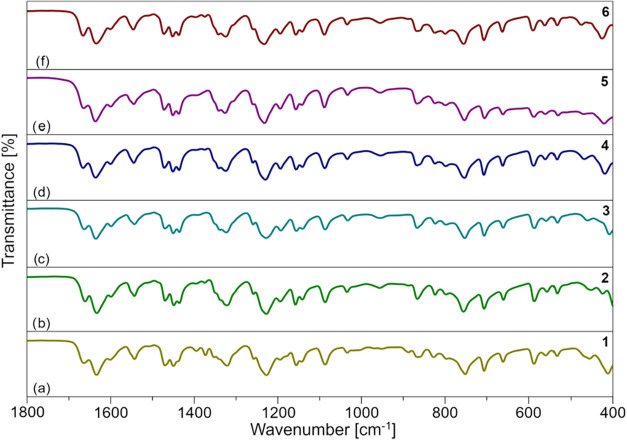
Comparison of FTIR-ATR spectra of **1**–**6** in the range of 1800–400 cm^–1^ (**1** (a), **2** (b), **3** (c), **4** (d), **5** (e), and **6** (f)).

### Synthesis of Molecular Precursors for the Preparation of Alkaline-Earth Manganites: Powder X-ray Diffraction and Transmission Electron Microscopy (TEM) Studies of the Resulting Materials

To synthesize alkaline-earth manganites, we employed molecular precursors containing equimolar amounts of Ca­(or Ba) and Mn. Among the resulting compounds, CaMnO_3_ and BaMnO_3_ are of particular interest due to their wide range of functional applications. These perovskite-type oxides have been extensively investigated as supercapacitors,^98^ catalysts for the oxygen evolution reaction (OER),^99^ photocatalysts for the degradation of air and water pollutants,^100^ and materials for high-temperature thermochemical heat storage.^101^ Since manganese oxides, such as pyrolusite (MnO_2_), manganite (MnOOH), and hausmannite (Mn_3_O_4_) exhibit high sorption capacities for actinides like uranium, neptunium, and plutonium, alkaline-earth manganites also show promise as matrix materials for the immobilization of radioactive waste.^102,103^

The alkaline-earth precursors [Ca­(sal-Et)_2_]*_n_* (**7**, 87%) and [Ba­(sal-Et)_2_(THF)]*_n_* (**8**, 76%) were synthesized by direct reaction of metallic Ca or Ba with 2 mol equiv of Hsal-Me in EtOH or EtOH/THF solution, as shown in Scheme 2 (Figures 3 and 4).

**Figure 3:**
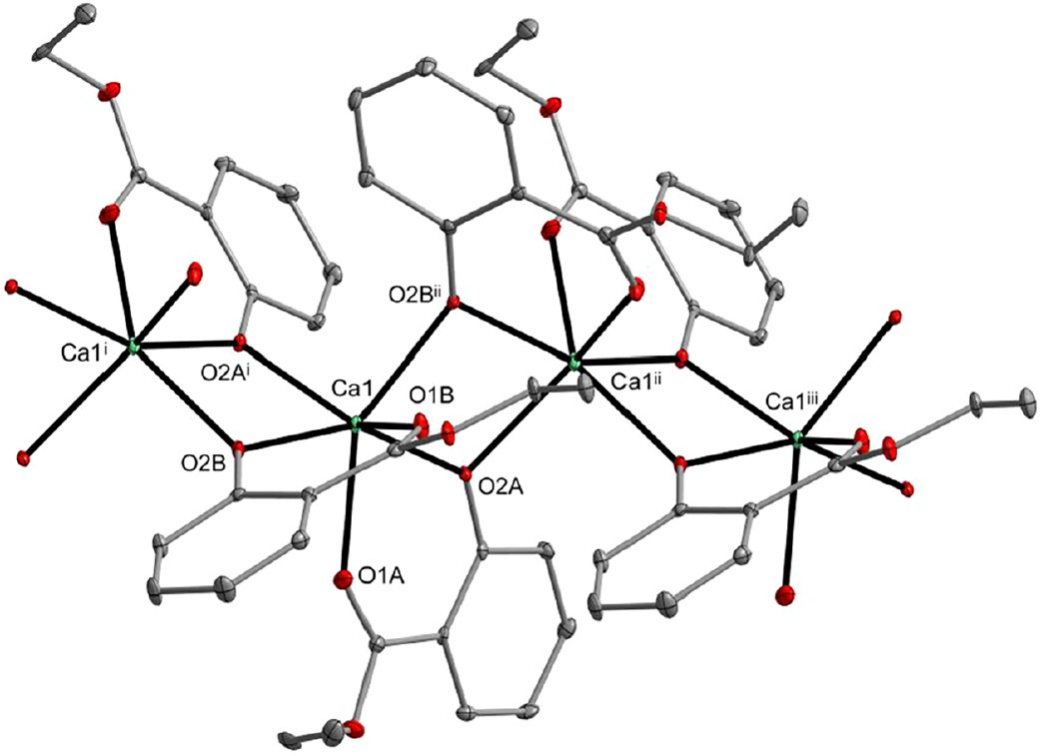
Molecular structure of [Ca­(sal-Et)_2_]*_n_* (**7**). Displacement ellipsoids are drawn at the 25% probability level. Hydrogen atoms are omitted for clarity [symmetry codes: (i) *x*, 1.5 – *y*, −1/2 + *z*; (ii) *x*, 1.5 – *y*, 1/2 + *z*; and (iii) *x*, *y*, 1 + *z*].

**Figure 4:**
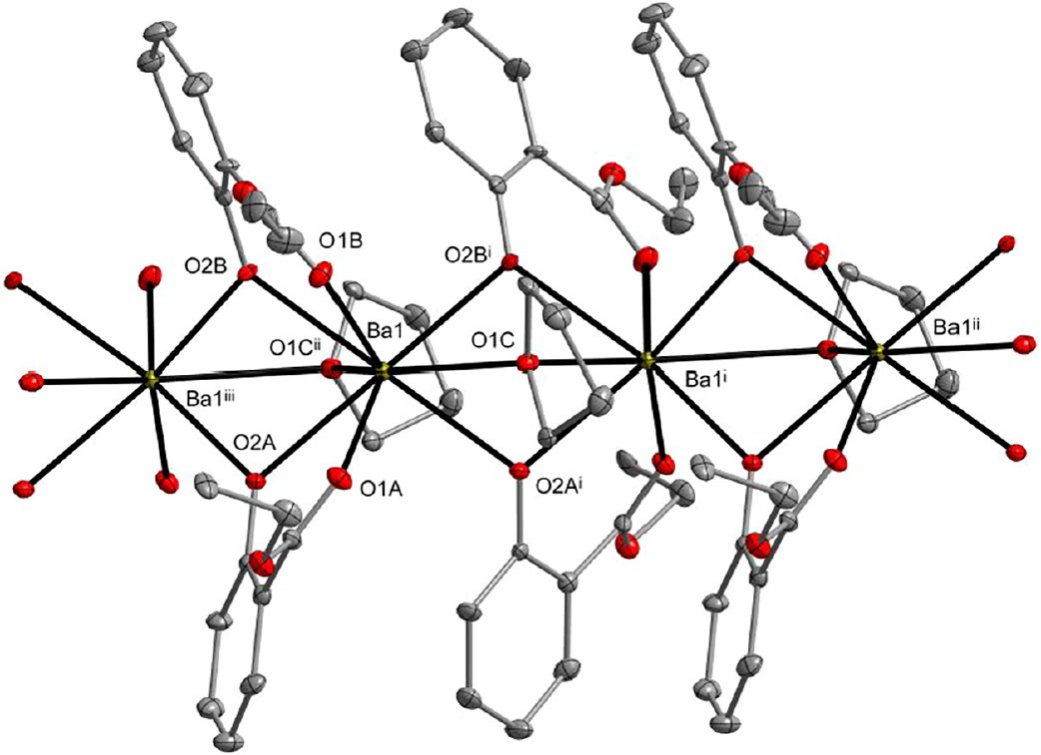
Molecular structure of [Ba­(sal-Et)_2_(THF)]*_n_* (**8**). Displacement ellipsoids are drawn at the 20% probability level. Hydrogen atoms are omitted for clarity [symmetry codes: (i) 1/2 – *x*, 1/2 + *y*, *z*; (iii) *x*, 1 + *y*, *z*; and (iii) 1/2 – *x*, −1/2 + *y*, *z*].

**Scheme 2:**
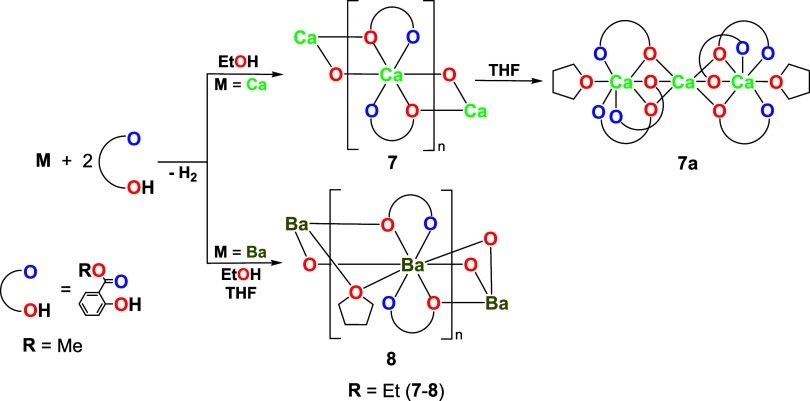
Synthesis of **7**–**8**

The molecular structures of **7** and **8** reveal the formation of 1D coordination polymers in which the M­(II) atoms are bridged by aryloxo oxygen atoms of the sal-Et ligands. In compound **7**, the Ca1 atom is coordinated by six oxygen donor atoms, adopting a highly distorted trigonal prismatic geometry. In compound **8**, the Ba1 atom is surrounded by eight oxygen donor atoms and adopts a distorted biaugmented trigonal prismatic geometry (Table S3).

These structures are notable in the field of structural chemistry, as compound **7** represents the first and compound **8** represents the third example of Ca­(Ba) aryloxides forming coordination polymer networks. Calcium coordination polymers are usually formed using carboxylate ligands, such as in [Ca_5_(ip)_5_(μ-H_2_O)_2_(H_2_O)_7_]*_n_* (ip^2–^ = isophthalato),^104^ [M­(sal)_2_(phen)_*x*_]*_n_* (M­(II) = Ca, Ba; *x* = 1, 2).^105^ In contrast, barium coordination polymers with phenolate ligands are rare, with only two reported examples: [Ba_2_(sdph)_2_(μ-H_2_O)­(MeOH)_2_]*_n_* (sdph^2–^ = 4,4′-sulfonyldiphenolato),^106^ and [Ba_2_(dhtnph)_2_(OH)_2_(H_2_O)_2_]*_n_* (dhtnph^–^ = 3,5-dihydroxy-2,4,6-trinitrophenolato).^107^

Upon treatment with THF, compound **7** undergoes disaggregation to yield [Ca_3_(sal-Et)_6_(THF)_2_] (**7a**, 84%) (Scheme 2 and Figure S21). A comparable structure has been previously reported only for [Ca_3_(mccp)_6_(THF)_2_] (mccp^–^ = 2-(methoxycarbonyl)­cyclopent-1-en-1-olato).^108^ Similarly, when the synthesis of **7** was carried out in an EtOH/THF solvent mixture, the formation of **7a** was also observed. In such cases, compound **7** was isolated as a crystalline precipitate, while **7a** remained in the solution. Powder XRD measurements of the resulting solids (Figure 5) confirmed the presence of both products within the reaction mixture.

**Figure 5:**
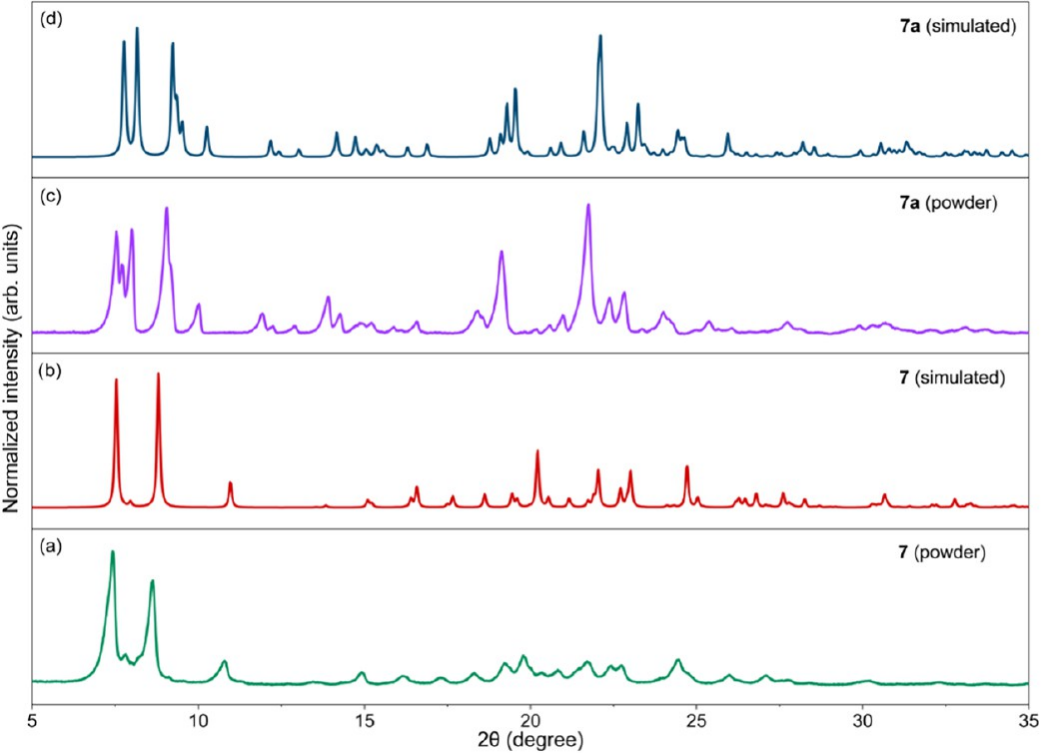
Comparison of PXRD patterns of powders obtained from the reaction of Ca with Hsal-Me in EtOH/THF (a, c) with reference patterns of compounds **7** and **7a** simulated from their crystal structures (b, d).

[Mn_2_(μ-OMe)_2_(sal-Me)_4_] (**9**, 67%) was synthesized via the reaction of 1 equiv of MnCl_2_ with 2 equiv of the sodium salt of methyl salicylate in a mixed MeOH/THF solution (Figure 6). In **9**, the two octahedrally coordinated Mn­(III) centers are bridged by two OMe ligands. This structural motif is characteristic of bisphenolato-alkoxide-bridged Mn­(III) dimers.^109,110^ Compounds **7**–**9** were characterized by FTIR-ATR, ^1^H, and ^13^C NMR spectroscopy (Figures S22–S31).

**Figure 6:**
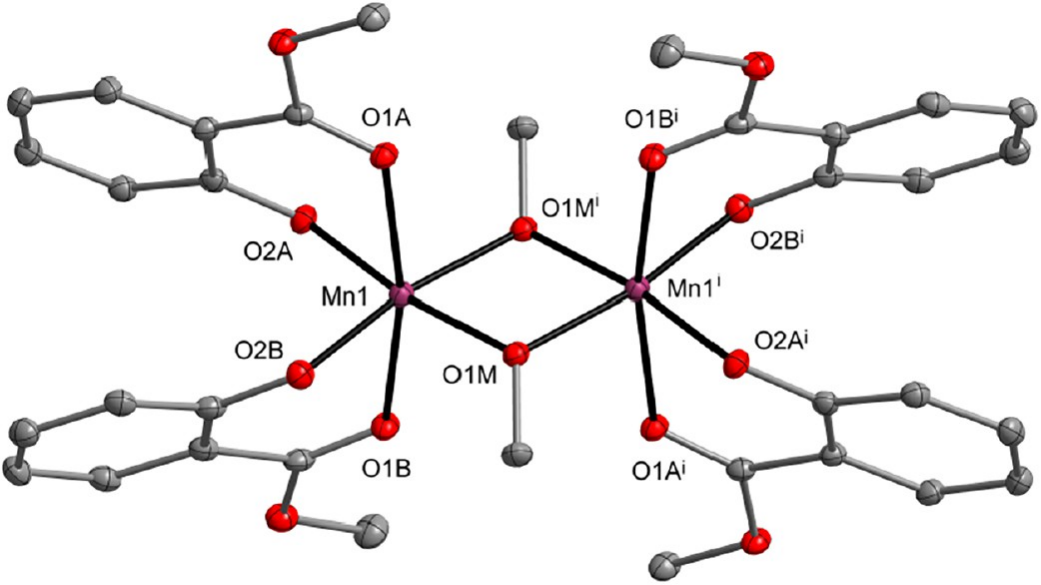
Molecular structure of [Mn_2_(μ-OMe)_2_(sal-Me)_4_] (**9**). Displacement ellipsoids are drawn at the 20% probability level. Hydrogen atoms are omitted for clarity [symmetry code: (i) 1 – *x*, 1 – *y*, 1 – *z*].

Thermogravimetric analysis and differential scanning calorimetry (TGA-DSC) were employed to investigate the thermal decomposition behavior of compounds **1**–**9** over the temperature range of 30–1000 °C, using a heating rate of 5 °C/min under a nitrogen (N_2_) atmosphere (Figures 7, S32, and S33). The TGA profiles of **1**–**6** are comparable, indicating similar and continuous thermal decomposition pathways for all six isostructural complexes.

**Figure 7:**
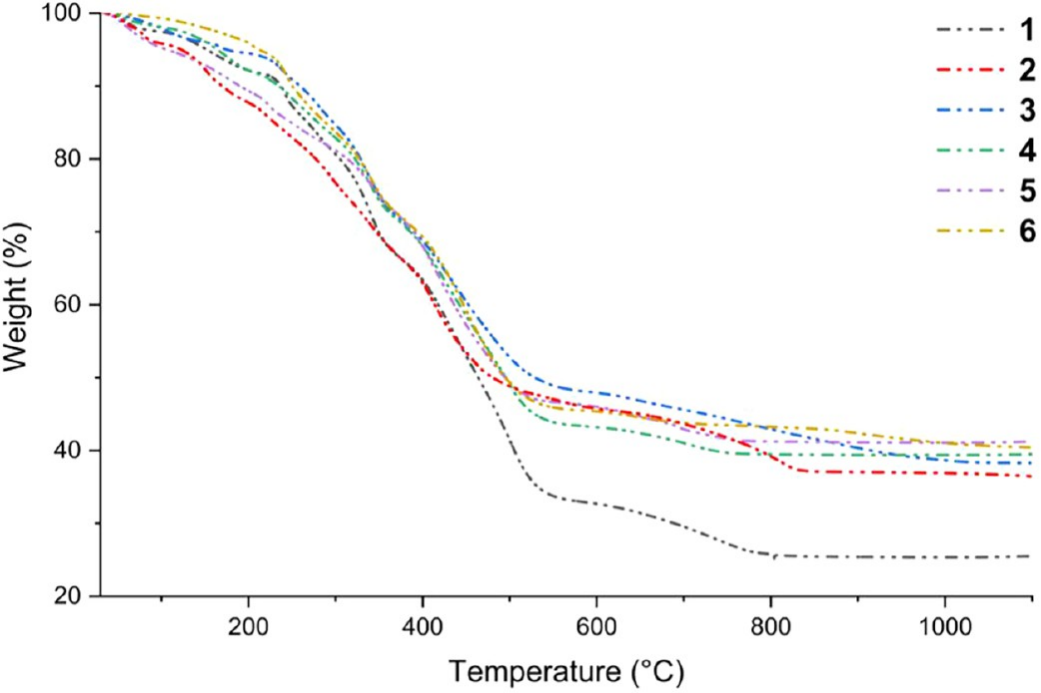
TGA curves of **1**–**6** recorded under a nitrogen atmosphere at a heating rate of 5 °C min^–1^ over the temperature range of 30–1100 °C.

The decomposition process begins with the loss of residual solvents (MeOH, EtOH, and THF) from the dried single crystals of **1**–**6**, occurring between 30 and 100 °C and corresponding to a mass loss of less than 5%. Water molecules present in the crystal lattice facilitate hydrolysis of the ester-type ligands, leading to the elimination of MeOH/EtOH or evaporation of the protonated ligands (Hsal-R) between 110 and 190 °C. Decomposition of the aromatic moieties of the ligands occurs between 200 and 530–560 °C, resulting in the formation of intermediate rare-earth oxyhydroxide phases (M^RE^O­(OH)). Subsequent weight loss of 4–10% at higher temperatures corresponds to the decomposition of M^RE^O­(OH) into M_2_^RE^O_3_. DSC analysis revealed crystallization of monoclinic, cubic, or mixed monoclinic/cubic M_2_^RE^O_3_ phases above 660 °C. Thermal decomposition of compounds **7**–**9** led to the formation of CaO, BaCO_3_, or Mn_2_O_3_ phases, with final degradation temperatures of 894 °C for **7** and 993 °C for **8**. In the case of compound **9**, the observed weight loss above 900 °C is attributed to the transformation of Mn_2_O_3_ into Mn_3_O_4_.

The thermal decomposition of compound **7** or **8** with **9**, using a Ca­(Ba)/Mn molar ratio of 1:1, was explored as a general strategy for synthesizing alkaline-earth manganites. PXRD analysis of the oxide materials obtained by calcining **7** and **9** at 850 °C revealed the formation of heterometallic oxides, primarily CaMnO_3_ and Ca_2_Mn_3_O_8_ (Figure 8a).

**Figure 8:**
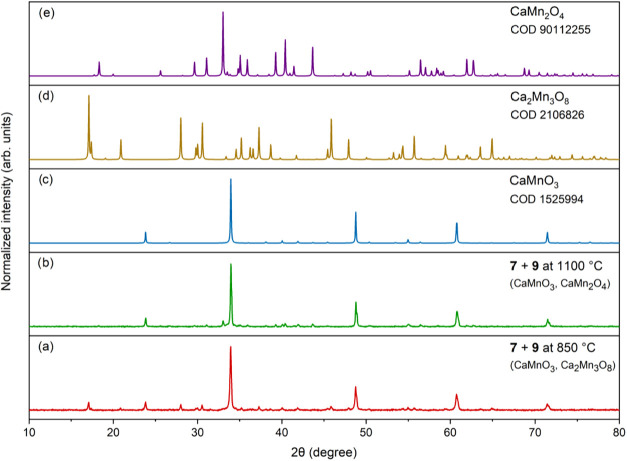
PXRD patterns of oxide materials obtained by calcination of **7** and **9** at 850 °C (a) and 1100 °C (b), compared with reference patterns for CaMnO_3_ [COD 1525994] (c), Ca_2_Mn_3_O_8_ [COD 2106826] (d), and CaMn_2_O_4_ [COD 9012255] (e).

Upon increasing the sintering temperature to 1100 °C, the major crystalline phases observed were CaMnO_3_ and CaMn_2_O_4_ (Figure 8b). The hexagonal BaMnO_3_ phase was obtained by the thermal decomposition of precursors **8** and **9** at both 850 °C (Figure 9a) and 1100 °C (Figure 9b).

**Figure 9:**
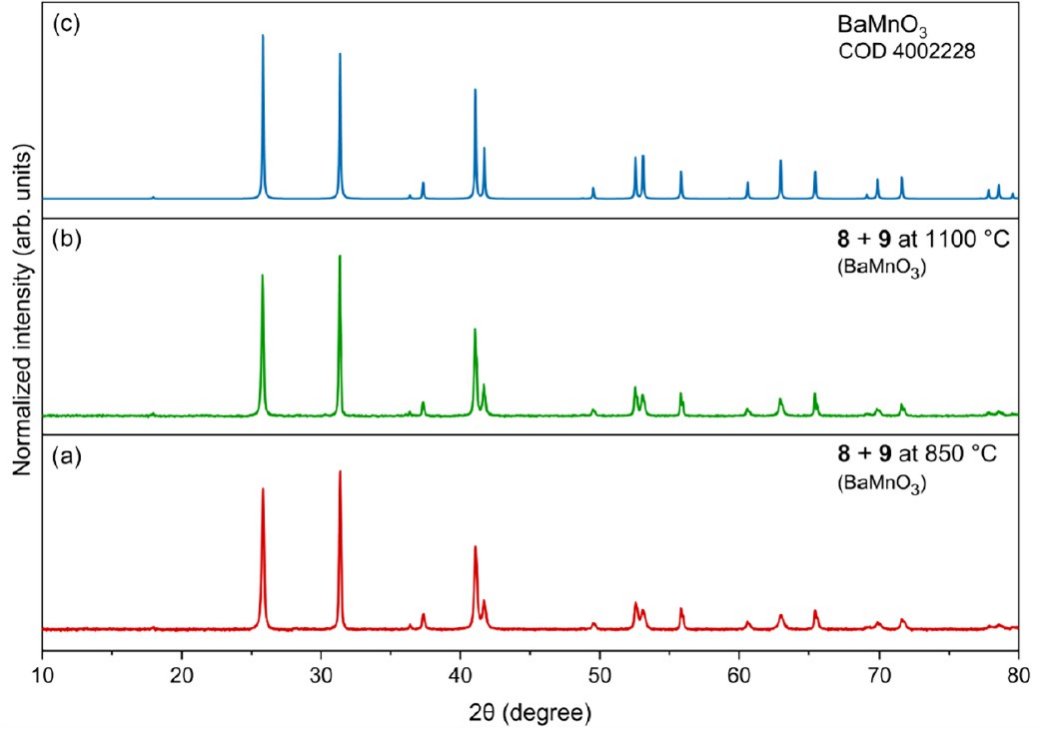
PXRD patterns of oxide materials obtained by calcination of **8** and **9** at 850 °C (a) and 1100 °C (b), compared with the reference pattern for BaMnO_3_ [COD 4002228] (c).

Subsequently, clusters **1**–**6** were introduced in 0.22 mol % quantities relative to Mn­(III) to prepare M^RE^(III)-doped heterometallic oxide nanomaterials via thermal decomposition of **7**–**8** and **9** at 1100 °C. PXRD patterns of the M^RE^(III)-doped Ba–Mn samples primarily show BaMnO_3_, with additional weak planes corresponding to a secondary Ba_6_Mn_24_O_48_ phase. When **7** and **9** were cocalcined with clusters **1**–**6**, the dominant phase was CaMnO_3_, accompanied by variable amounts of CaMn_2_O_4_, Ca_3_Mn_2_O_7_, and Mn_3_O_4_ (Figure 10; see Figures S34–S45). In Y-, Eu-, Yb-, and Lu­(III)-doped samples, minor quantities of CaCO_3_ and Ca_2_MnO_4_ were also detected. Increasing the M^RE^(III) ion concentration to 10 mol % led to CaMnO_3_ remaining as the primary phase, with CaMn_2_O_4_ and Mn_3_O_4_ appearing as secondary phases (Figures S41 and S44).

**Figure 10:**
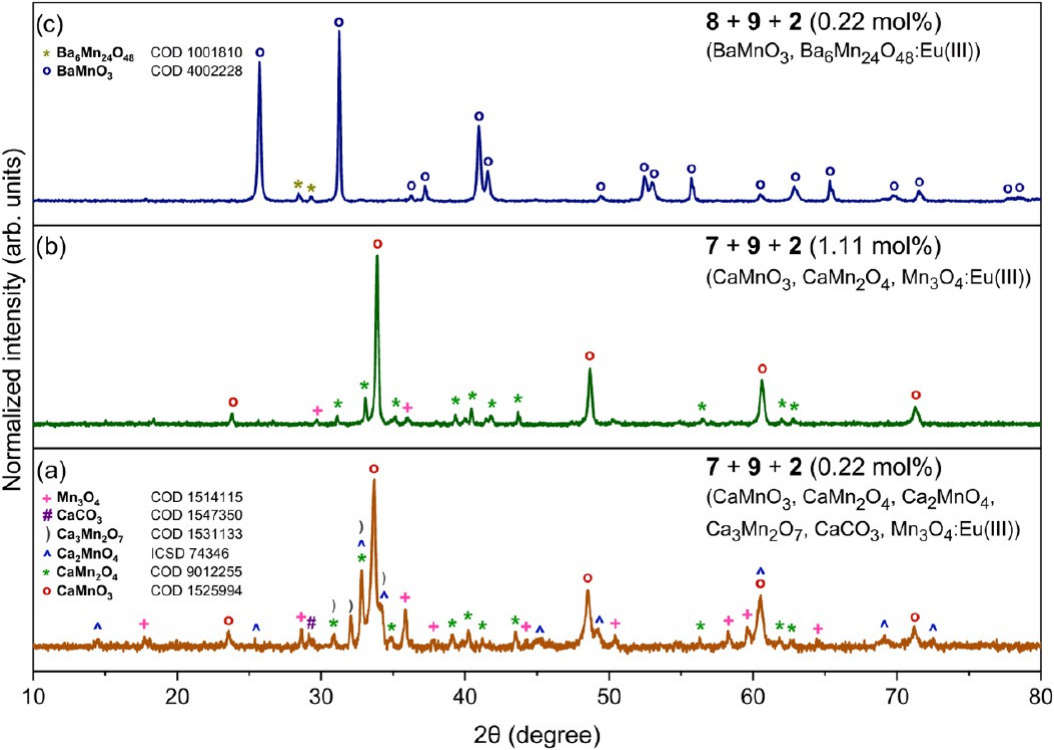
PXRD patterns of oxide materials obtained by calcination at 1100 °C: (a) **7** with **9** in the presence of 0.22 mol % compound **2**; (b) **7** with **9** in the presence of 1.11 mol % compound **2**; and (c) **8** with **9** in the presence of 0.22 mol % compound **2**.

### TEM Characterization of the Obtained Oxide Materials

The morphology and chemical composition of the synthesized oxide materials were investigated by using transmission electron microscopy (TEM). TEM analysis of the sample obtained by sintering compounds **7** and **9** at 1100 °C revealed the presence of two distinct types of particles: oval-shaped CaMnO_3_ and nanorods of CaMn_2_O_4_, with diameters ranging from 48 to 277 nm (Figures 11a–c, S46, and S47). In the case of BaMnO_3_, derived from the thermal treatment of compounds **8** and **9** at 1100 °C, aggregated oval nanoparticles with diameters between 176 and 290 nm were observed (Figures 11d–f and S48).

**Figure 11:**
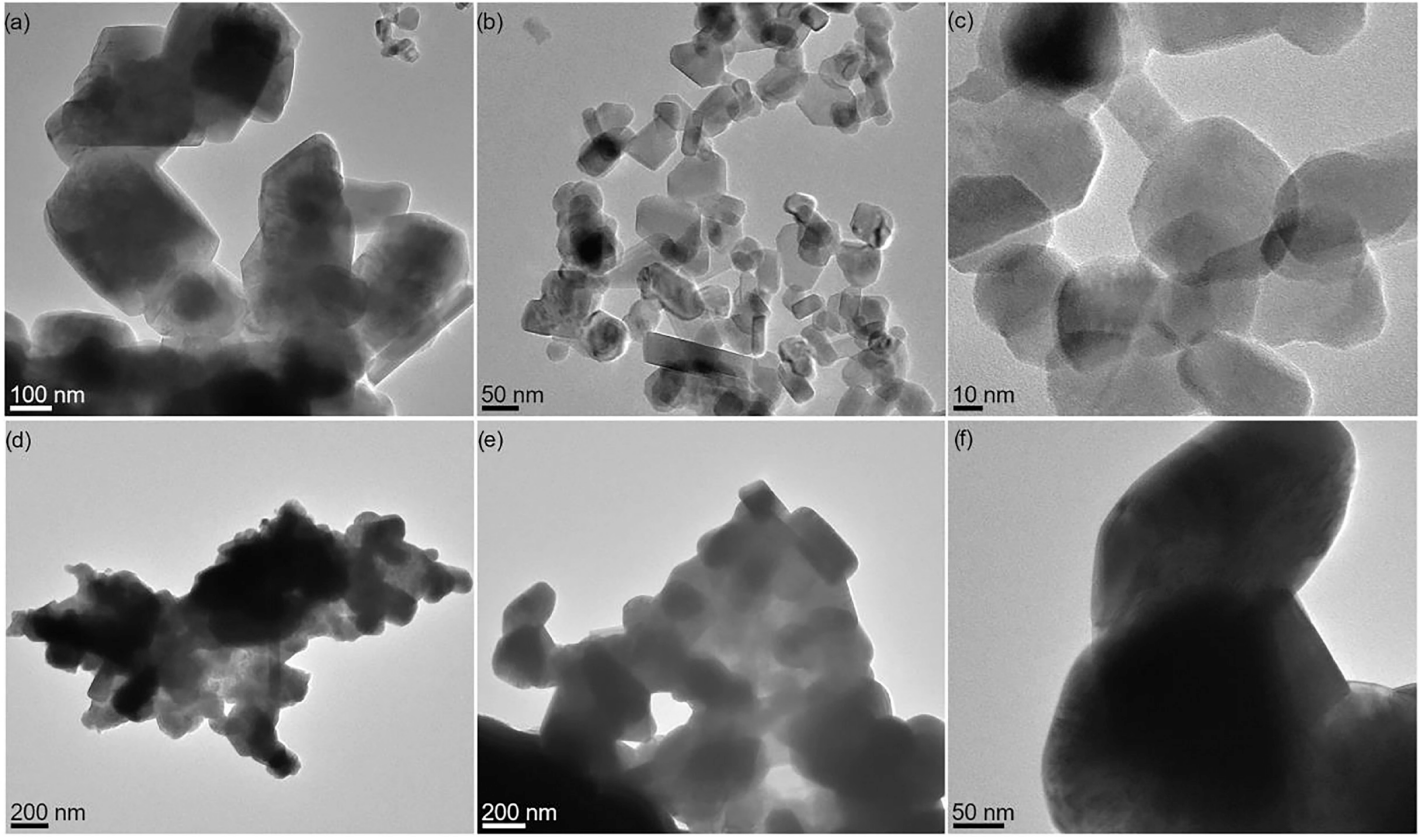
TEM images of oxide materials obtained by calcination of **7** with **9** (CaMnO_3_ and CaMn_2_O_4_, a–c) and **8** with **9** (BaMnO_3_, d–f) at 1100 °C.

TEM images of the oxide materials obtained by thermal decomposition of compounds **7** and **9** in the presence of 0.22 mol % clusters **1**–**6**, supported by energy-dispersive X-ray (EDX) analysis, confirm the formation of M^RE^(III)-doped CaMnO_3_ and CaMn_2_O_4_ crystallites (Figure 12a–c). These crystallites exhibit shapes, sizes, and morphologies comparable to those observed in the undoped materials. Nanoparticles of residual calcium-containing minor phases, detected by PXRD, were generally not visible in TEM images, likely due to coalescence with the dominant CaMnO_3_ and CaMn_2_O_4_ phases. TEM investigation of the materials derived from the decomposition of compounds **8** and **9** with 0.22 mol % clusters **1**–**6** revealed the formation of highly aggregated, oval, or irregularly shaped M^RE^(III)-doped BaMnO_3_ nanoparticles, alongside significantly smaller M^RE^MnO_3_ nanorods (Figures 12d–f and S49–S81). A representative EDX spectrum of an isolated rod-like crystallite (Figure 13) confirms the elemental composition consistent with EuMnO_3_ perovskite.

**Figure 12:**
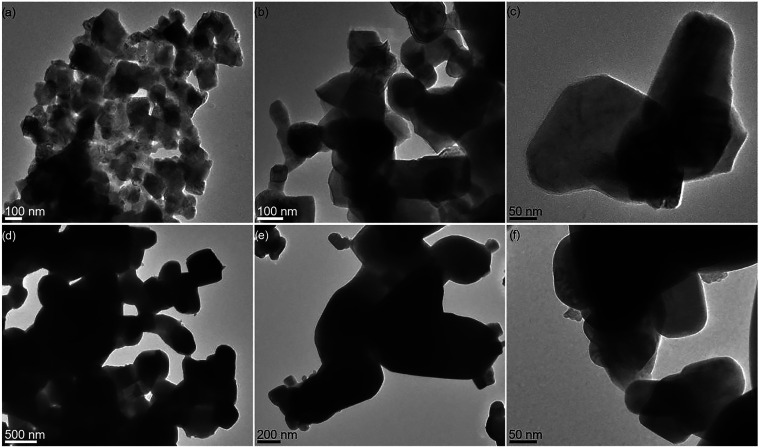
TEM images of oxide materials obtained by calcination at 1100 °C of **7** or **8** with **9** in the presence of 0.22 mol % **2**: (a–c) Eu­(III)-doped CaMnO_3_ and CaMn_2_O_4_ and (d–f) Eu­(III)-doped BaMnO_3_.

**Figure 13:**
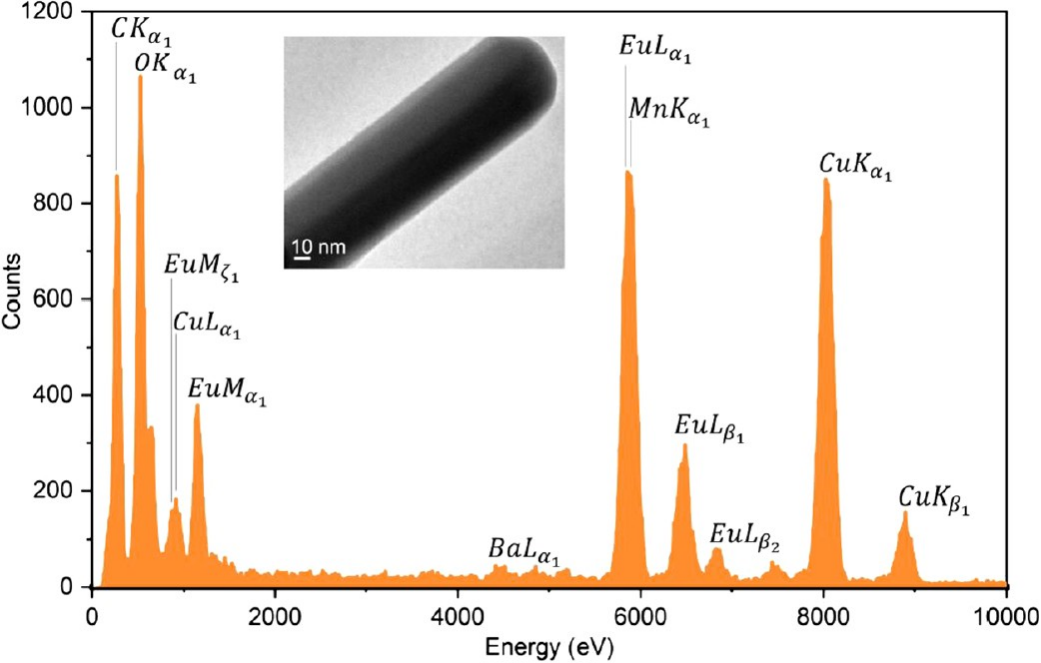
EDX spectrum of a EuMnO_3_ rod-like crystallite identified in the Eu­(III)-doped BaMnO_3_ material obtained by calcination of **8** with **9** in the presence of 0.22 mol % **2** at 1100 °C. The detected copper and carbon signals originate from the copper–carbon TEM grid.

PXRD and TEM studies revealed that the alkaline-earth manganite phases CaMnO_3_/CaMn_2_O_4_ and BaMnO_3_, formed via thermal decomposition of precursors **7** or **8** with **9**, can serve as effective host matrices for the immobilization of high-level nuclear waste. The resulting materials were capable of incorporating Eu­(III) ions, used as chemical surrogates for Cm­(III)/Am­(III), by substituting them at the Ca­(II)/Ba­(II) lattice sites. In the case of BaMnO_3_, employed as a matrix for Eu­(III) ion immobilization, TEM analysis confirmed the formation of nanorod-like crystallites corresponding to EuMnO_3_ phases.

## Conclusions

In this work, a series of rare-earth molecular cluster aggregates with the general formula [M_9_^RE^(μ_4_-OH)_2_(μ_3_-OH)_8_(sal-R)_16_]­X (M^RE^ = Y (**1**), Eu (**2**), Dy (**3**), Tm (**4**), Yb (**5**), Lu (**6**); X^–^ = Cl^–^ (**1**–**2**, **4**–**6**), or DyCl_4_^–^ (**3**); R = Me (**2**, **4**–**6**), Et (**3**), Me and Et (**1**)) were synthesized and investigated as surrogates for minor actinides present in high-level waste (HLW) generated from nuclear fuel reprocessing. A straightforward and efficient method is described for HLW immobilization using stable, insoluble alkaline-earth manganite solid-state matrices derived from molecular precursors. These materials were obtained via thermal decomposition of mixtures of either [Ca­(sal-Et)_2_]*_n_* (**7**) or [Ba­(sal-Et)_2_(THF)]*_n_* (**8**) with [Mn_2_(μ-OMe)_2_(sal-Me)_4_] (**9**) at 850 or 1100 °C (Ca­(Ba):Mn = 1:1), in the presence of 0.22 or 1.11 mol % clusters **1**–**6**. The resulting heterometallic oxide phases CaMnO_3_/CaMn_2_O_4_ and BaMnO_3_ demonstrated efficient incorporation of M^RE^(III) ions at the Ca­(II)/Ba­(II) lattice sites, underscoring their suitability as HLW host matrices. Notably, the formation of M^RE^MnO_3_ crystallites was observed during the synthesis of M^RE^(III)-doped BaMnO_3_. This study represents the first report on the use of alkaline-earth manganites for the immobilization of radioactive waste. Owing to the widespread availability and low cost of manganese compounds frequently encountered as waste in the electrochemical industry, this method also offers promising economic viability.

## Experimental Section

### Materials and Methods

All syntheses were conducted under a controlled nitrogen (N_2_) atmosphere by using standard Schlenk techniques. Solvents were purified by distillation: methanol and ethanol over metallic magnesium, and tetrahydrofuran (THF) over metallic sodium. Reagents were obtained from commercial suppliers: methyl salicylate, Li, Ca, Ba, MnCl_2_, EuCl_3_, DyCl_3_, TmCl_3_, and LuCl_3_ (Sigma-Aldrich); YCl_3_ and YbCl_3_ (Alfa Aesar); methanol (Pol-Aura); ethanol (POCH); diethyl glycol (Laborchemie Apolda); THF (Eurochem BGD); dimethyl sulfoxide (DMSO)-*d*_6_ and THF-*d*_8_ (Deutero GmbH). ^1^H and ^13^C NMR spectra were recorded at room temperature for **1**–**8**, or at 50 °C for **7a** with a JEOL JNM-ECZ 400 MHz spectrometer. Chemical shifts (δ) are reported in parts per million (ppm) relative to residual solvent signals. FTIR-ATR spectra were collected by using a Bruker Vertex 70 vacuum spectrometer with a resolution of 2 cm^–1^. Elemental analyses were performed on a PerkinElmer 2400 CHN elemental analyzer. Thermogravimetric and differential scanning calorimetry (TGA-DSC) measurements were conducted with a METTLER-TOLEDO TGA/DSC 3+ system under nitrogen (50 mL/min) at a heating rate of 5 °C min^–1^. Thermal decomposition of molecular precursors was carried out in air by using a Neotherm NT 1313 furnace equipped with a KXP4 thermostat. PXRD of the oxide materials was performed using a Malvern Panalytical Compact X-ray diffractometer (Aeris, 600 W). Phase identification was supported using reference patterns from the Crystallography Open Database (COD).^111^ Morphological analysis of metal oxides was performed using an FEI Tecnai G2 20 X-Twin transmission electron microscope (TEM) equipped with a field-emission gun (FEG) and an integrated energy-dispersive X-ray (EDX) spectrometer. For TEM observations, 200-mesh copper grids with lacey carbon films were used. As a result, copper and carbon signals from the grid are visible in the TEM-EDX spectra. Single-crystal X-ray diffraction data were collected on a XtaLAB Synergy R diffractometer (for compounds **1**, **4**, **5**, **6**, **7a**, **8**, and **9** at 100 K or compound **2** at 150 K) and an Xcalibur Ruby diffractometer (for compounds **3** and **7** at 100 K).^112^ Experimental parameters, crystal data, and refinement details are provided in Table S1. Structures were solved using direct methods and refined by full-matrix least-squares procedures on *F* ^2^ using the SHELXTL package.^113^ Nonhydrogen atoms were refined anisotropically. Hydrogen atoms were placed in geometrically calculated positions and included in the structure factor calculations but not refined. Crystals of compounds **2**, **3**, **5**, and **6** exhibited solvent-accessible voids; therefore, a SQUEEZE procedure was applied and included in the CIF files. Molecular graphics were generated using Diamond software (version 3.1e).^114^ Crystallographic data for compounds **1**–**9** have been deposited with the Cambridge Crystallographic Data Centre under accession numbers CCDC 2444425–2444434. These data are available free of charge from www.ccdc.cam.ac.uk/data_request/cif, by emailing data_request@ccdc.cam.ac.uk, or by contacting the CCDC at 12 Union Road, Cambridge CB2 1EZ, U.K.; Fax: + 44 1223 336033.

### Synthesis of [Y_9_(μ_4_-OH)_2_(μ_3_-OH)_8_(sal-R)_16_]Cl (**1**, R = Me (0.70), Et (0.30)), [Eu_9_(μ_4_-OH)_2_(μ_3_-OH)_8_(sal-Me)_16_]Cl (**2**), [Dy_9_(μ_4_-OH)_2_(μ_3_-OH)_8_(sal-Et)_16_]­[DyCl_4_] (**3**), [Tm_9_(μ_4_-OH)_2_(μ_3_-OH)_8_(sal-Me)_16_]Cl (**4**), [Yb_9_(μ_4_-OH)_2_(μ_3_-OH)_8_(sal-Me)_16_]Cl (**5**), and [Lu_9_(μ_4_-OH)_2_(μ_3_-OH)_8_(sal-Me)_16_]Cl (**6**)

To a 150 mL Schlenk flask equipped with a stir bar, methyl salicylate (0.65 mL; 5.0 mmol), THF (15 mL), and metallic lithium (0.0312 g; 4.5 mmol) were introduced. The mixture was stirred at room temperature for 2 h until lithium had fully reacted. Subsequently, M^RE^Cl_3_ (1.50 mmol; YCl_3_: 0.2937 g, EuCl_3_: 0.3870 g, DyCl_3_: 0.403 g, YbCl_3_: 0.4191 g, TmCl_3_: 0.5740 g, LuCl_3_: 0.4220 g) and 20–40 mL of methanol were added to the light yellow solution, and the reaction mixture was stirred for an additional 72 h. Due to the limited solubility of the products, particularly compounds **1** and **3**, 10–20 mL of EtOH was added, leading to the partial or complete transesterification of sal-Me ligand. After several days of crystallization at ambient conditions, colorless crystals of compounds **1**–**5** were collected by filtration, washed with hexane (3 × 10 mL), and dried under vacuum. Compound **6** was obtained as single crystals suitable for X-ray crystallography from the reaction between in situ generated [Zn_4_(sal-Me)_8_] (from ZnEt_2_ and Hsal-Me) and LuCl_3_.

### [Y_9_(μ_4_-OH)_2_(μ_3_-OH)_8_(sal-R)_16_]Cl (**1**, for R = Me (0.70), Et(0.30))

As a result of sal-Me ligand transesterification using EtOH, compound **1** contains ca. 9% sal-Et ligand as calculated by ^1^H NMR. Yield (**1**): 0.26 g (46%). C_128_H_122_Y_9_O_58_Cl (3423.91 g/mol): C, 44.90; H, 3.59; Cl, 1.04; found: C, 45.10; H, 3.65; Cl, 1.02. ^1^H NMR (400 MHz, DMSO-*d*_6_): δ 10.80–9.67 (8H, s, OH), 7.73–7.61 (8H, m, ArH), 7.19–7.00 (8H, m, ArH), 6.81–6.32 (32H, m, ArH), 6.11–6.01 (8H, m, ArH), 4.07 (4H, m, OCH_2_ (sal-Et)), 3.74, 3.58, 3.26, 2.59 (44H, m, OCH_3_), 3.06 (2H, s, OH), 1.24 (6H, m, CH_3_ (sal-Et)); *n*-hexane: 1.17, 0.77. ^13^C NMR (101 MHz, DMSO-*d*_6_): δ 170.68 (16C, C=O), 160.52 (16C, C–O), 134.87 (16C, ArH), 130.18 (16C, ArH), 122.50 (16C, ArH), 114.37 (16C, ArH), 111.76 (16C, Ar), 61.27, 60.04 (6C, OCH_2_), 51.26 (10C, OCH_3_), 14.01 (6C, CH_3_); *n*-hexane: 22.05, 13.88. FTIR-ATR (cm^–1^): 3583 (w), 3380 (m), 3279 (m), 3058 (w), 2954 (m), 2870 (w), 1664 (m), 1633 (vs), 1601 (m), 1542 (m), 1470 (m), 1449 (m), 1394 (m), 1373 (m), 1352 (m), 1320 (m), 1257 (m), 1226 (s), 1194 (m), 1155 (m), 1142 (m), 1086 (m), 1033 (m) 950 (w), 887 (w), 866 (m), 826 (m), 796 (m), 751 (s), 706 (m), 660 (m), 586 (m), 558 (m), 522 (m), 455 (m), 411 (m).

### [Eu_9_(μ_4_-OH)_2_(μ_3_-OH)_8_(sal-Me)_16_]Cl (**2**)

Yield (**2**): 0.38 g (57%). Anal. calcd for C_128_H_122_Eu_9_O_58_Cl (3991.44 g/mol): C, 38.52; H, 3.08; Cl, 0.89; found: C, 38.56; H, 3.10; Cl, 0.86. FTIR-ATR (cm^–1^): 3571 (w), 3198 (w), 3019 (w), 2951 (m), 2850 (m), 1661 (m), 1633 (vs), 1599 (m), 1543 (m), 1469 (m), 1448 (s), 1435 (m), 1395 (w), 1374 (w), 1321 (m), 1258 (m), 1227 (s), 1194 (m), 1157 (m), 1141 (m), 1086 (m), 1034 (m) 956 (w), 887 (w), 866 (m), 823 (m), 797 (m), 755 (s), 706 (m), 660 (m), 586 (m), 555 (m), 532 (m), 451 (w), 424 (m).

### [Dy_9_(μ_4_-OH)_2_(μ_3_-OH)_8_(sal-Et)_16_]­[DyCl_4_] (**3**)

As a result of complete sal-Me ligand transesterification using EtOH, compound **3** contains the sal-Et ligand. Yield (**3**): 0.49 g (64%). Anal. calcd for C_144_H_154_Dy_10_O_58_Cl_4_ (4579.54 g/mol): C, 37.77; H, 3.39; Cl, 3.10; found: C, 37.73; H, 3.40; Cl, 3.07. FTIR-ATR (cm^–1^): 3575 (vw), 3193 (w), 3019 (w), 2951 (w), 2850 (w), 2689 (w), 2323 (w), 1663 (m), 1635 (vs), 1599 (m), 1542 (m) 1470 (m), 1450 (m), 1435 (m), 1339 (m), 1322 (m), 1259 (m), 1228 (s), 1192 (m), 1155 (m), 1140 (m), 1086 (m), 1032 (m), 955 (w), 866 (m), 823 (m), 797 (m), 752 (s), 706 (m), 660 (m), 585 (m), 558 (m), 532 (m), 460 (w), 408 (m).

### [Tm_9_(μ_4_-OH)_2_(μ_3_-OH)_8_(sal-Me)_16_]­Cl (**4**)

Yield (**4**): 0.40 g (58%). Anal. calcd for C_128_H_122_Tm_9_O_58_Cl (4144.16 g/mol): C, 37.10; H, 2.97; Cl, 0.86; found: C, 37.16; H, 2.96; Cl, 0.84. FTIR-ATR (cm^–1^): 3582 (w), 3185 (m), 3061 (m), 3021 (m), 2951 (m), 1666 (m), 1636 (vs), 1600 (m), 1544 (m), 1471 (m), 1451 (m), 1436 (m), 1374 (vw), 1341 (m), 1324 (m), 1259 (m), 1229 (s), 1193 (m), 1156 (m), 1141 (m), 1087 (m), 1033 (m), 954 (m), 867 (m), 823 (m), 798 (m), 753 (s), 706 (m), 661 (m), 587 (m), 559 (m), 533 (m), 468 (m), 419 (m).

### [Yb_9_(μ_4_-OH)_2_(μ_3_-OH)_8_(sal-Me)_16_]­Cl (**5**)

Yield (**5**): 0.52 g (75%). Anal. calcd for C_128_H_122_Yb_9_O_58_Cl (4181.12 g/mol): C, 36.77; H, 2.94; Cl, 0.85; found: C, 36.81; H, 2.95; Cl, 0.82. FTIR-ATR (cm^–1^): 3379 (m), 3023 (w), 2952 (w), 1664 (m), 1636 (vs), 1599 (m), 1544 (m), 1472 (m), 1451 (m), 1436 (m), 1341 (m), 1325 (m), 1259 (m), 1231 (s), 1194 (m), 1156 (m), 1142 (m), 1088 (m), 1033 (w), 955 (w), 867 (m), 823 (w), 798 (w), 753 (m), 705 (m), 661 (m), 587 (m), 560 (w), 531 (w), 469 (vw), 420 (m).

### [Lu_9_(μ_4_-OH)_2_(μ_3_-OH)_8_(sal-Me)_16_]Cl (**6**)

To a solution of methyl salicylate (0.65 mL, 5.0 mmol) in THF (15 mL) in a 150 mL Schlenk flask was added ZnEt_2_ (2.0 mmol, 2 mL) dropwise under a nitrogen atmosphere. The reaction mixture was stirred at room temperature for 3 h. Subsequently, LuCl_3_ (0.3870 g, 1.38 mmol) and methanol (25 mL) were added, and the mixture was stirred for an additional 72 h. The resulting solution was left undisturbed to crystallize for 3 weeks. Colorless crystals of compound **6** were collected by filtration, washed with hexane (3 × 10 mL), and dried under vacuum. Yield (**6**): 0.28 g (43%). Anal. calcd for C_128_H_122_Lu_9_O_58_Cl (4198.45 g/mol): C, 36.62; H, 2.93; Cl, 0.84; found: C, 36.81; H, 2.95; Cl, 0.82. ^1^H NMR (400 MHz, DMSO-*d*_6_): δ 8.41–8.22 (8H, s, OH), 7.72 (8H, m, ArH), 7.00 (8H, m, ArH), 6.87 (8H, m, ArH), 6.65 (16H, m, ArH), 6.45 (8H, m, ArH), 6.13 (8H, m, ArH), 6.05 (8H, m, ArH), 3.31 (2H, s, OH), 3.21 (24H, s, OCH_3_), 2.58 (24H, s, OCH_3_). ^13^C NMR (101 MHz, DMSO-*d*_6_): δ 171.11, 171.05 (16C, C=O), 169.72, 167.45 (16C, C–O), 136.07, 133.75 (16C, ArH), 130.74 (16C, ArH), 121.99 (16C, ArH), 117.01, 115.29 (16C, Ar), 112.55 (16C, ArH), 52.67, 50.48 (16C, OCH_3_). FTIR-ATR (cm^–1^): 3585 (w), 3163 (w), 3020 (w), 2952 (m), 1665 (m), 1634 (vs), 1600 (m), 1545 (m), 1472 (m), 1451 (m), 1436 (m), 1396 (w), 1374 (w), 1342 (m), 1324 (m), 1259 (m), 1232 (s), 1194 (m), 1156 (m), 1141 (m), 1088 (m), 1034 (m), 955 (m), 867 (m), 824 (m), 799 (m), 754 (s), 706 (m), 662 (m), 589 (m), 561 (m), 532 (m), 475 (m), 425 (m).

### Synthesis of [Ca­(sal-Et)_2_]*_n_* (**7**)

In a 150 mL Schlenk flask equipped with a magnetic stir bar, metallic calcium (0.1964 g, 4.90 mmol), methyl salicylate (0.877 mL, 9.80 mmol), and ethanol (30 mL) were combined under a nitrogen atmosphere. The reaction mixture was stirred at room temperature for 96 h. Afterward, the light yellow solution was concentrated under vacuum and allowed to stand at room temperature for crystallization. After several days, colorless crystals of compound **7** were collected by filtration, washed with hexane (3 × 10 mL), and dried under vacuum. Due to transesterification of the sal-Me ligand, the final product contained the sal-Et ligand. Yield: 1.58 g (87%). Anal. calcd for C_18_H_18_CaO_6_: C, 58.37; H, 4.90; found: C, 58.43; H, 4.91. FTIR-ATR (cm^–1^): 3063 (w), 3022 (w), 2993 (w), 2947 (w), 2902 (w), 2849 (w), 2791 (vw), 2662 (vw), 1669 (vs), 1599 (m), 1545 (m), 1468 (m), 1446 (m), 1394 (w), 1371 (w), 1323 (s), 1260 (m), 1224 (vs), 1192 (m), 1159 (m), 1143 (m), 1083 (m), 1037 (w), 1019 (w), 959 (w), 888 (w), 865 (m), 820 (m), 798 (w), 765 (m), 755 (s), 708 (m), 660 (m), 579 (s), 559 (w), 536 (m), 465 (m), 441 (w), 421 (w).

### Synthesis of [Ca_3_(sal-Et)_6_(THF)_2_] (**7a**)

A 150 mL Schlenk flask equipped with a magnetic stir bar was charged with compound **7** (0.41 g, 1.106 mmol) and tetrahydrofuran (20 mL). The reaction mixture was stirred and refluxed under a nitrogen atmosphere for 72 h. After cooling, the solution was left undisturbed to allow for crystallization. After several days, colorless crystals of compound **7a** were collected by filtration, washed with hexane (3 × 10 mL), and dried under vacuum. Yield: 0.39 g (28%). Anal. calcd for C_62_H_70_Ca_3_O_20_: C, 59.31; H, 5.62; found: C, 59.39; H, 5.63. ^1^H NMR (400 MHz, THF-*d*_8_): δ 7.65 (6H, m, ArH), 6.84 (12H, m, ArH), 6.23 (6H, m, ArH), 4.12 (12H, m, OCH_2_), 3.61 (m, 8H, THF) 1.76 (m, 8H, THF), 1.24 (18H, m, CH_3_). ^13^C NMR (101 MHz, THF-*d*_8_): δ 171.35 (6C, C=O), 169.67 (6C, C–O), 135.31 (6C, ArH), 131.81 (6C, ArH), 125.08 (6C, ArH), 115.24 (6C, Ar), 113.25 (6C, ArH), 68.20 (4C, THF), 60.81 (6C, OCH_2_), 26.36 (4C, THF), 14.55 (6C, CH_3_). FTIR-ATR (cm^–1^): 3090 (vw), 3062 (vw), 3046 (vw), 3021 (w), 2981 (m), 2958 (m), 2928 (w), 2901 (w), 2872 (w), 2671 (w), 1685 (m), 1656 (vs), 1600 (m), 1540 (m), 1505 (w), 1467 (s), 1443 (m), 1392 (m), 1369 (m), 1347 (m), 1320 (m), 1259 (m), 1213 (s), 1181 (m), 1158 (m), 1115 (m), 1081 (m), 1039 (m), 1021 (m), 966 (w), 951 (w), 944 (w), 912 (w), 890 (m), 867 (m), 855 (m), 823 (m), 798 (m), 750 (m), 707 (m), 658 (m), 578 (s), 553 (m), 537 (m), 461 (m), 447 (m), 407 (m).

### Synthesis of [Ba­(sal-Et)_2_(THF)]*_n_* (**8**)

In a 150 mL Schlenk flask equipped with a magnetic stir bar, metallic barium (0.3159 g; 2.30 mmol), methyl salicylate (0.600 mL; 4.60 mmol), ethanol (30 mL), and tetrahydrofuran (20 mL) were added under a nitrogen atmosphere. The reaction mixture was stirred at room temperature for 96 h. After completion, the solution was concentrated under vacuum and left undisturbed at room temperature to allow crystallization. After several weeks, colorless crystals of compound **8** were collected by filtration, washed with hexane (3 × 10 mL), and dried under vacuum. Due to transesterification of the sal-Me ligand, the final product contained the sal-Et ligand. Yield: 0.94 g (76%). Anal. calcd for C_22_H_26_BaO_7_: C, 48.95; H, 4.86; found: C, 49.93; H, 4.85. ^1^H NMR (400 MHz, THF-*d*_8_): 7.78 (2H, m, ArH), 7.37 (2H, m, ArH), 6.87 (2H, m, ArH), 6.75 (2H, m, ArH), 4.33 (4H, m, OCH_2_), 3.57 (m, 8H, THF), 1.72 (m, 4H, THF), 1.34 (6H, s, CH_3_). ^13^C NMR (101 MHz, THF-*d*_8_) δ 170.84 (2C, C=O), 163.82 (2C, C–O), 136.08 (2C, ArH), 130.80 (2C, ArH), 121.35 (2C, ArH), 119.13 (1C, ArH), 113.86 (2C, Ar), 67.20 (2C, THF), 62.07 (2C, OCH_2_), 25.10 (2C, THF), 14.47 (2C, CH_3_). FTIR-ATR (cm^–1^): 3483 (w), 3055 (w), 2979 (w), 1665 (m), 1630 (m), 1596 (m), 1554 (m), 1513 (s), 1499 (m), 1461 (s), 1441 (vs), 1394 (s), 1370 (m), 1311 (m), 1252 (s), 1210 (s), 1153 (m), 1077 (m), 1038 (m), 966 (w), 944 (w), 871 (m), 858 (m), 814 (m), 797 (m), 752 (s), 712 (m), 656 (m), 571 (m), 536 (m), 484 (w), 471 (vw), 453 (w).

Due to the very poor solubility of compound **8** in THF-*d*_8_, the NMR spectra of its methyl derivative, [Ba­(sal-Me)_2_(THF)]*_n_* (**8a**), which exhibits significantly better solubility, were additionally recorded. ^1^H NMR (400 MHz, THF-*d*_8_): 7.72 (2H, m, ArH), 7.29 (2H, m, ArH), 6.91 (2H, m, ArH), 6.62 (2H, m, ArH), 3.75 (6H, s, OCH_3_). ^13^C NMR (101 MHz, THF-*d*_8_): δ 170.81 (2C, C=O), 165.64 (2C, C–O), 135.65 (2C, ArH), 130.96 (2C, ArH), 120.13 (2C, ArH), 117.09 (2C, ArH), 114.10 (2C, Ar), 51.88 (2C, OCH_3_).

### Synthesis of [Mn_2_(μ-OMe)_2_(sal-Me)_4_] (**9**)

To a 250 mL Schlenk flask equipped with a magnetic stir bar were introduced metallic sodium (0.1774 g; 7.72 mmol), methyl salicylate (1.00 mL; 7.72 mmol), and tetrahydrofuran (40 mL). After the complete reaction of sodium, a solution of MnCl_2_ (0.4857 g; 3.86 mmol) in methanol (5 mL) was added. The resulting mixture was stirred at room temperature for 96 h. The reaction mixture was then filtered to remove precipitated sodium chloride, concentrated under vacuum, and left undisturbed at room temperature. After 3–4 weeks of crystallization, dark brown crystals of compound **9** were collected by filtration, washed with hexane (3 × 5 mL), and dried under vacuum. Yield: 1.04 g (67%). Anal. calcd for C_34_H_34_Mn_2_O_14_: C, 52.59; H, 4.41; found: C, 52.62; H, 4.42. FTIR-ATR (cm^–1^): 3057 (vw), 3028 (vw), 3000 (vw), 2952 (w), 2931 (w), 2882 (vw), 2852 (vw), 2823 (vw), 1648 (vs), 1598 (m), 1551 (m), 1469 (m), 1443 (m), 1336 (m), 1308 (s), 1227 (vs), 1195 (m), 1158 (m), 1141 (m), 1090 (m), 1051 (m), 1035 (m), 988 (w), 970 (w), 948 (w), 863 (m), 822 (m), 798 (w), 757 (s), 704 (m), 663 (m), 626 (s), 570 (m), 543 (s), 528 (m), 480 (m), 453 (w), 430 (m), 409 (w).

### General Procedure for the Synthesis of Oxide Materials

The thermal decomposition of compounds **7** or **8** with **9** at a Ca­(Ba)/Mn molar ratio of 1:1 was employed as a general method for synthesizing alkaline-earth manganites. In a typical procedure, compounds **7** (0.491 g; 1.32 mmol) and **9** (0.515 g; 0.66 mmol), or compounds **8** (0.598 g; 1.28 mmol) and **9** (0.497 g; 0.64 mmol) were placed into 50 mL ceramic crucibles. Subsequently, methanol (10 mL), distilled water (10 mL), and diethyl glycol (5 mL) were added to each crucible. The mixtures were subjected to thermal treatment in a furnace at 850 or 1100 °C for 5 h. After cooling to room temperature, the resulting powders were washed with distilled water (50 mL) and left to dry at ambient conditions for approximately 3 days. The ceramic yield of heterometallic oxide materials obtained from the decomposition of compounds **7** and **9** was 18% at 1100 °C, while the combination of **8** and **9** yielded 27% under the same conditions. For the preparation of M^RE^(III)-doped heterometallic oxides, clusters **1**–**6** were added in an amount corresponding to 2 or 10 mol % M^RE^(III) relative to Mn­(III), and the thermal decomposition was carried out at 1100 °C.

## Supplementary Material



## Notes

X-ray crystallographic data for **1**–**9**; FTIR-ATR, ^1^H, and ^13^C NMR spectra for **1**–**9**; additional crystallographic data for **1**–**9**; PXRD and TEM-EDX measurements of obtained nanomaterials (PDF)
