# Supplementary material for: Rare-Earth Molecular Cluster Aggregates with Sandglass-like Core Topology as Surrogates for Minor Actinides in Immobilization within Alkaline-Earth Manganites
Source: Inorg Chem. 2025 Jul 25;64(31):16020–35. doi: 10.1021/acs.inorgchem.5c02206 (PMC12344766; doi:10.1021/acs.inorgchem.5c02206)
Supplement: Supplementary file 1 [file ic5c02206_si_001.pdf]

Supporting information for

Rare Earth Molecular Cluster Aggregates with Sandglass-like Core Topology as Surrogates for Minor Actinides in Immobilization within Alkaline Earth Manganites

Rafał Petrus,[1,*] Adrian Kowaliński,[1] Tadeusz Lis,[2] Miłosz Siczek,[2] and Piotr Sobota[1]

[a]Faculty of Chemistry, Wrocław University of Science and Technology, 23 Smoluchowskiego, 50-370 Wrocław, Poland

[b]Faculty of Chemistry, University of Wrocław, 14 F. Joliot-Curie, 50-383 Wrocław, Poland

*Corresponding author email: rafal.petrus@pwr.edu.pl

Contents

X-Ray Crystallography of **1-9**...S2, S18

Continuous-shape measurements of the coordination environment around metal ions in **1-9**…...S10, S21

PXRD patterns of **1-6**……………………………………………...S11

^{1}H, ^{13}C, ^{1}H-DOSY NMR spectra of **1** and **6**……………………...……S14, S19

IR spectra of **1-6**……………………………………………...S16

IR and ^{1}H, ^{13}C NMR spectra of **7-9**……………………………………..S22

TGA curves for **7-9**……………………………………………..S26

PXRD patterns of oxide materials……………………………………S27

TEM analysis of oxide materials……………………………………..S33

Crystallographic Data for Compounds 1-9.

Table S1. Crystal and data collection parameters for compounds **1** – **9**.

Crystal	**1**·EtOH	**2**·1.5(MeOH)·2.25(H_2O)	**3**·$DyCl_4$ 0.6(EtOH)·0.3(H_2O)
Chemical formula	$C_{134.69}H_{137.38}ClO_{59}Y_9$	$C_{129.50}H_{132.50}ClO_{61.75}Eu_9$	$C_{151.20}H_{172.20}Cl_4O_{58.90}Dy_{10}$
Formula Mass	3535.74	4079.94	4698.68
Crystal system	orthorhombic	monoclinic	triclinic
Space group	$Pna2_1$	$P2_1$/n	$P\bar{1}$
a/Å	32.886 (7)	21.644 (2)	17.237 (3)
b/Å	22.509 (5)	33.077 (4)	20.464 (3)
c/Å	21.989 (5)	21.965 (2)	25.466 (4)
α/°			85.02 (2)
β/°		90.14 (2)	86.04 (2)
γ/°			78.23 (2)
Unit cell volume/Å^3	16277 (6)	15725 (3)	8749 (3)
Temperature/K	100(2)	150(2)	100(2)
Z	4	4	2
Radiation type	MoKα	MoKα	MoKα
Absorption coefficient, μ/mm^{-1}	3.267	3.634	4.354
No. of reflections measured	165011	149920	126014
No. of independent reflections	38280	35998	38177
No. of observed ($I > 2\sigma(I)$) reflections	26832	28118	28935
R_{int}	0.0798	0.0339	0.0387
Final R_1 values ($I > 2\sigma(I)$)	0.0508	0.0390	0.0413
Final $wR(F^2)$ values ($I > 2\sigma(I)$)	0.1208	0.0939	0.0948
Final R_1 values (all data)	0.0918	0.0544	0.0630
Final $wR(F^2)$ values (all data)	0.1396	0.1011	0.1027
Goodness of fit on F^2	1.008	1.069	1.037
Δρmax/eÅ^{-3}	0.567	1.256	1.530
Δρmin/eÅ^{-3}	-0.354	-0.953	-2.241

Crystal	**4**·7.98(MeOH)·3.4(H_2O)	**5**·0.5(THF)·2(MeOH)	**6**·0.75(AcOMe)·0.3(MeOH)
Chemical formula	$C_{135.98}H_{160.72}ClO_{69.38}Tm_9$	$C_{132}H_{134}ClO_{60.50}Yb_9$	$C_{130.55}H_{123.70}ClO_{59.80}Lu_9$
Formula Mass	4461.02	4281.19	4259.57
Crystal system	monoclinic	monoclinic	orthorhombic
Space group	*C*2/*c*	$P2_1/n$	$Pna2_1$
a/Å	39.406 (4)	24.016 (2)	32.541 (5)
b/Å	22.065 (2)	21.272 (2)	22.641 (4)
c/Å	23.265 (3)	31.661 (3)	21.508 (2)
α/°			
β/°	121.61 (3)	108.79 (2)	
γ/°			
Unit cell volume/Å^3	17228 (6)	15313 (3)	15846 (4)
Temperature/K	100(2)	100(2)	100(2)
Z	4	4	4
Radiation type	MoKα	MoKα	MoKα
Absorption coefficient, μ/mm^{-1}	4.683	5.542	5.649
No. of reflections measured	205099	354519	150571
No. of independent reflections	16949	28459	34501
No. of observed ($I > 2\sigma(I)$) reflections	13909	24280	31811
R_{int}	0.0333	0.0767	0.0346
Final R_1 values ($I > 2\sigma(I)$)	0.0451	0.0585	0.0338
Final $wR(F^2)$ values ($I > 2\sigma(I)$)	0.1025	0.1219	0.0891
Final R_1 values (all data)	0.0631	0.0692	0.0406
Final $wR(F^2)$ values (all data)	0.1262	0.1281	0.0949
Goodness of fit on F^2	1.285	1.130	1.084
Δρmax/eÅ^{-3}	0.968	2.847	1.233
Δρmin/eÅ^{-3}	-0.871	-1.553	-0.914

Crystal	**7**	**7a**·THF	**8**	**9**
Chemical formula	$C_{18}H_{18}CaO_6$	$C_{66}H_{78}Ca_3O_{21}$	$C_{21.10}H_{24.20}BaO_7$	$C_{34}H_{34}Mn_2O_{14}$
Formula Mass	370.40	1327.52	527.14	776.49
Crystal system	monoclinic	triclinic	orthorhombic	monoclinic
Space group	$P2_1/c$	$P\bar{1}$	*Pbca*	$P\ 2_1/c$
a/Å	11.132 (3)	11.980 (2)	19.596 (11)	8.934 (2)
b/Å	23.454 (6)	12.377 (2)	7.928 (2)	8.995 (2)
c/Å	6.8209 (19)	12.869 (2)	27.851 (17)	20.592 (6)
α/°		69.08 (5)		
β/°	94.32 (5)	66.51 (5)		91.09 (4)
γ/°		73.30 (5)		
Unit cell volume/Å^3	1775.8 (8)	1611.1 (10)	4327.0 (4)	1654.5 (7)
Temperature/K	100(2)	100(2)	100(2)	100
Z	4	1	8	2
Radiation type	Mo$K\alpha$	Mo$K\alpha$	Mo$K\alpha$	Cu$K\alpha$
Absorption coefficient, μ/mm^{-1}	0.384	0.333	1.874	6.834
No. of reflections measured	6423	24216	14757	11027
No. of independent reflections	3563	7025	4705	3004
No. of observed ($I > 2\sigma(I)$) reflections	2074	6026	2481	2455
R_{int}	0.0672	0.0256	0.1177	0.0556
Final R_1 values ($I > 2\sigma(I)$)	0.0657	0.0397	0.0668	0.0590
Final $wR(F^2)$ values ($I > 2\sigma(I)$)	0.0966	0.0976	0.0890	0.1529
Final R_1 values (all data)	0.1318	0.0484	0.1559	0.0730
Final $wR(F^2)$ values (all data)	0.1193	0.1028	0.1112	0.1631
Goodness of fit on F^2	0.980	1.017	1.039	1.045
$\Delta\rho$max/eÅ^{-3}	0.565	0.617	0.861	1.113
$\Delta\rho$min/eÅ^{-3}	-0.489	-0.380	-0.779	-0.548

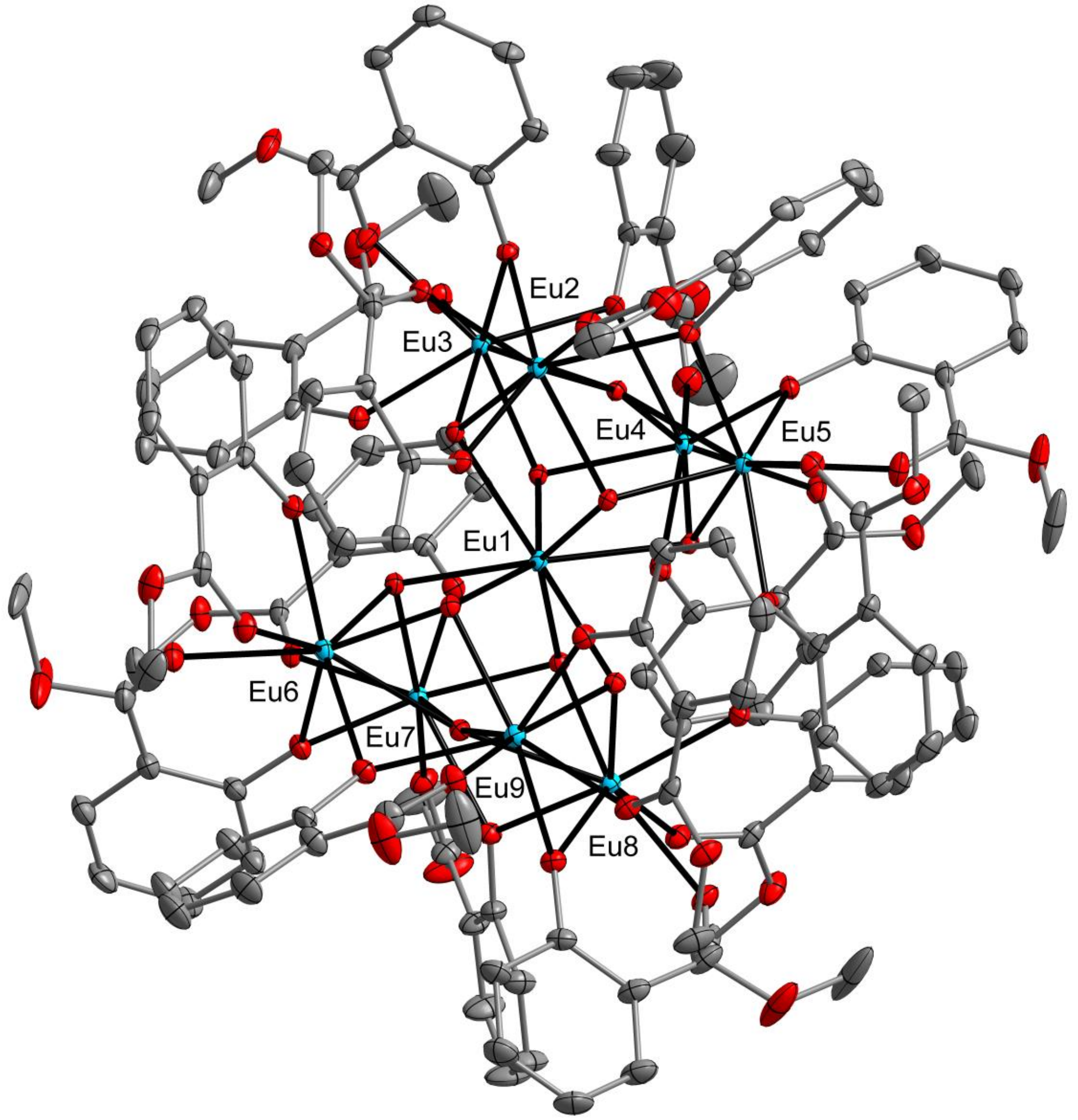

Figure S1. The molecular structure of $[Eu_9(\mu_4\text{-}OH)_2(\mu_3\text{-}OH)_8(\text{sal-Me})_{16}]Cl$ (**2**). The displacement ellipsoids are drawn at the 20% probability level. Hydrogen atoms, solvent molecules, Cl^- anion, and disorder counterparts of solvents or Cl^- are omitted for clarity.

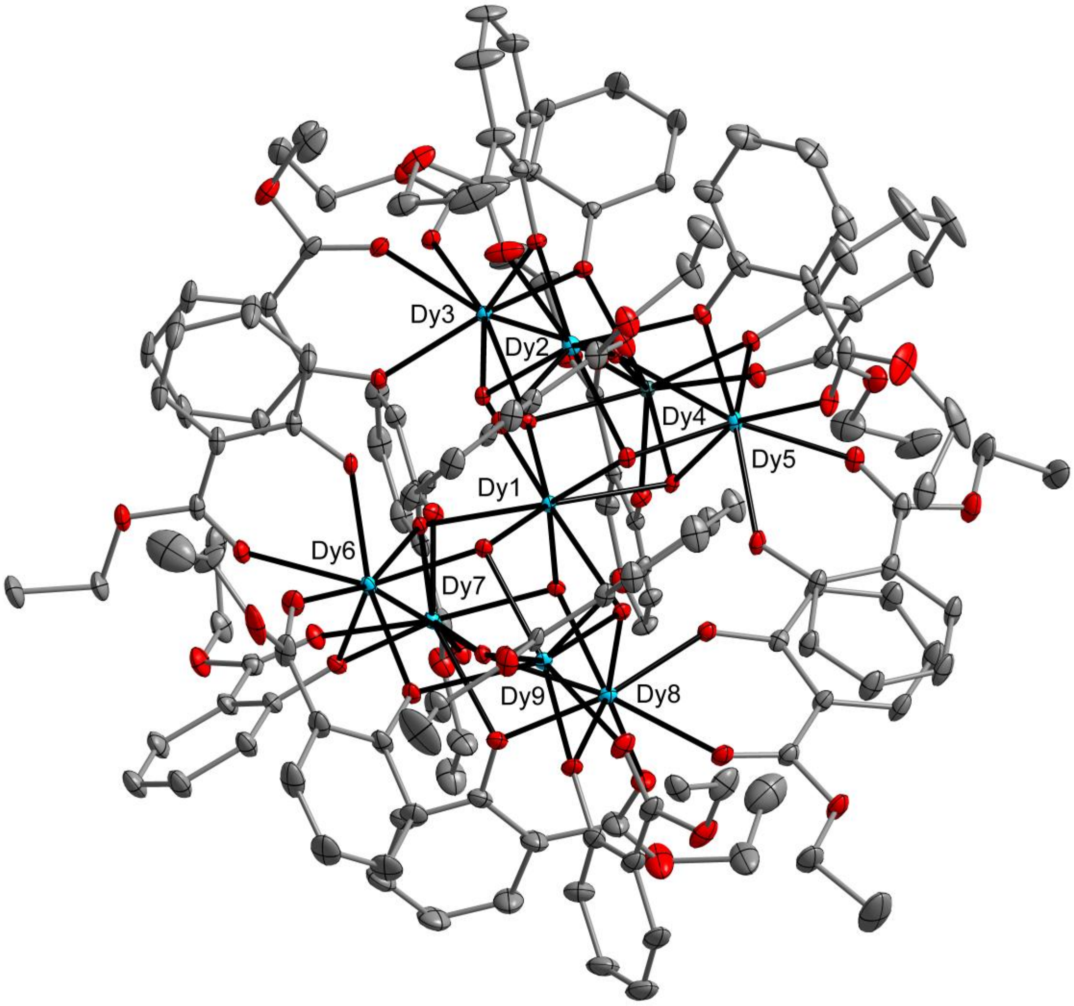

Figure S2. The molecular structure of [$Dy_9(\mu_4\text{-}OH)_2(\mu_3\text{-}OH)_8$(sal-Et)$_{16}$][$DyCl_4$] (**3**). The displacement ellipsoids are drawn at the 20% probability level. Hydrogen atoms, solvents molecules, and [$DyCl_4$]$^-$ anion are omitted for clarity.

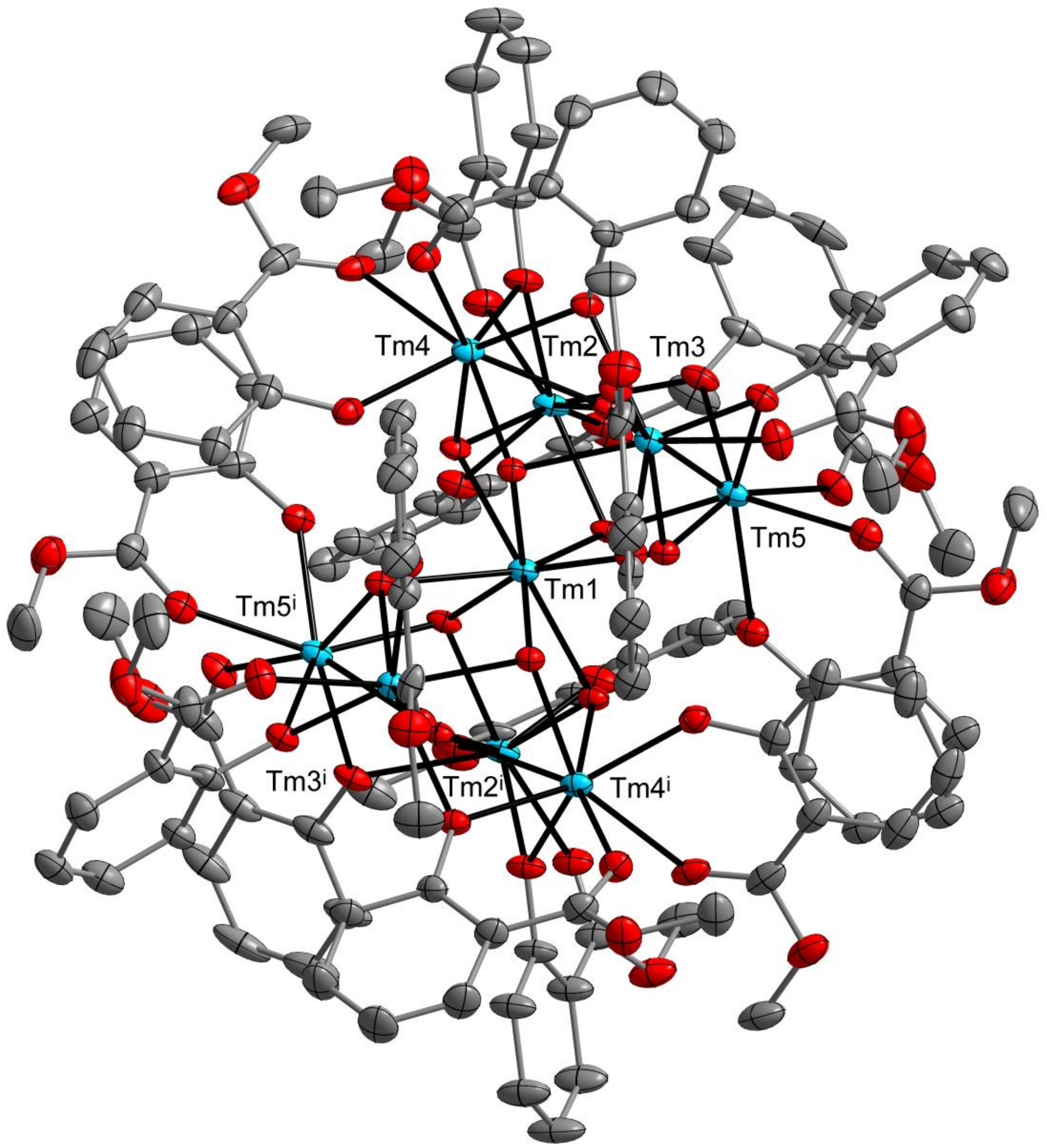

Figure S3. The molecular structure of $[Tm_9(\mu_4\text{-}OH)_2(\mu_3\text{-}OH)_8(\text{sal-Me})_{16}]Cl$ (**4**). The displacement ellipsoids are drawn at the 20% probability level. Hydrogen atoms, solvents molecules, Cl^- anion and disorder counterparts of solvents or Cl^- are omitted for clarity.

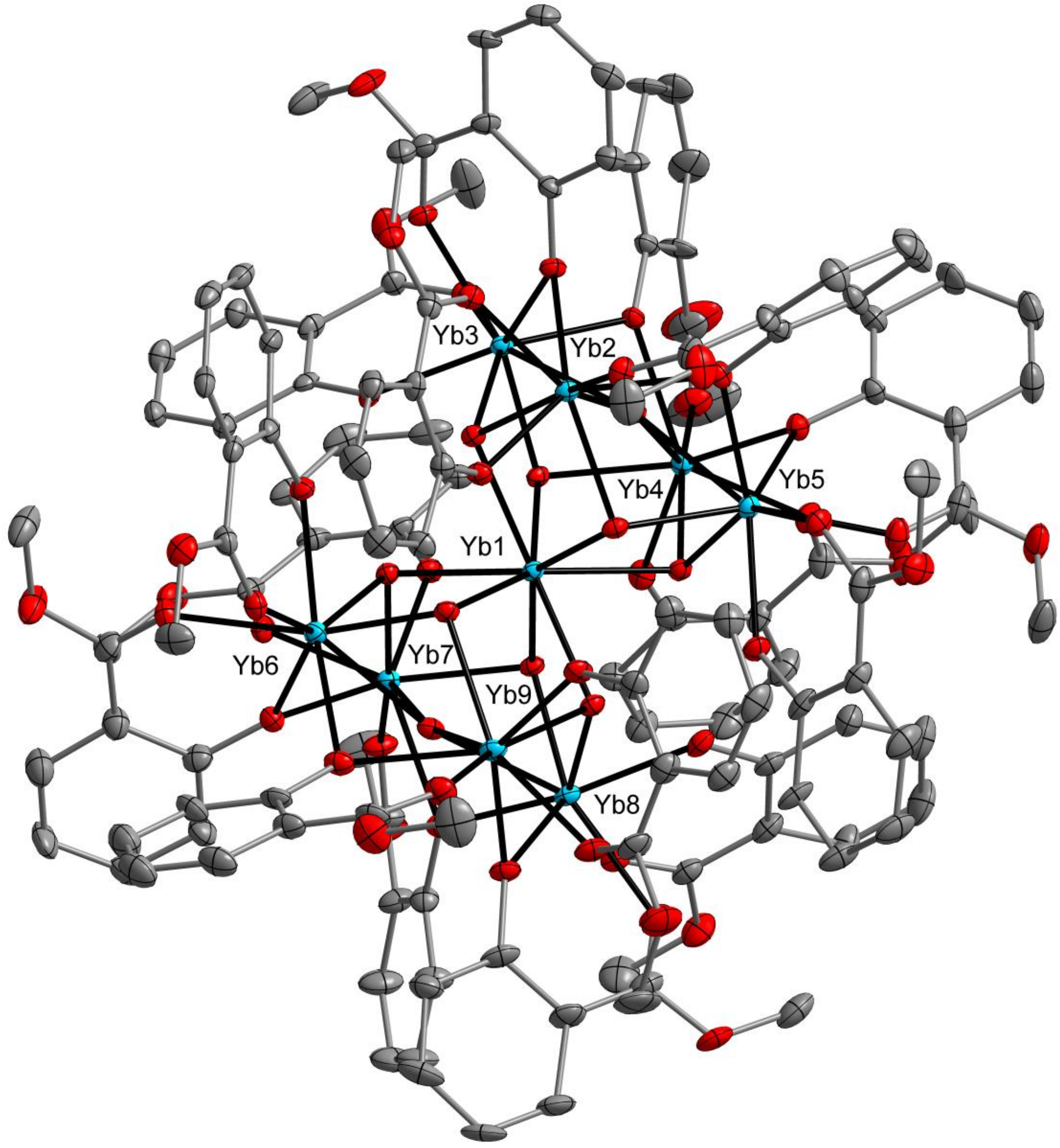

Figure S4. The molecular structure of $[Yb_9(\mu_4\text{-}OH)_2(\mu_3\text{-}OH)_8(\text{sal-Me})_{16}]Cl$ (**5**). The displacement ellipsoids are drawn at the 20% probability level. Hydrogen atoms, solvents molecules, Cl^- anion and disorder counterparts of solvents or Cl^- are omitted for clarity.

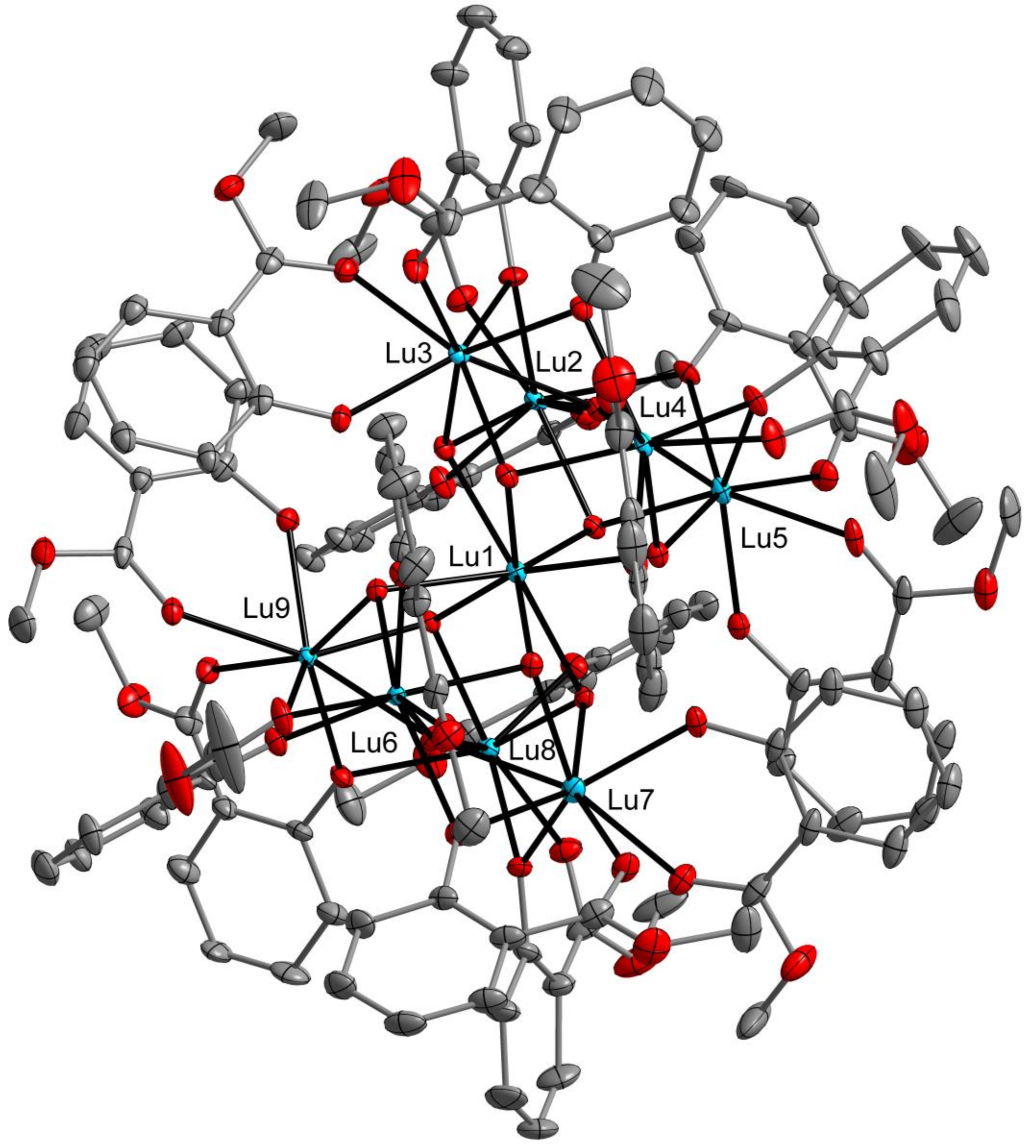

Figure S5. The molecular structure of $[Lu_9(\mu_4\text{-OH})_2(\mu_3\text{-OH})_8(\text{sal-Me})_{16}]Cl$ (**6**). The displacement ellipsoids are drawn at the 20% probability level. Hydrogen atoms, solvents molecules, Cl^- anion and disorder counterparts of solvents or Cl^- are omitted for clarity.

Table S2. Continuous-shape measurements (CShM) of the coordination environment around metal ions in **1-6**.

		***S* parameter**								
atom		RE1	RE2	RE3	RE4	RE5	RE6	RE7	RE8	RE9
compound	**1** (RE = Y)	0.056	2.375	1.919	2.124	2.411	2.031	1.842	1.947	1.967
	**2** (RE = Eu)	0.047	1.774	1.814	2.126	2.202	2.275	2.421	2.122	2.369
	**3** (RE = Dy)	0.038	2.115	1.776	2.297	2.117	1.923	2.617	2.411	1.735
	**4** (RE = Tm)	0.034	2.295	1.792	1.934	2.060	2.296	1.790	1.935	2.060
	**5** (RE = Yb)	0.042	1.497	2.475	1.909	1.964	2.364	2.052	1.699	2.149
	**6** (RE = Lu)	0.067	1.960	1.805	2.189	2.470	1.874	1.721	1.870	2.035
donor atoms		O_8								
polyhedron		square antiprism	biaugmented trigonal prism							

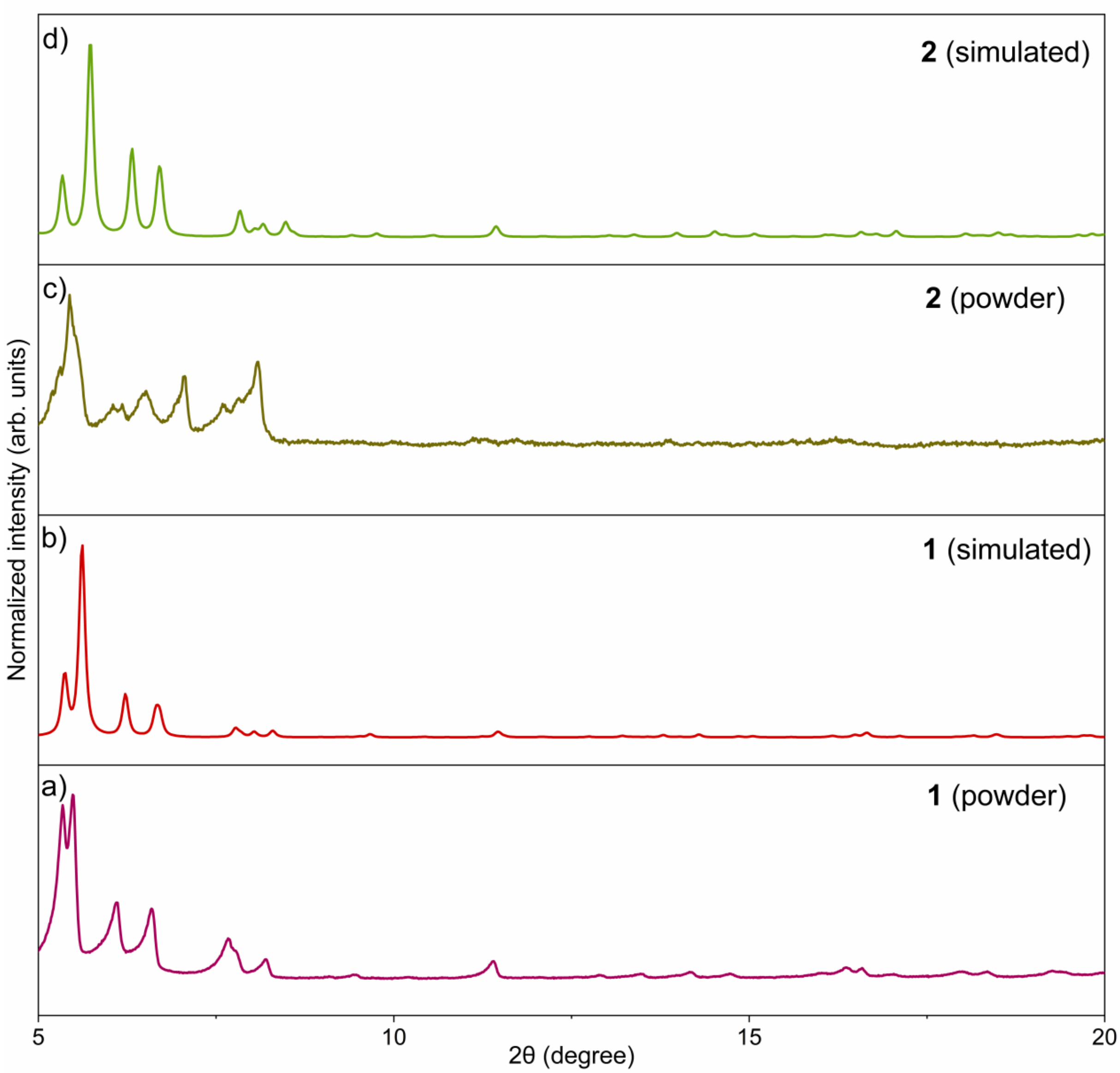

Figure S6. Comparison of PXRD patterns of crystalline powders of compounds **1** (a) and **2** (c) with their corresponding reference patterns simulated from single-crystal X-ray data (b and d).

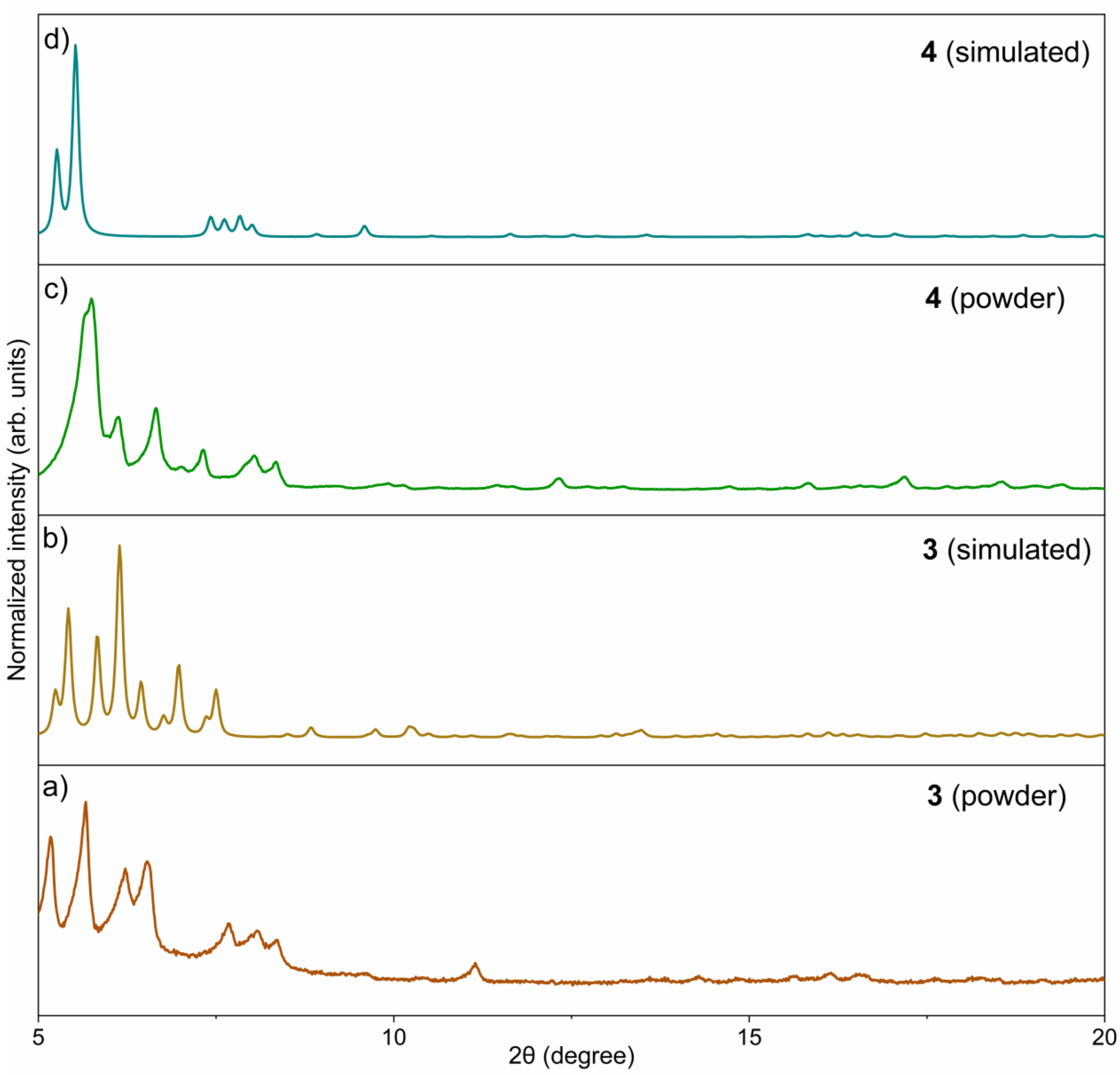

Figure S7. Comparison of PXRD patterns of crystalline powders of compounds **3** (a) and **4** (c) with their corresponding reference patterns simulated from single-crystal X-ray data (b and d).

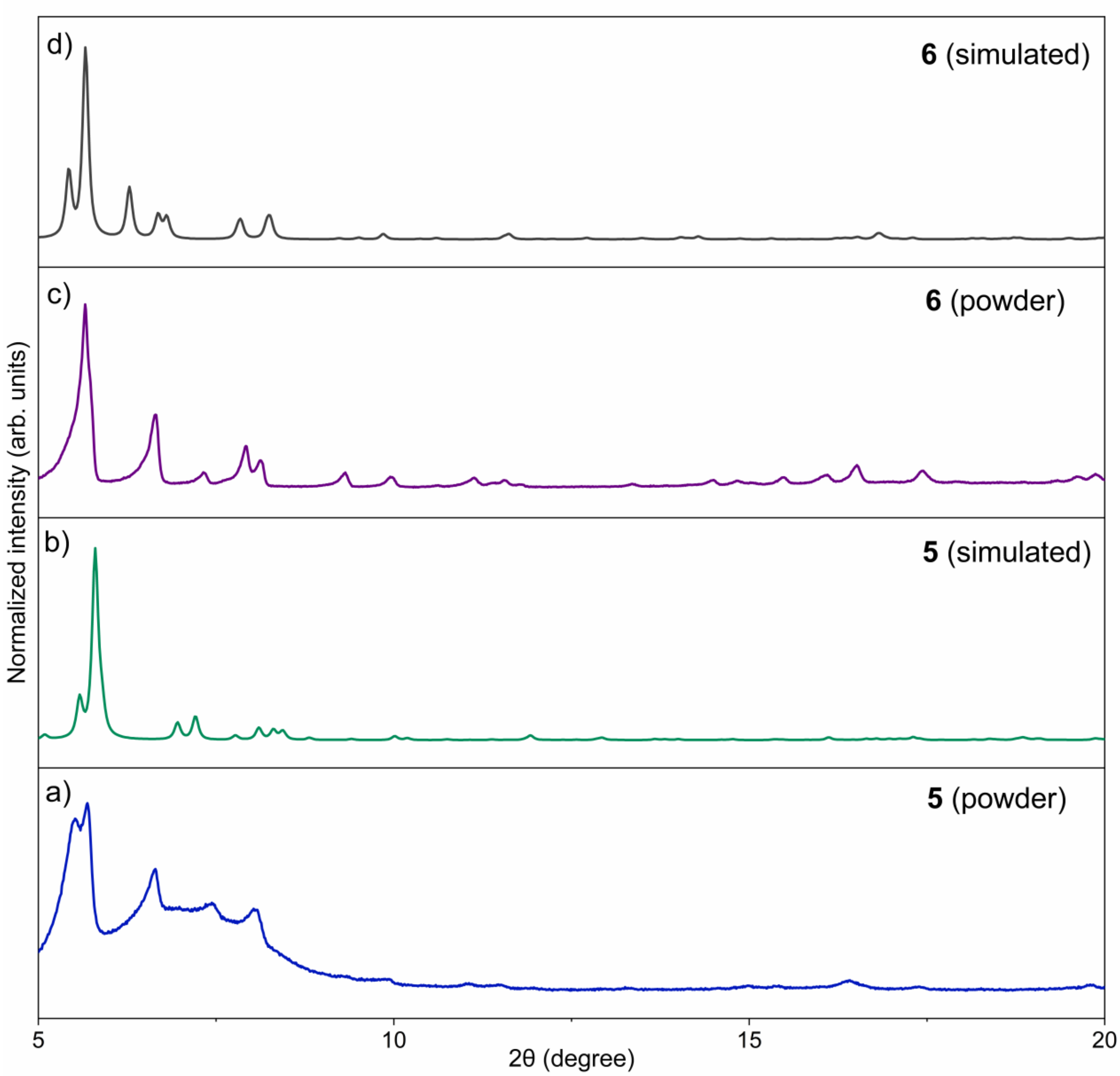

Figure S8. Comparison of PXRD patterns of crystalline powders of compounds **5** (a) and **6** (c) with their corresponding reference patterns simulated from single-crystal X-ray data (b and d).

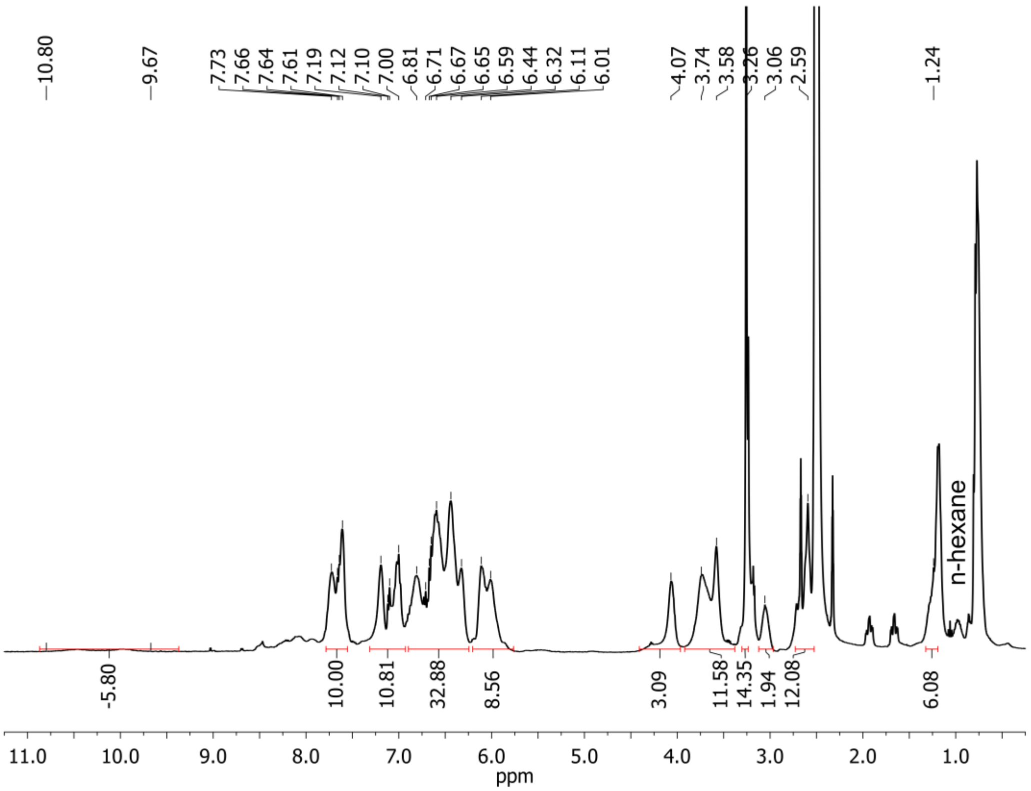

Figure S9. ^{1}H NMR spectrum of **1** in DMSO-d6.

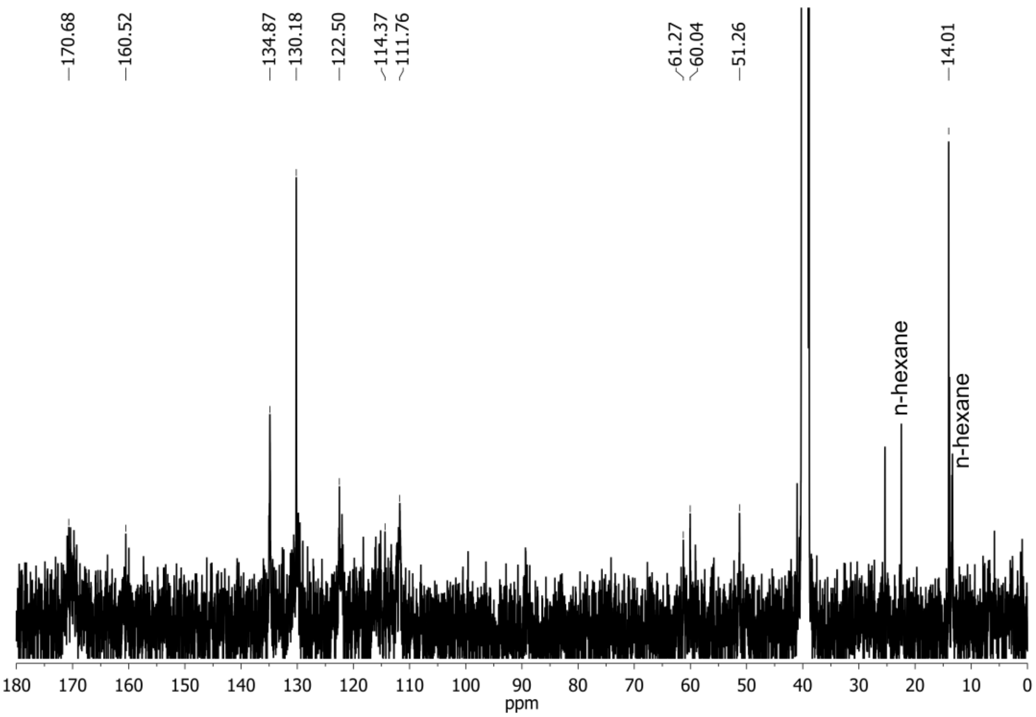

Figure S10. ^{13}C NMR spectrum of **1** in DMSO-d6.

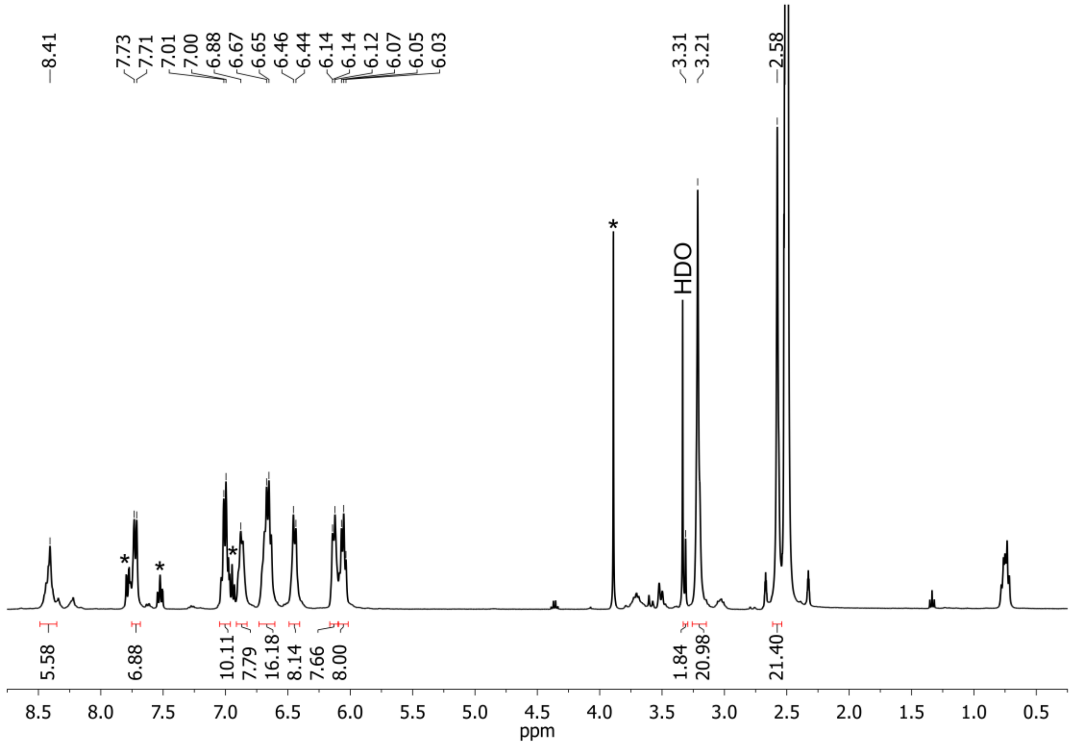

Figure S11. ^{1}H NMR spectrum of **6** in DMSO-d_6. * denotes trace amounts of free Hsal-Me.

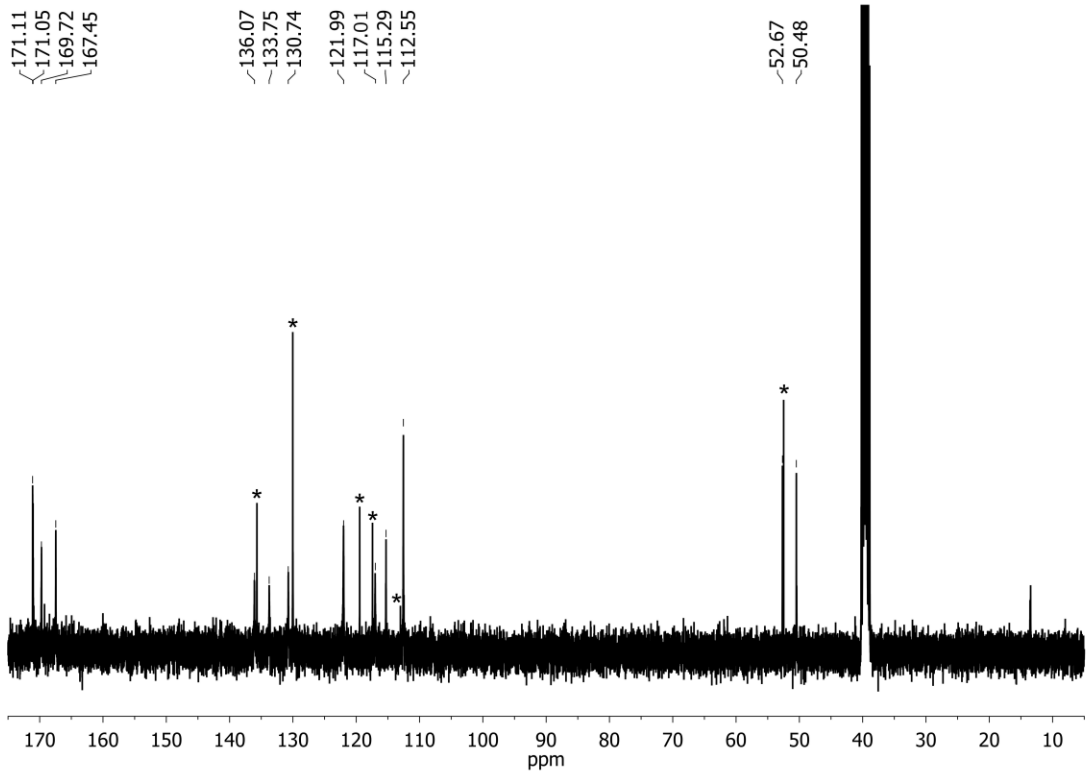

Figure S12. ^{13}C NMR spectrum of **6** in DMSO-d_6. * denotes trace amounts of free Hsal-Me.

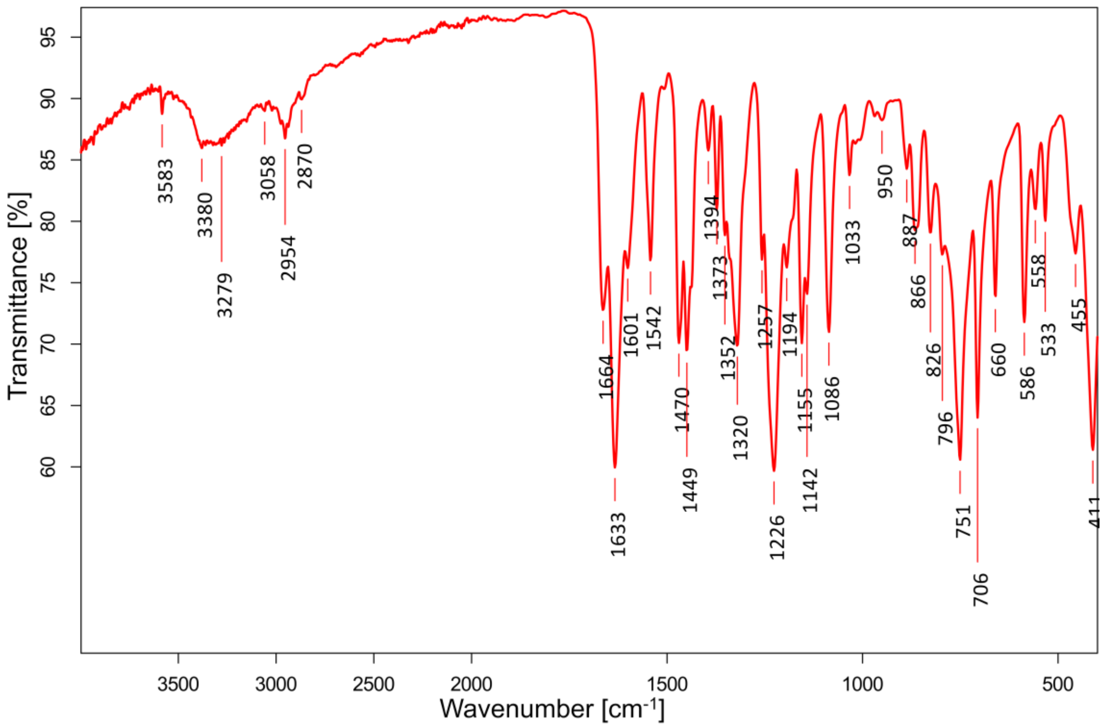

Figure S13. FTIR-ATR spectrum of **1**.

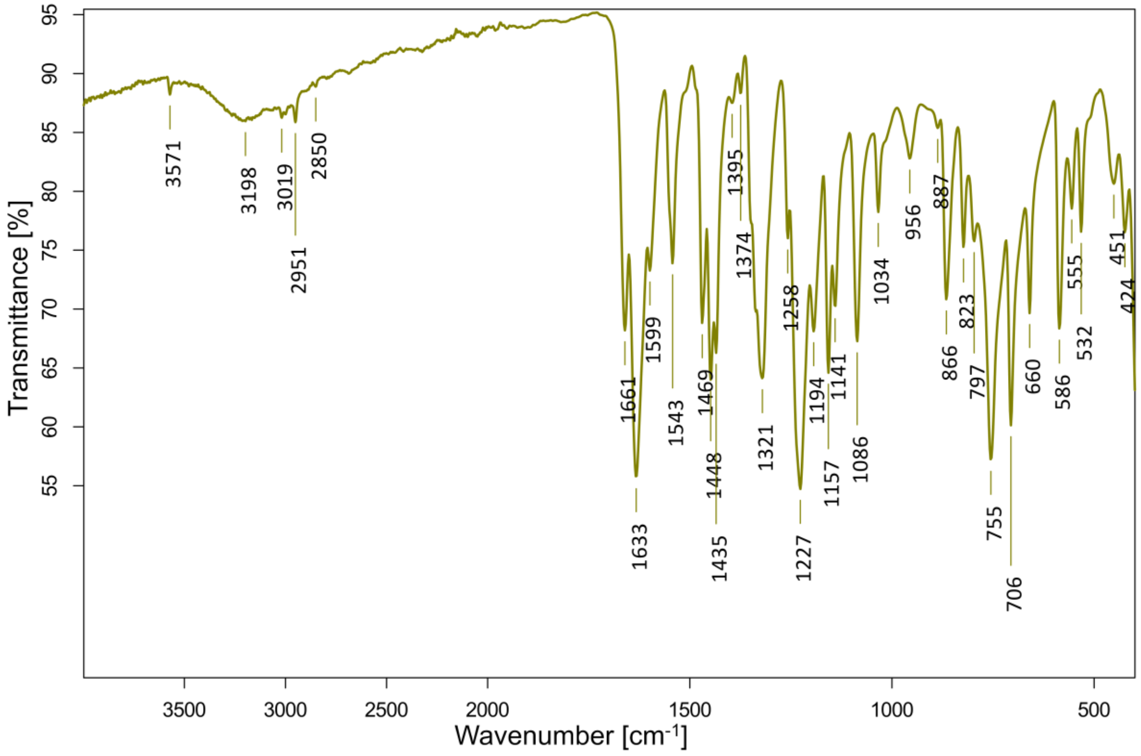

Figure S14. FTIR-ATR spectrum of **2**.

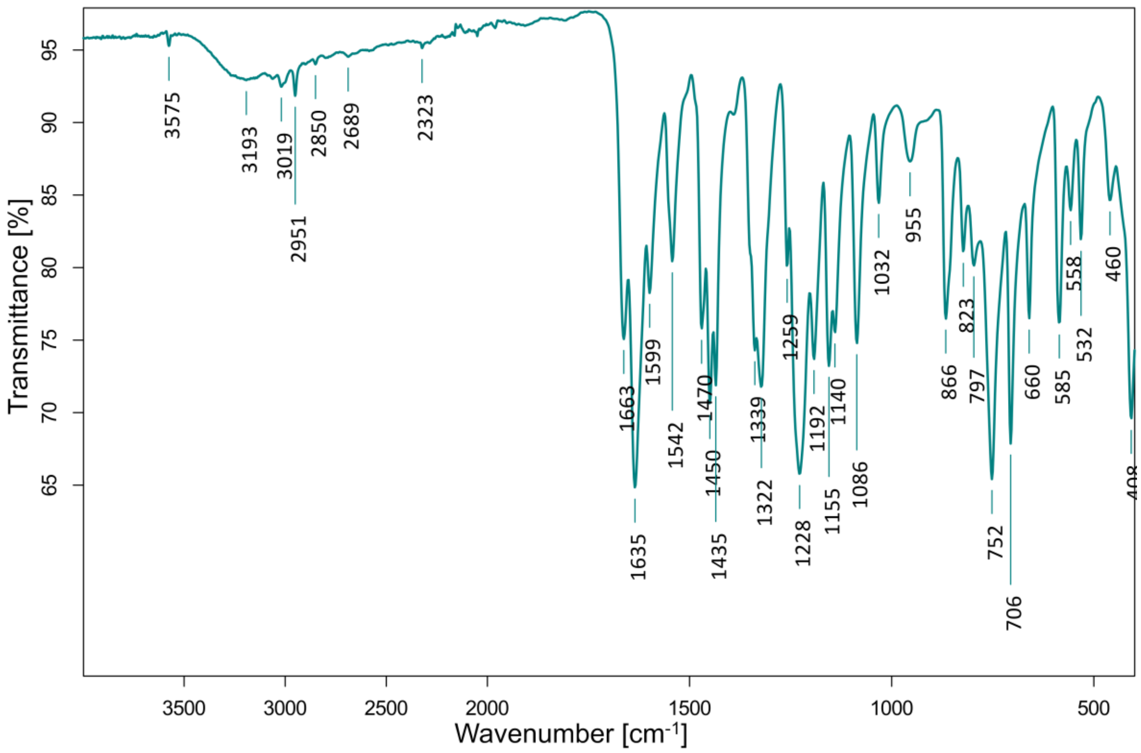

Figure S15. FTIR-ATR spectrum of **3**.

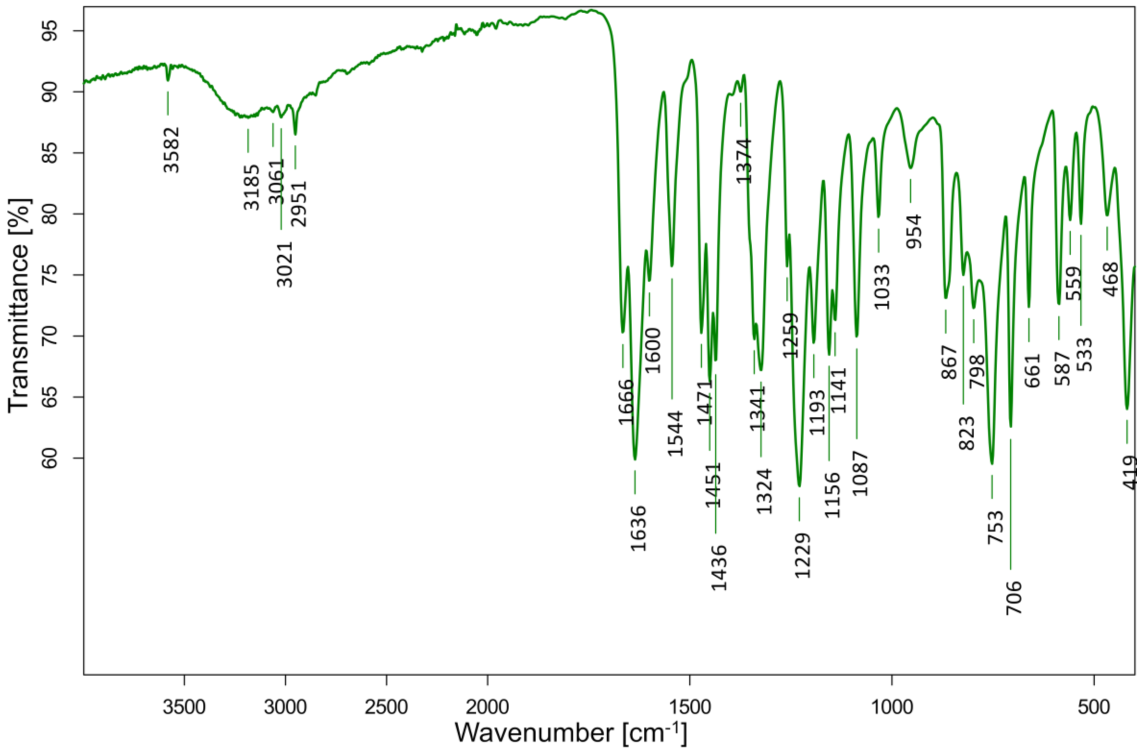

Figure S16. FTIR-ATR spectrum of **4**.

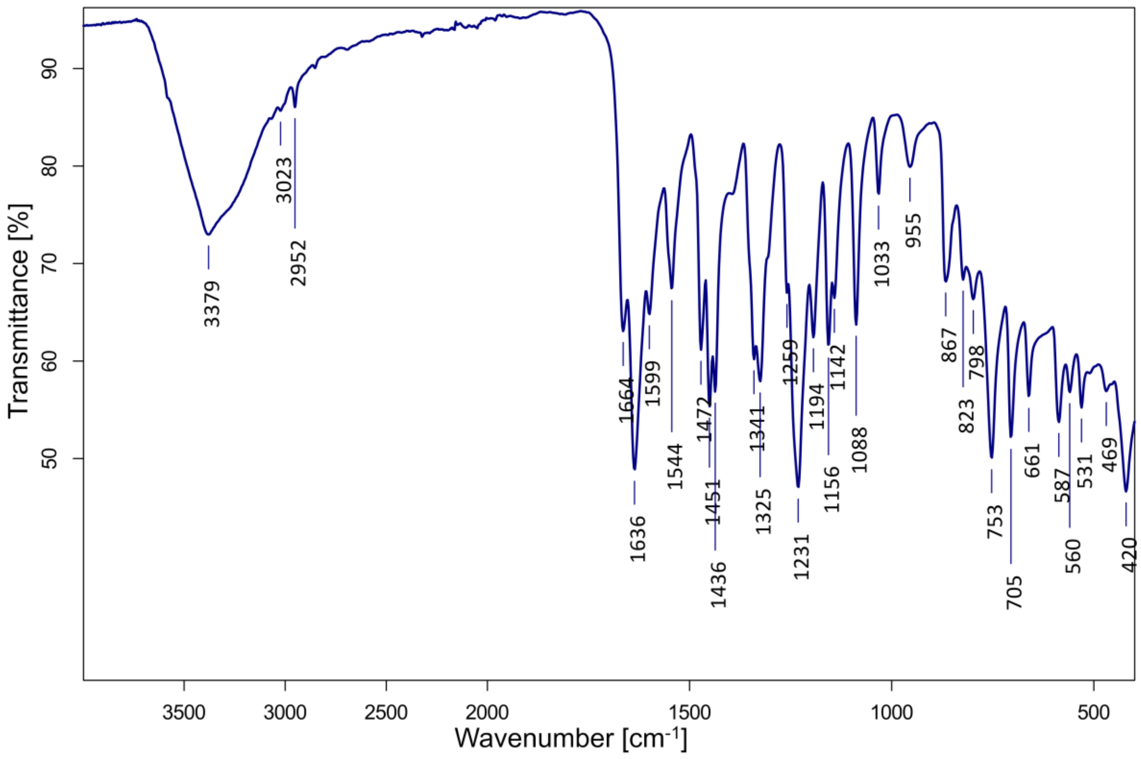

Figure S17. FTIR-ATR spectrum of **5**.

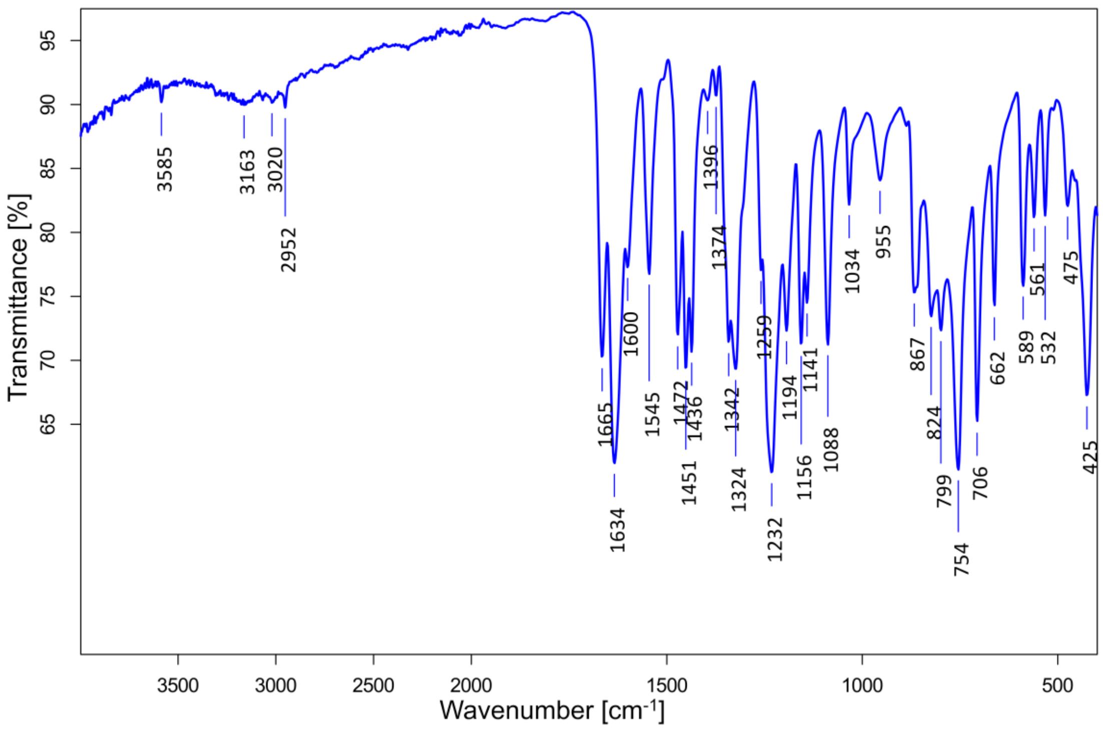

Figure S18. FTIR-ATR spectrum of **6**.

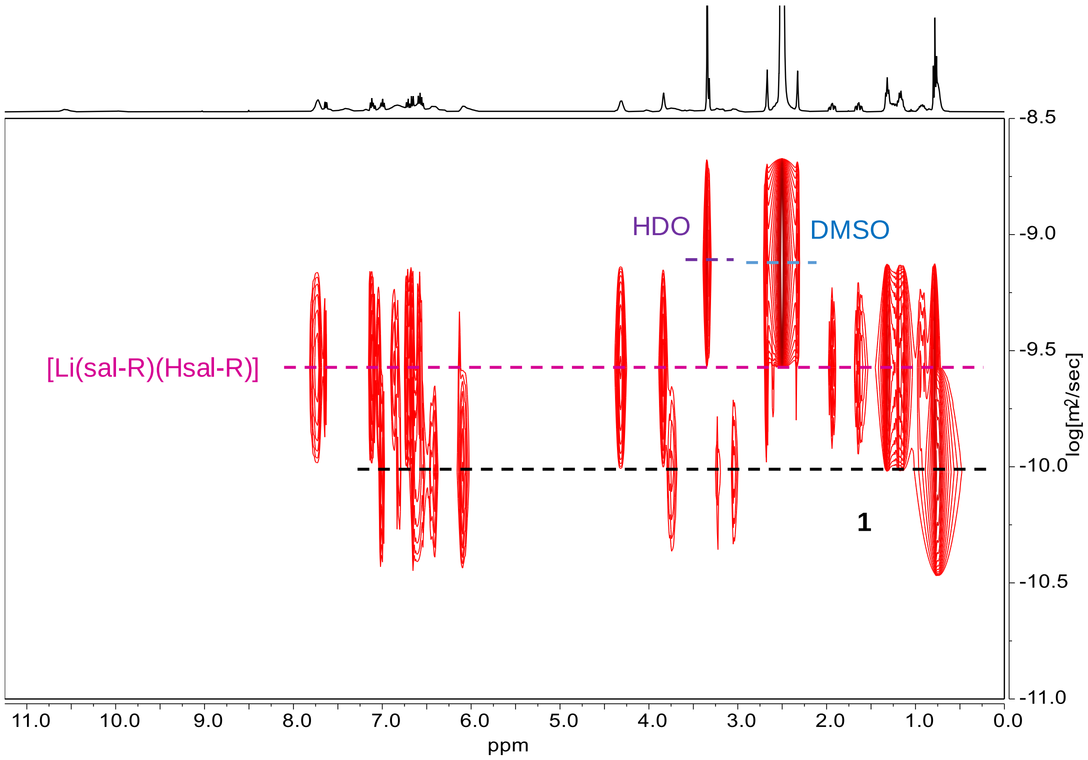

Figure S19. ^{1}H-DOSY NMR spectrum of **1** in DMSO-d_6 (T = 20.4 °C, $\log D_{x(norm)}$ = -10.078 log(m^2/sec), FW = 3513 g/mol, rH = 13.05 Å).

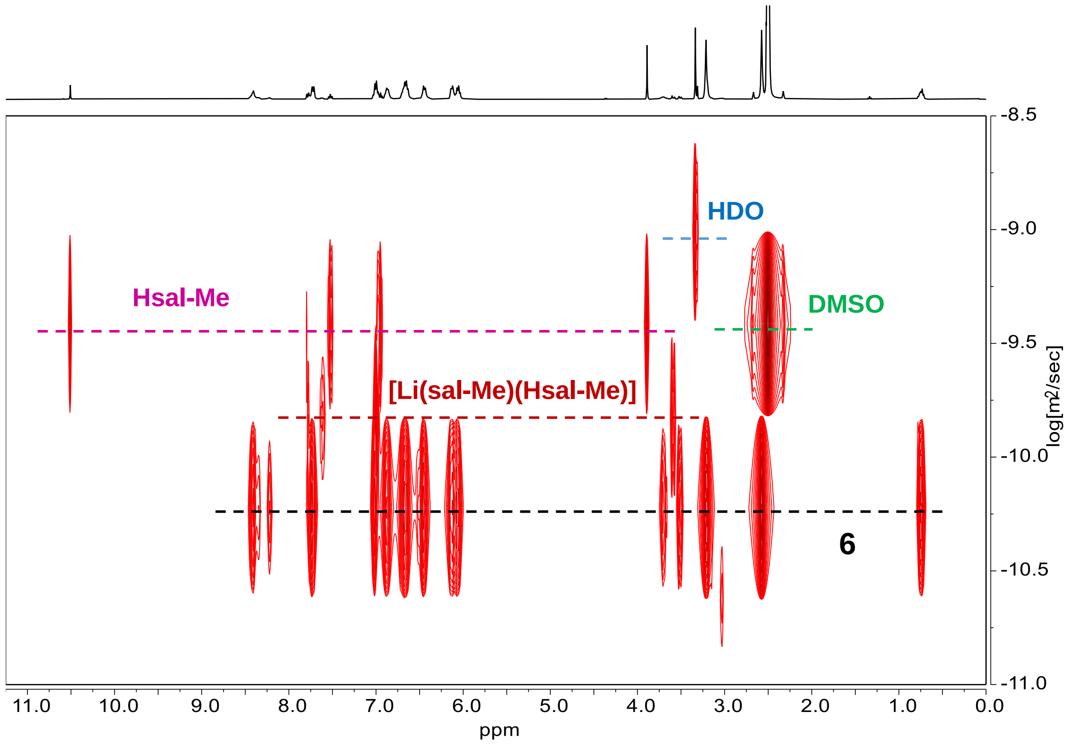

Figure S20. ^{1}H-DOSY NMR spectrum of **6** in DMSO-d_6 (T = 20.4 °C, $\log D_{x(norm)}$ = -10.112 log(m^2/sec), FW = 4250 g/mol, rH = 13.9 Å).

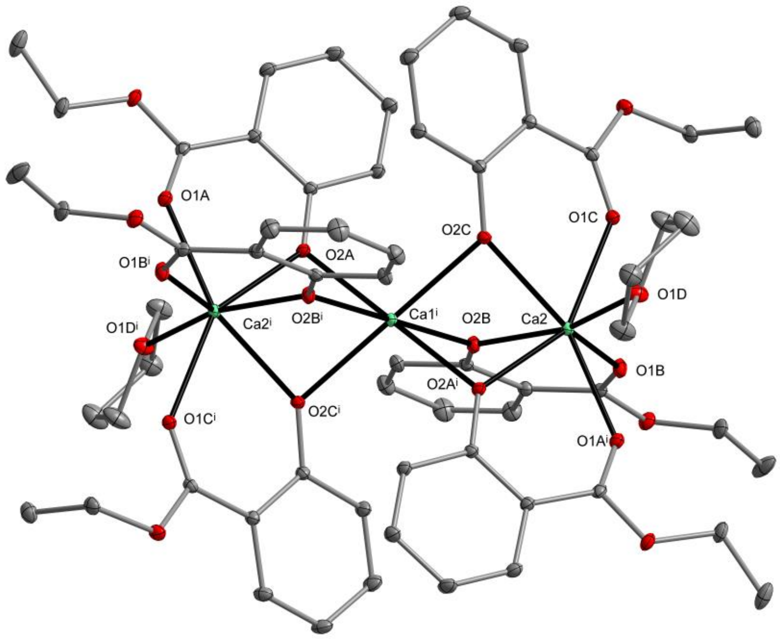

Figure S21. Molecular structure of [Ca_3(sal-Et)$_6$(THF)$_2$] (**7a**). Displacement ellipsoids are drawn at the 20% probability level. Hydrogen atoms and solvent molecules are omitted for clarity [symmetry code: (i) 1-x, 1-y, 1-z].

Table S3. Continuous-shape measurements (CShM) of the coordination environment around metal ions in **7-9**.

compound	atom	donor atoms	polyhedron	***S* parameter**
**7**	Ca1	O_6	trigonal prism	4.826
**7a**	Ca1	O_6	octahedron	2.392
	Ca2	O_7	capped trigonal prism	1.104
**8**	Ba1	O_8	biagumented trigonal prism	3.594
**9**	Mn1	O_6	octahedron	1.543

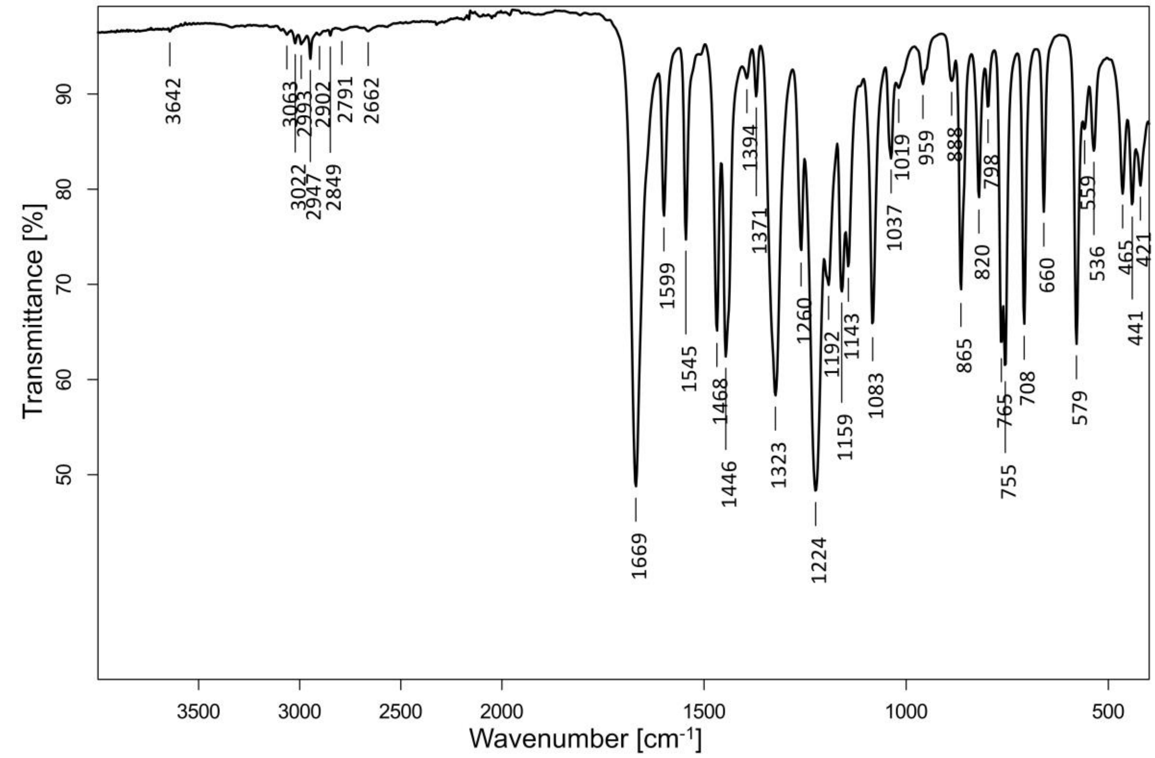

Figure S22. FTIR-ATR spectrum of **7**.

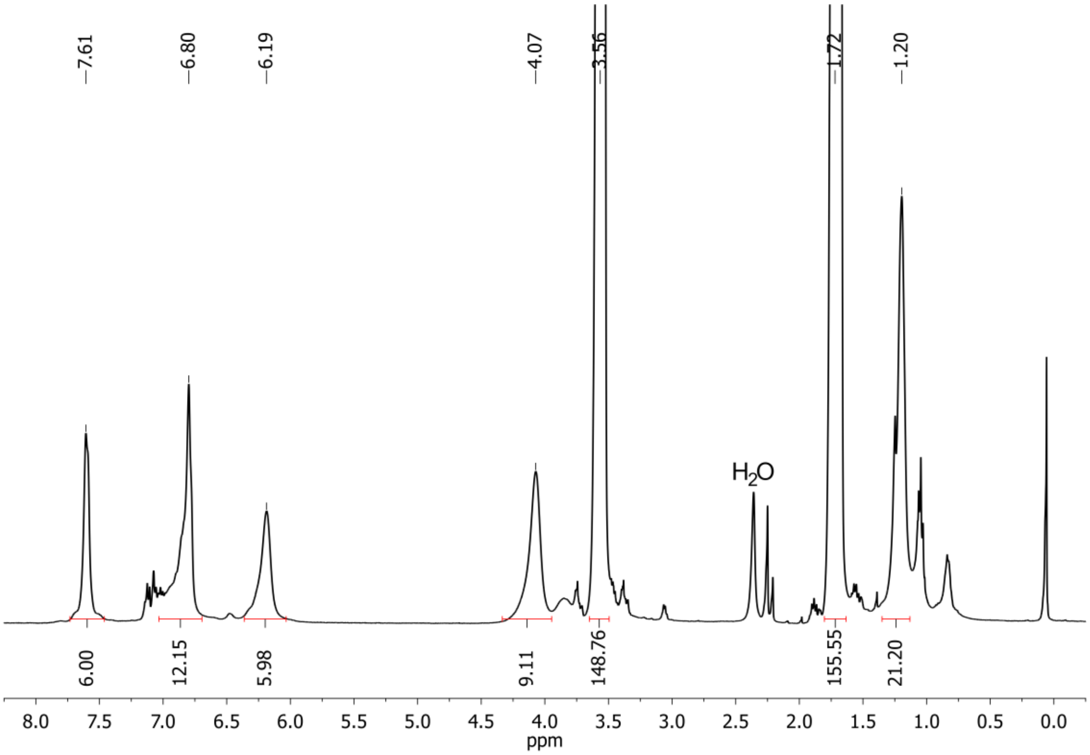

Figure S23. ^{1}H NMR spectrum of **7a** in THF-d_8.

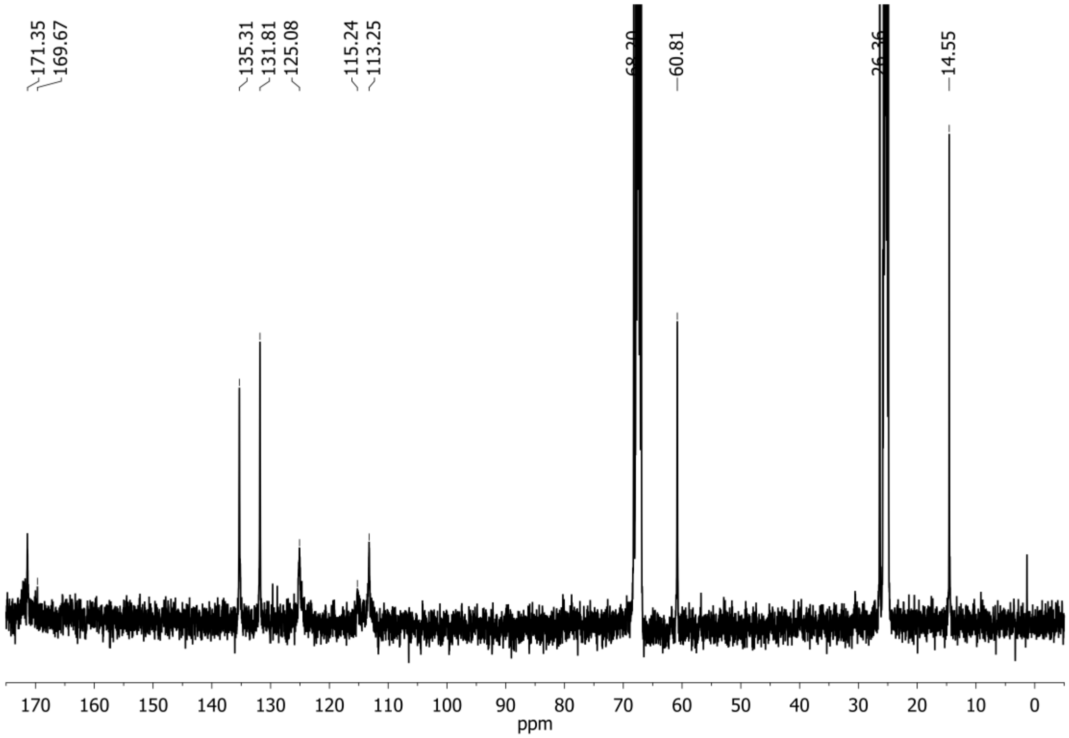

Figure S24. ^{13}C NMR spectrum of **7a** in THF-d_8.

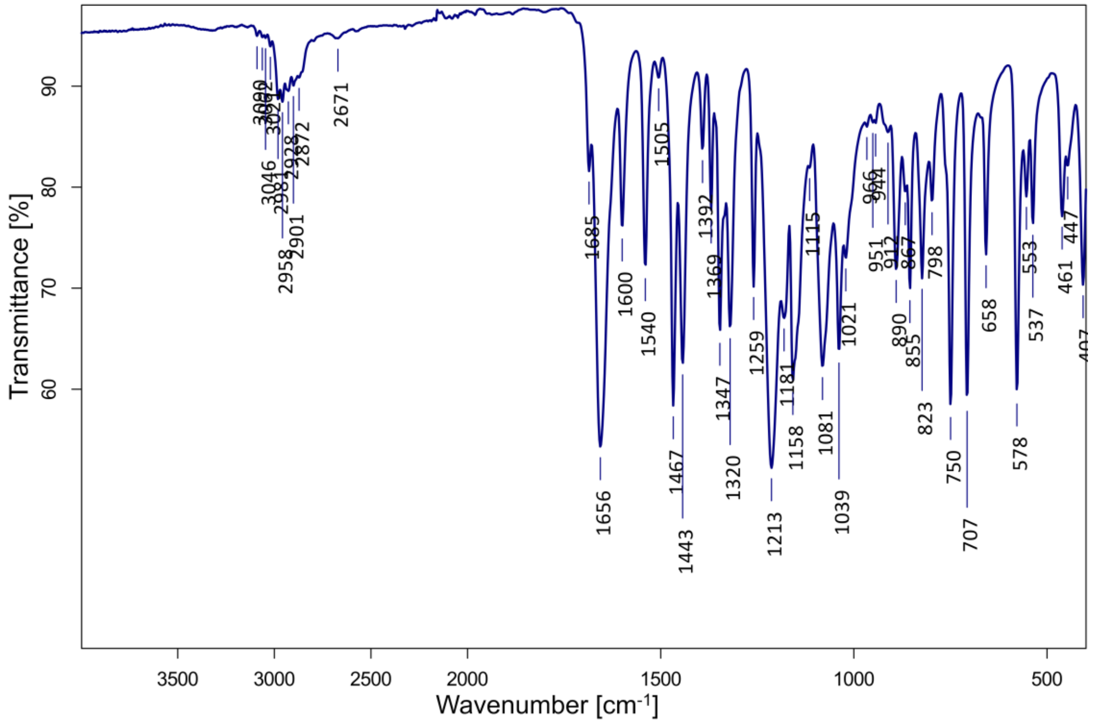

Figure S25. FTIR-ATR spectrum of **7a**.

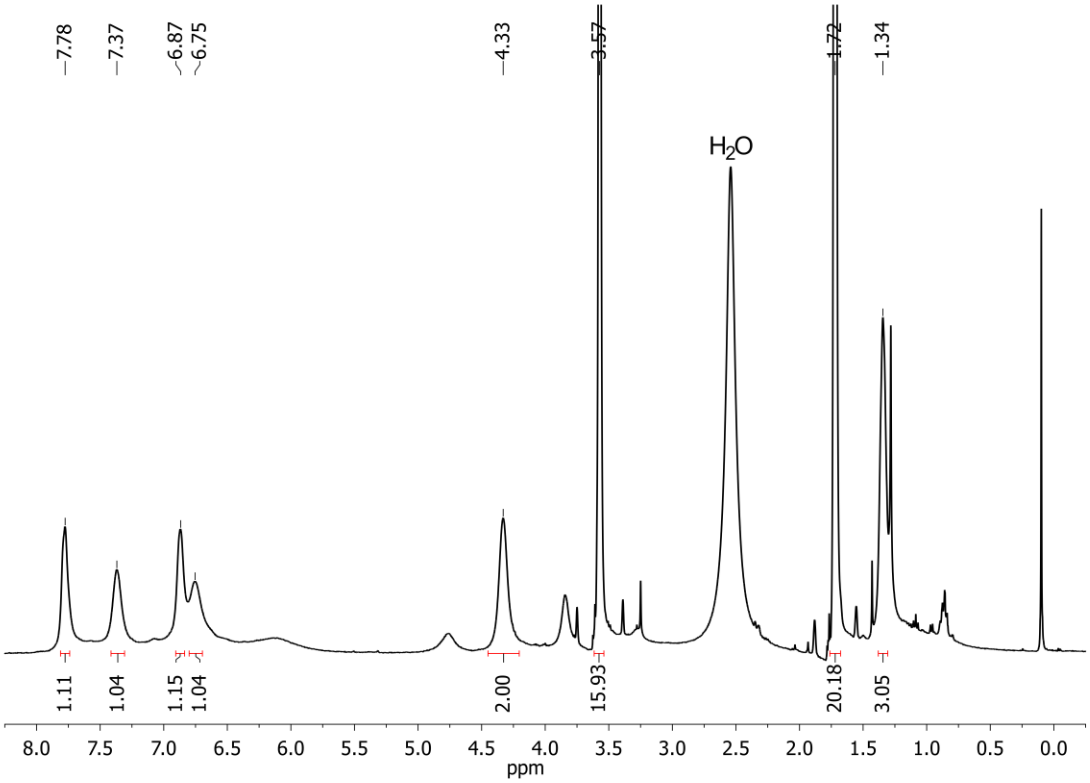

Figure S26. ^{1}H NMR spectrum of **8** in THF-d_8.

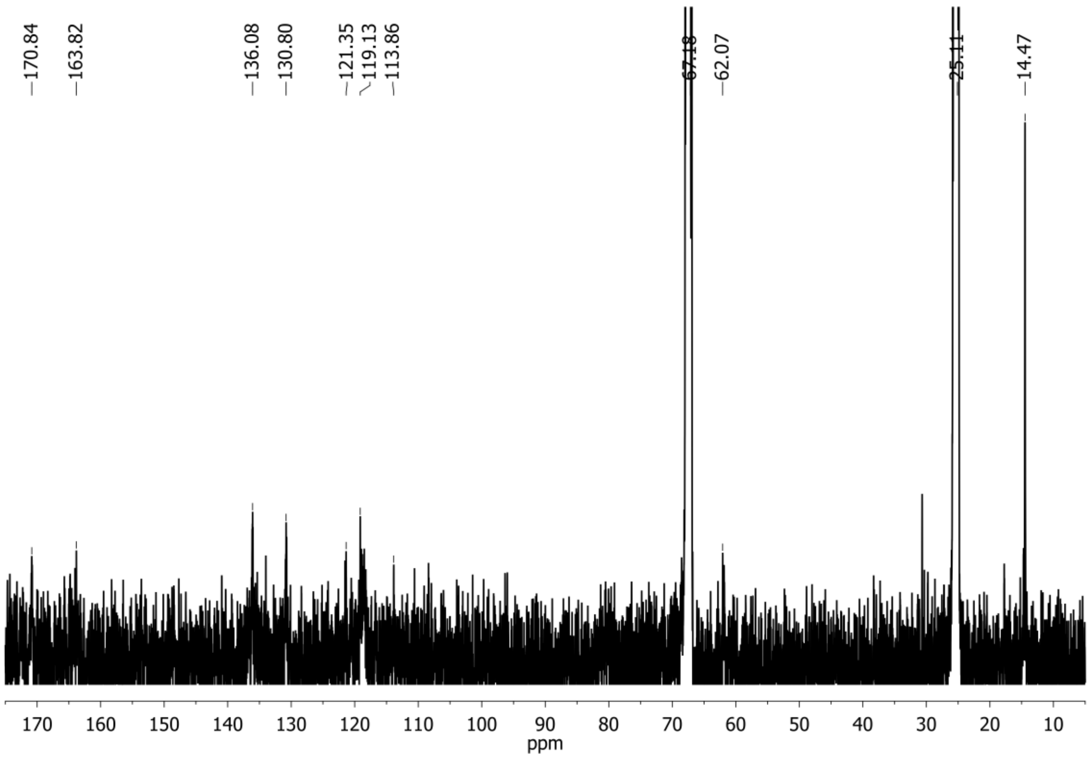

Figure S27. ^{13}C NMR spectrum of **8** in THF-d_8.

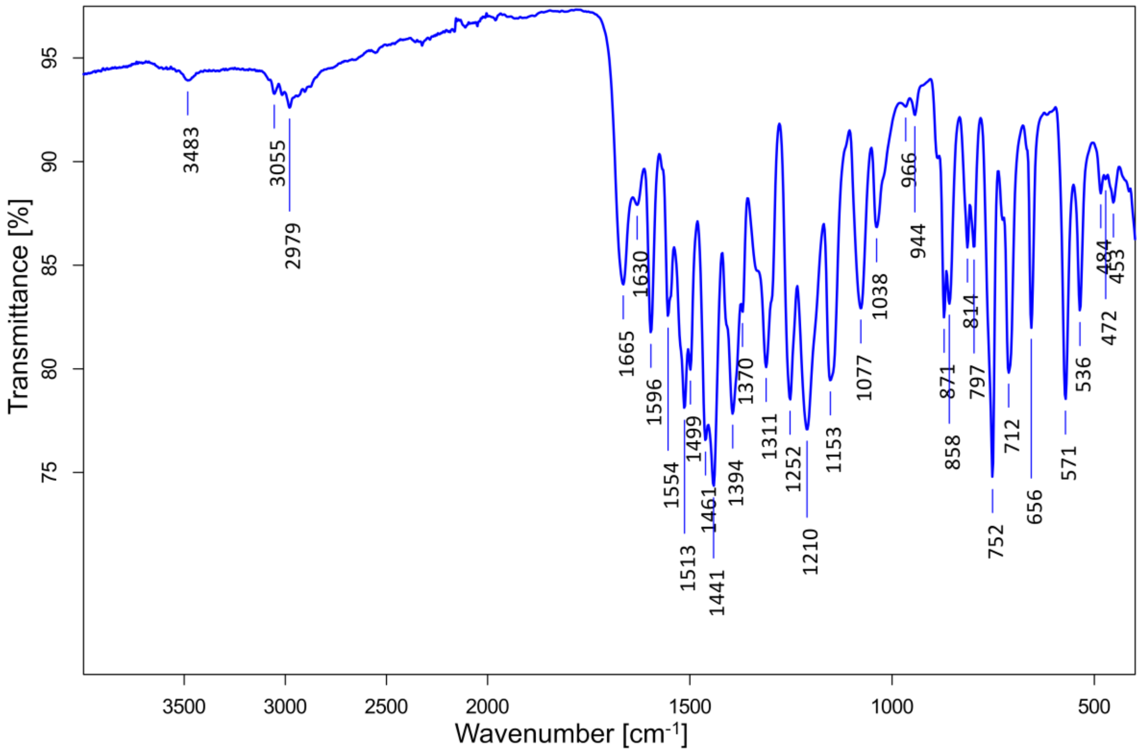

Figure S28. FTIR-ATR spectrum of **8**.

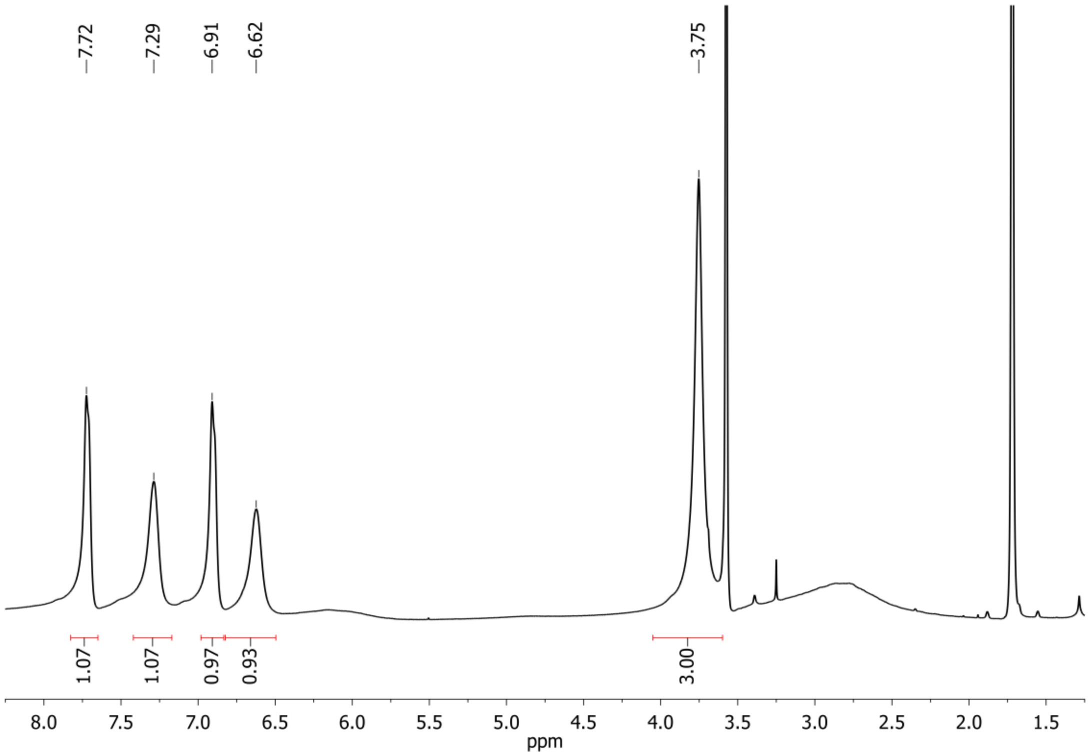

Figure S29. ^{1}H NMR spectrum of **8a** in THF-d_8.

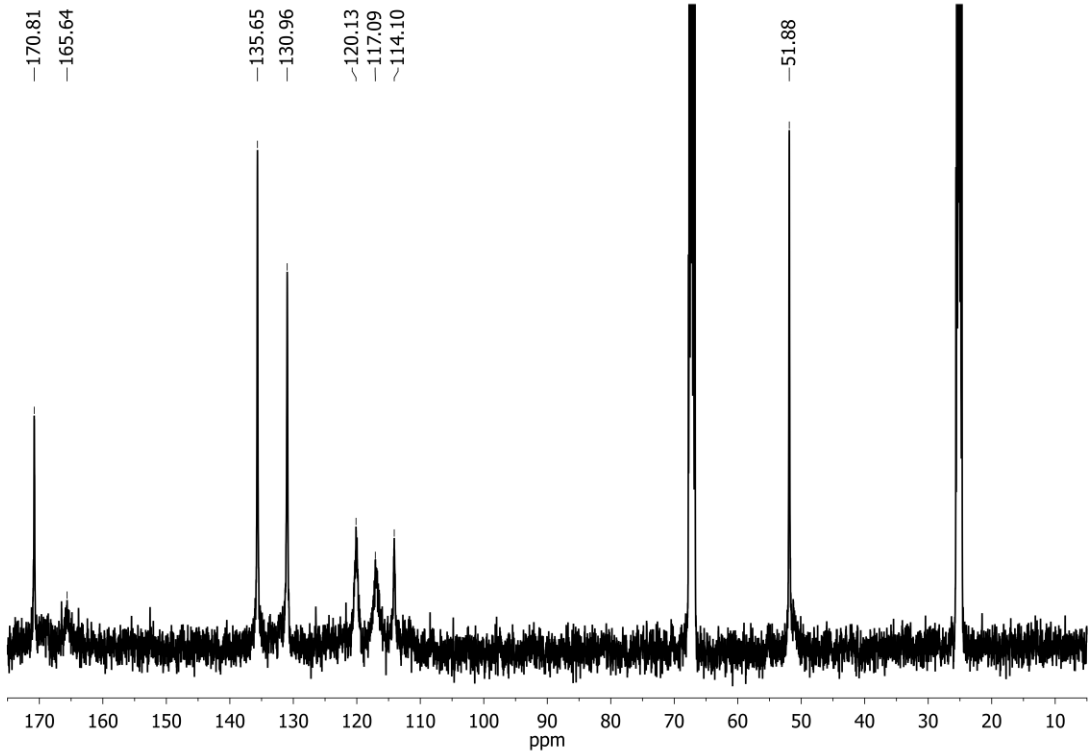

Figure S30. ^{13}C NMR spectrum of **8a** in THF-d_8.

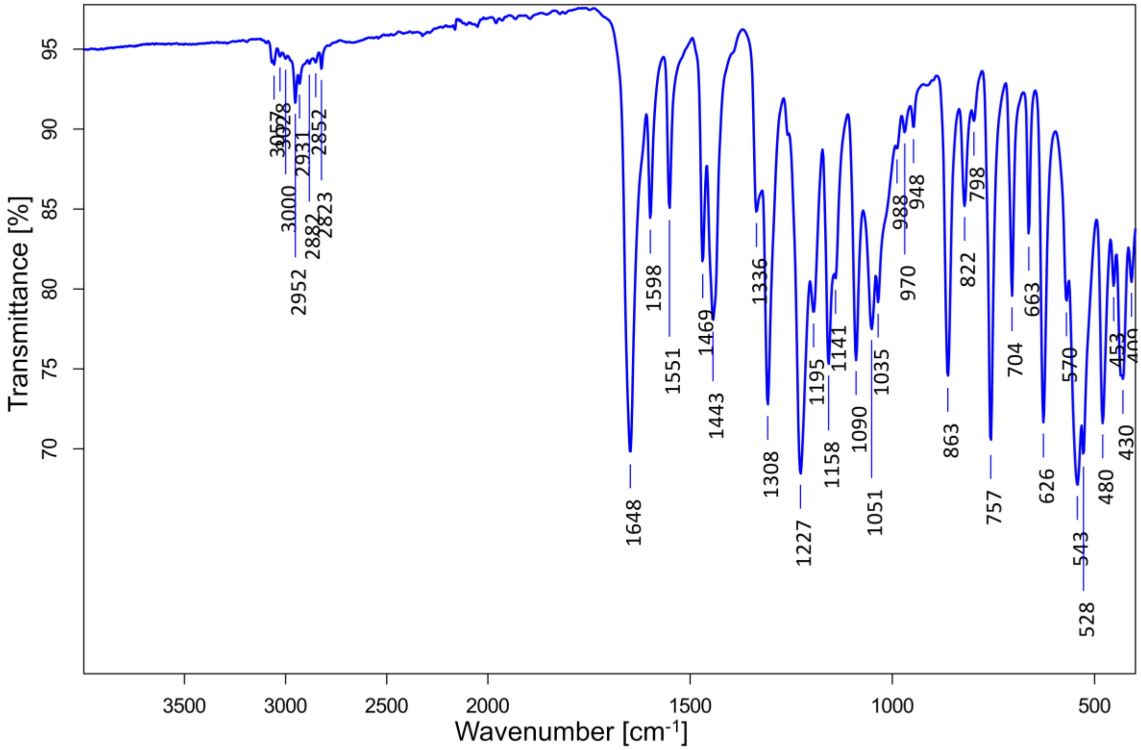

Figure S31. FTIR-ATR spectrum of **9**.

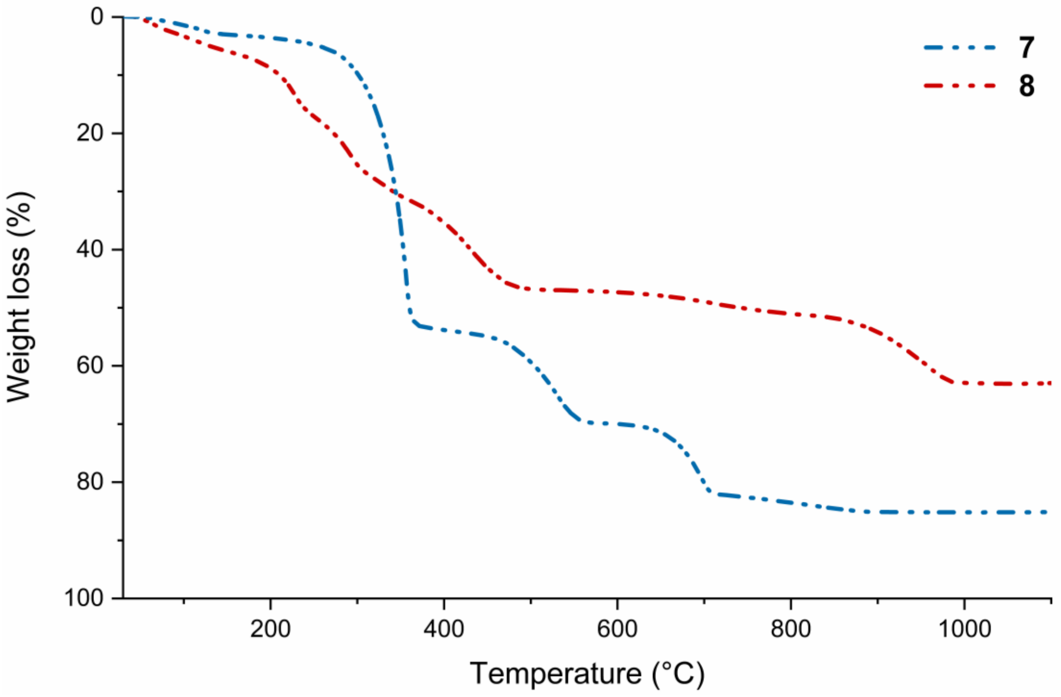

Figure S32. TGA curves for **7** and **8** measured at a heating rate of 5 °C min^{-1} under a nitrogen atmosphere over the temperature range of 25 to 1100 °C.

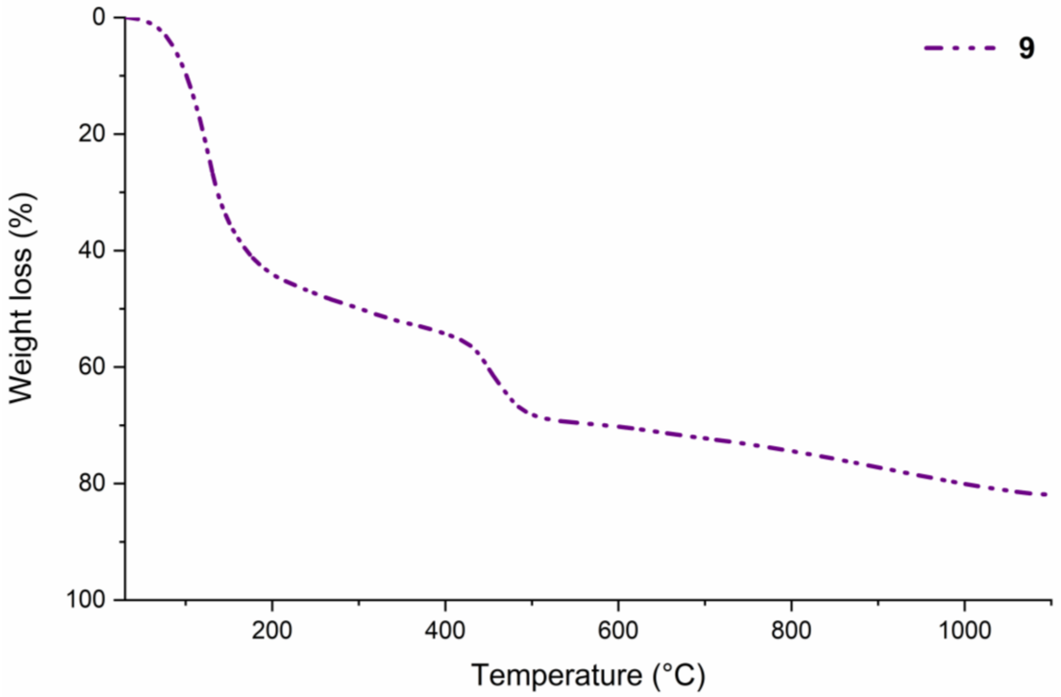

Figure S33. TGA curve for **9** measured at a heating rate of 5 °C min^{-1} under a nitrogen atmosphere over the temperature range of 25 to 1100 °C.

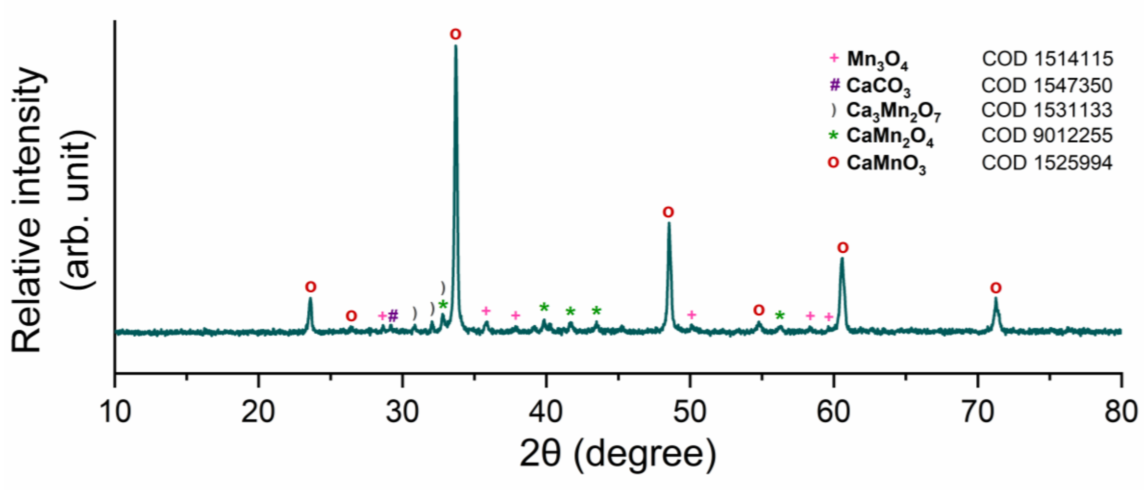

Figure S34. PXRD pattern of oxide material prepared by calcination of **7** with **9** in the presence of 0.22 mol % of **1** at 1100 °C.

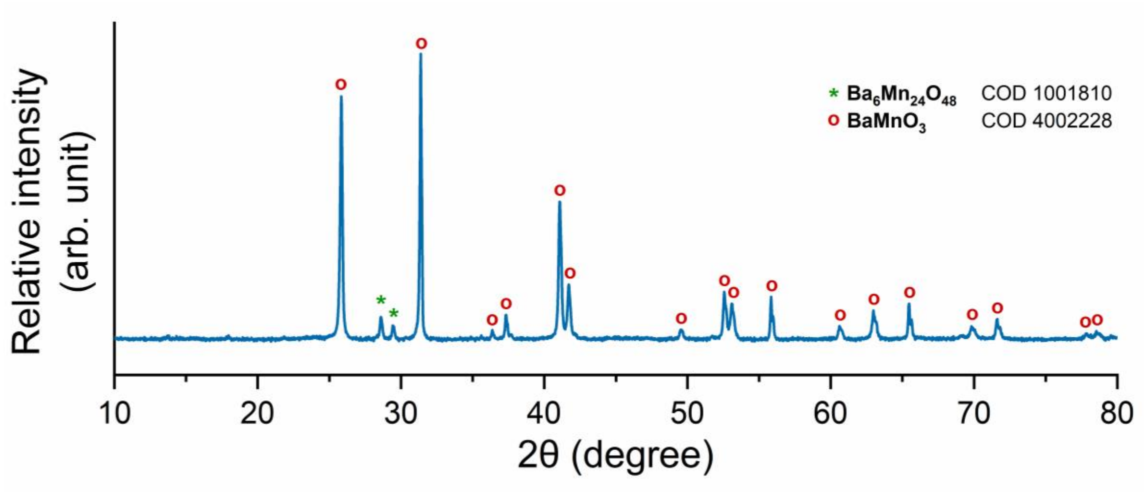

Figure S35. PXRD pattern of oxide material prepared by calcination of **8** with **9** in the presence of 0.22 mol % of **1** at 1100 °C.

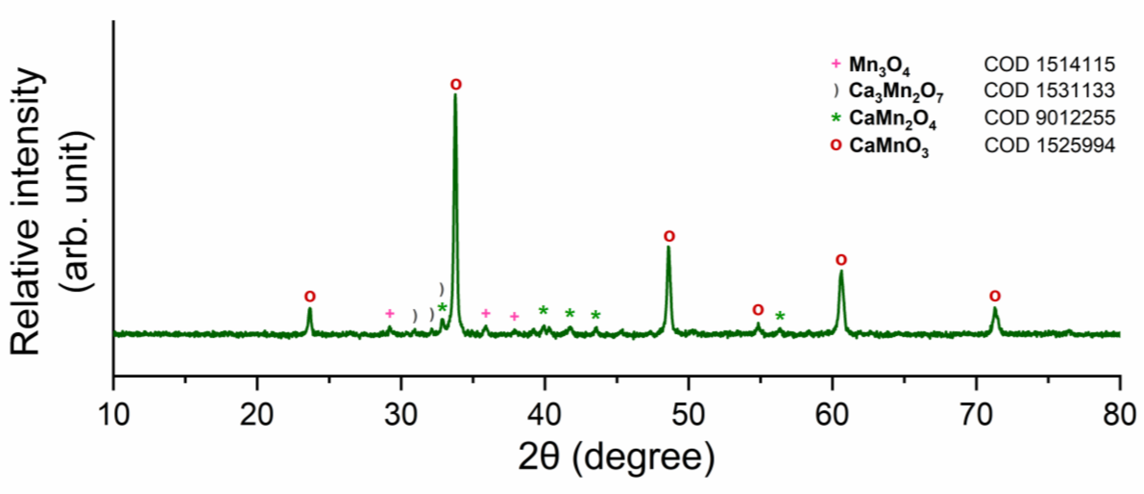

Figure S36. PXRD pattern of oxide material prepared by calcination of **7** with **9** in the presence of 0.22 mol % of **3** at 1100 °C.

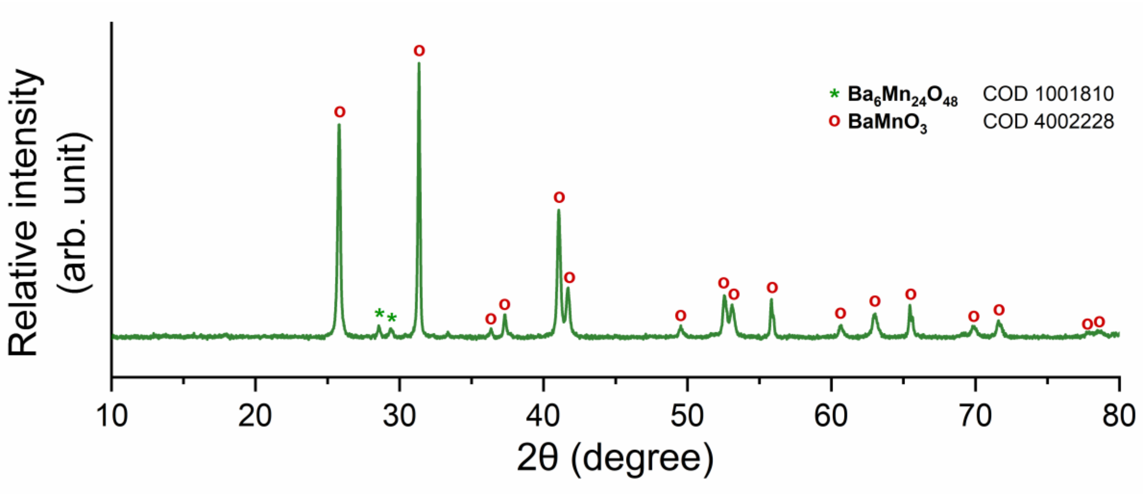

Figure S37. PXRD pattern of oxide material prepared by calcination of **8** with **9** in the presence of 0.22 mol % of **3** at 1100 °C.

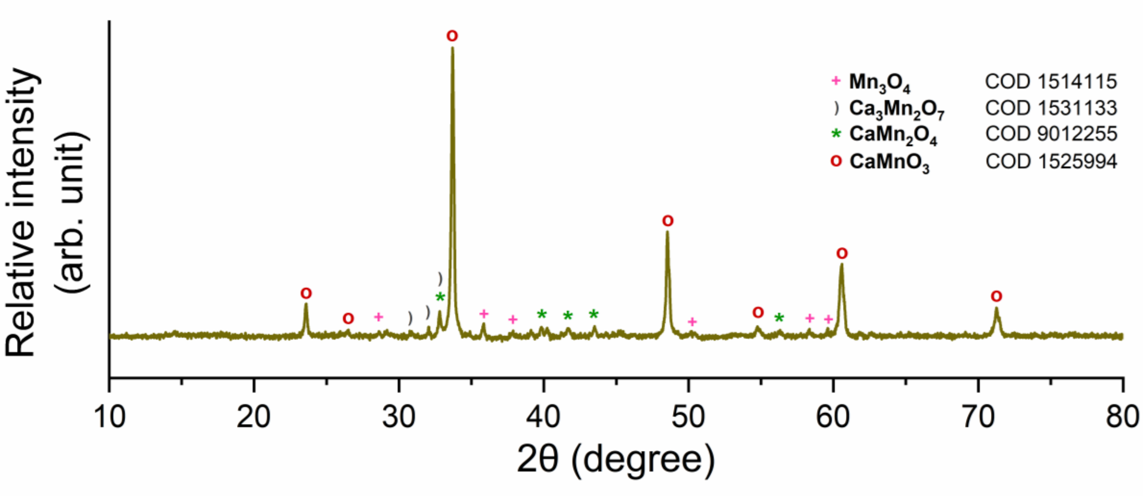

Figure S38. PXRD pattern of oxide material prepared by calcination of **7** with **9** in the presence of 0.22 mol % of **4** at 1100 °C.

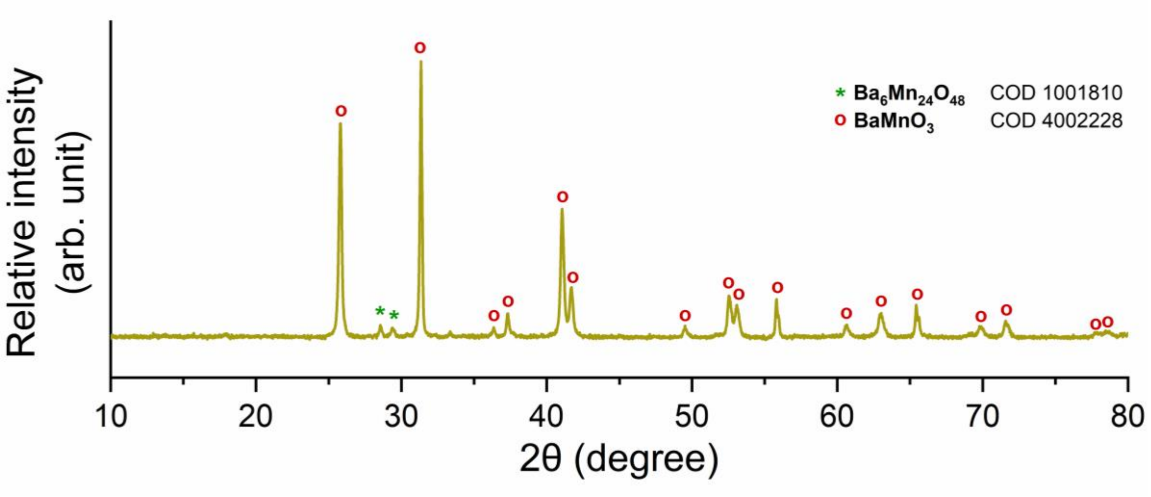

Figure S39. PXRD pattern of oxide material prepared by calcination of **8** with **9** in the presence of 0.22 mol % of **4** at 1100 °C.

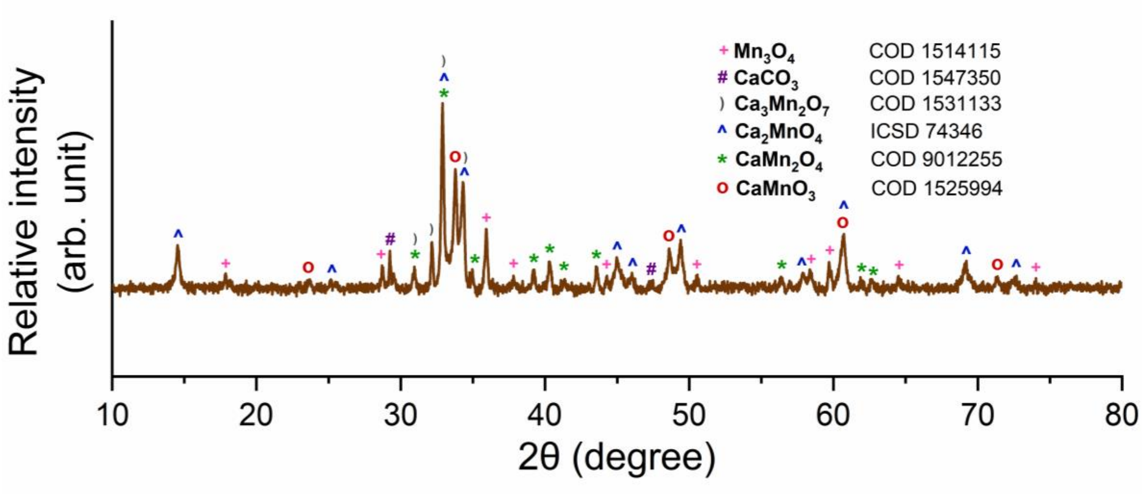

Figure S40. PXRD pattern of oxide material prepared by calcination of **7** with **9** in the presence of 0.22 mol % of **5** at 1100 °C.

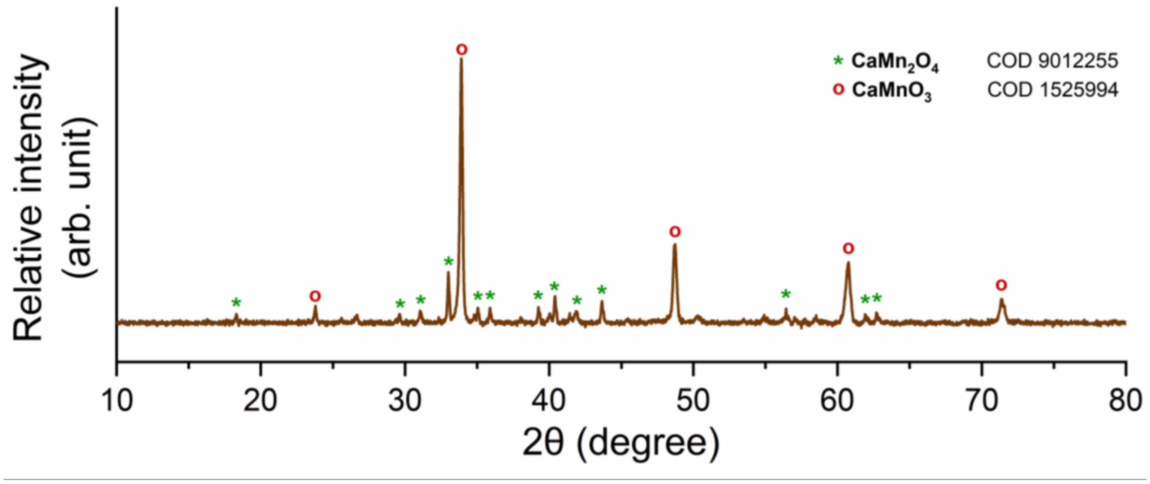

Figure S41. PXRD pattern of oxide material prepared by calcination of **7** with **9** in the presence of 1.11 mol % of **5** at 1100 °C.

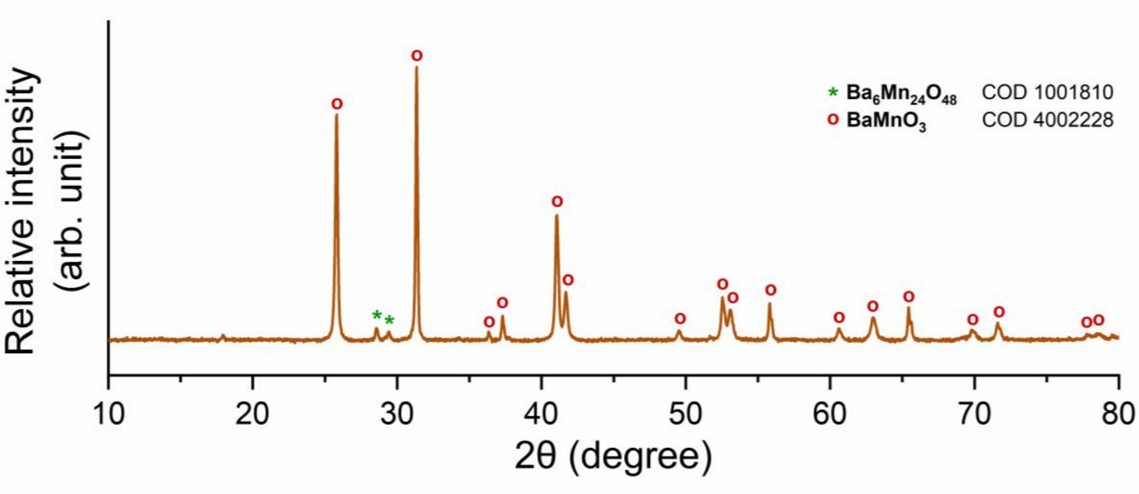

Figure S42. PXRD pattern of oxide material prepared by calcination of **8** with **9** in the presence of 0.22 mol % of **5** at 1100 °C.

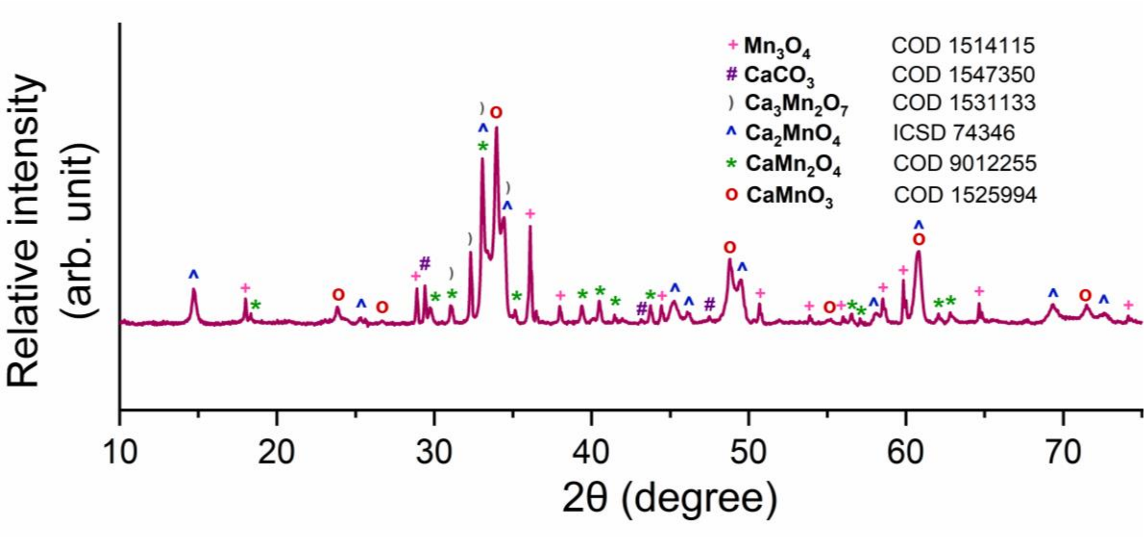

Figure S43. PXRD pattern of oxide material prepared by calcination of **7** with **9** in the presence of 0.22 mol % of **6** at 1100 °C.

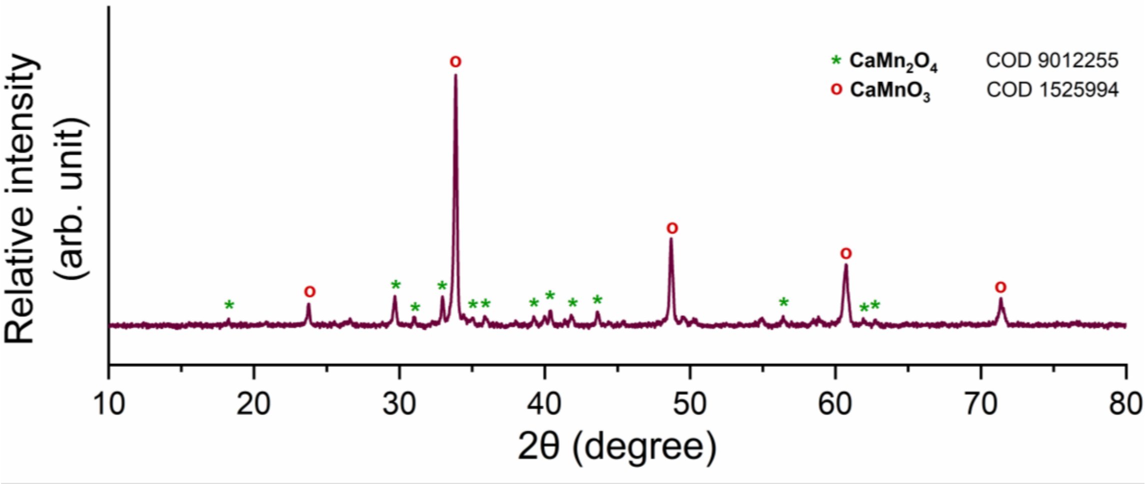

Figure S44. PXRD pattern of oxide material prepared by calcination of **7** with **9** in the presence of 1.11 mol % of **6** at 1100 °C.

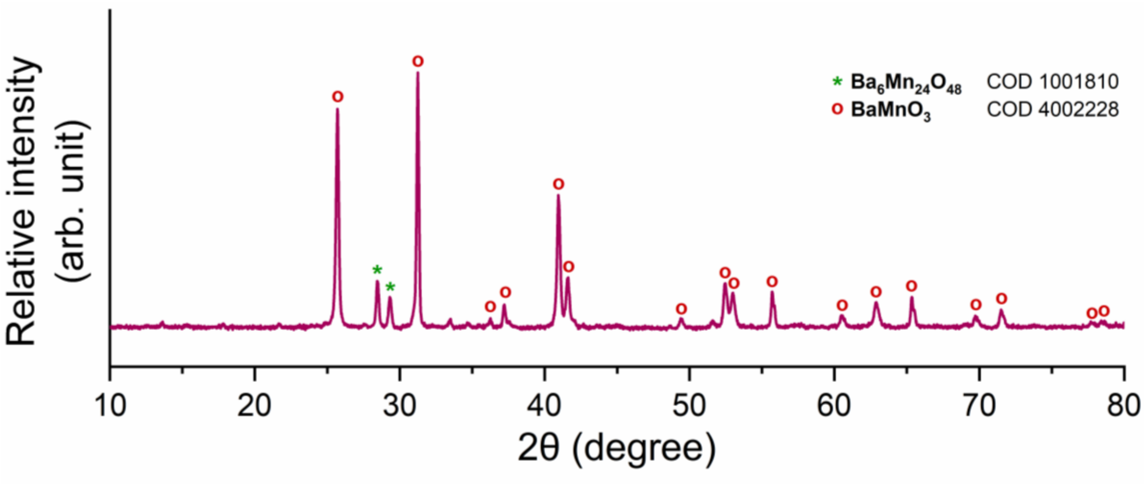

Figure S45. PXRD pattern of oxide material prepared by calcination of **8** with **9** in the presence of 0.22 mol % of **6** at 1100 °C.

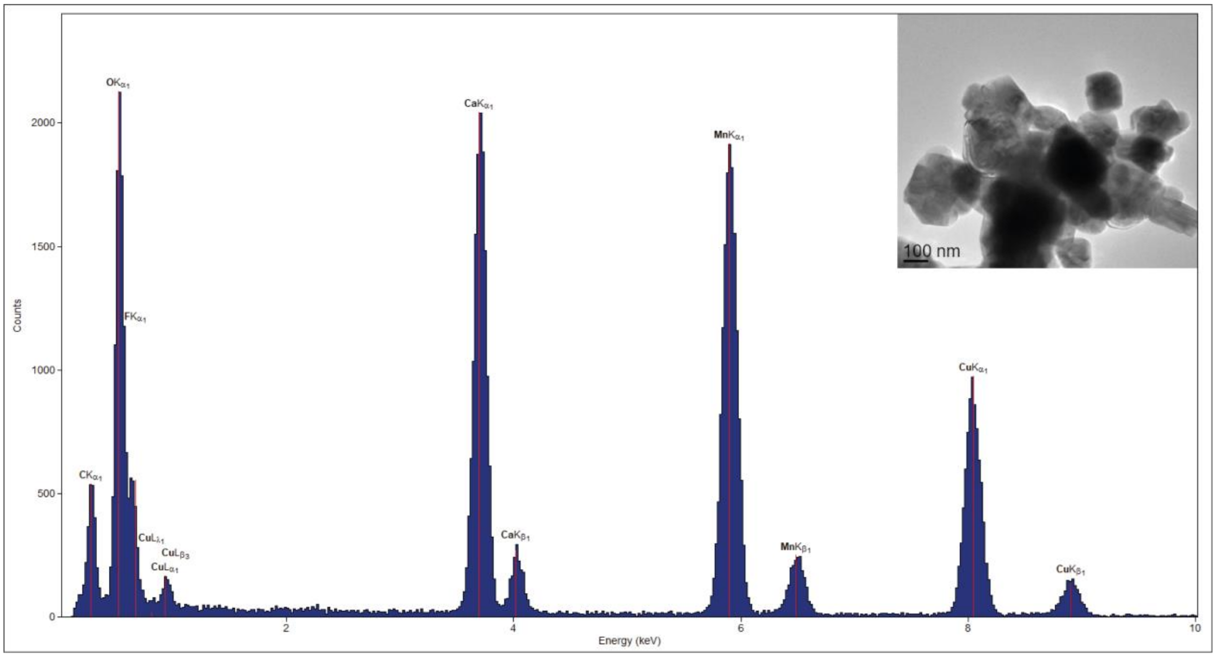

Figure S46. EDX analysis of $CaMnO_3$ crystallites. The copper and carbon elements come from the use of copper-carbon grids.

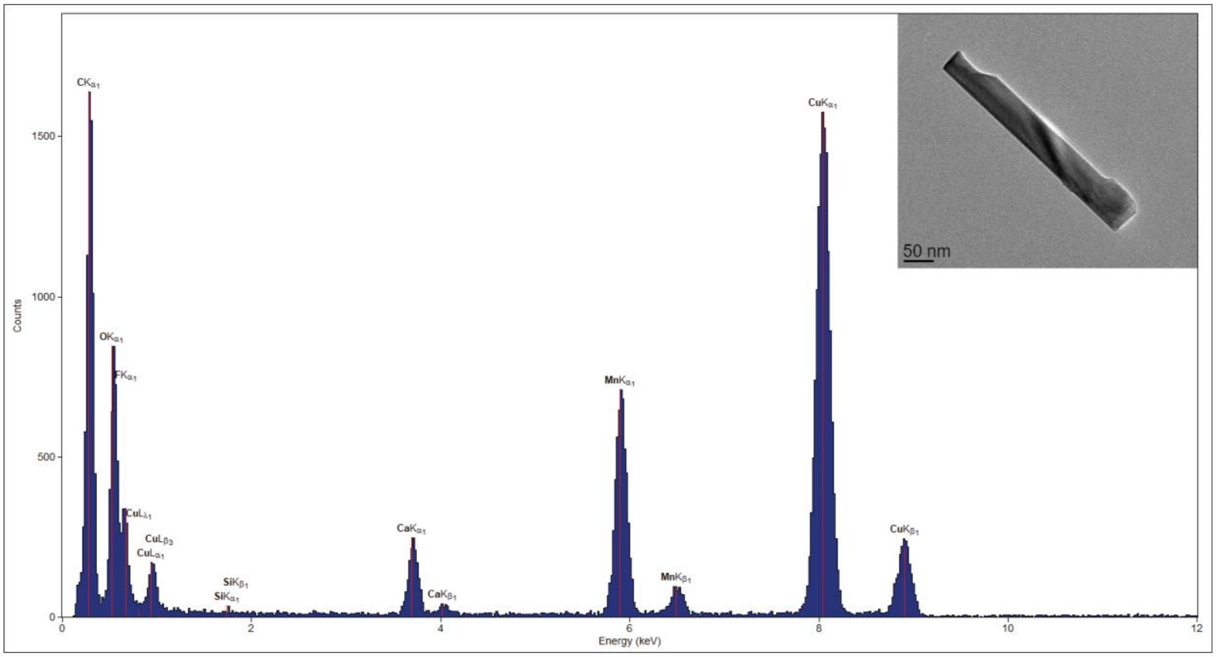

Figure S47. EDX analysis of $CaMn_2O_4$ crystallite. The copper and carbon elements come from the use of copper-carbon grids.

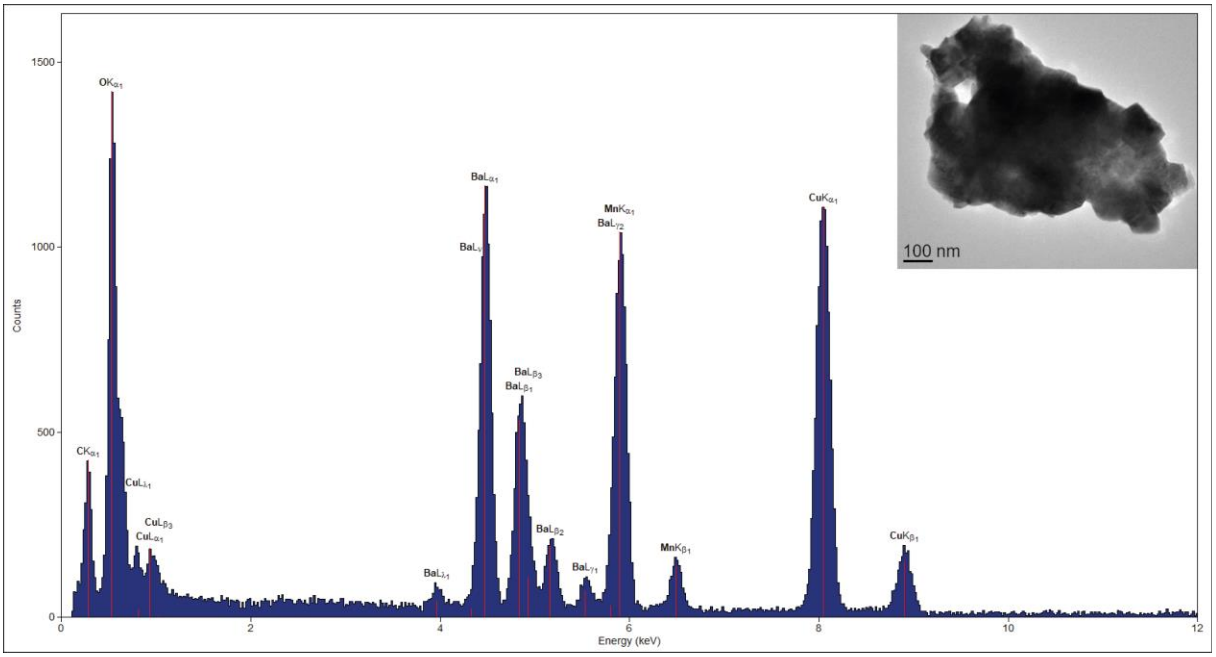

Figure S48. EDX analysis of $BaMnO_3$ crystallites. The copper and carbon elements come from the use of copper-carbon grids.

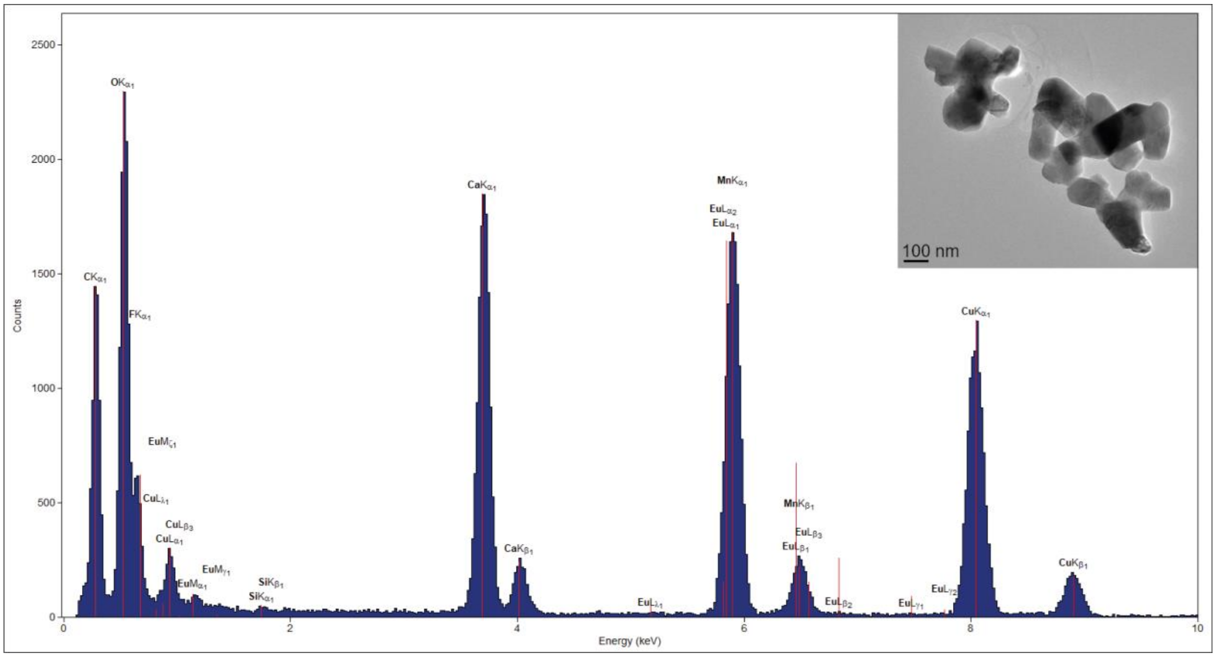

Figure S49. EDX analysis of $CaMnO_3$:Eu(III) crystallites. The copper and carbon elements come from the use of copper-carbon grids.

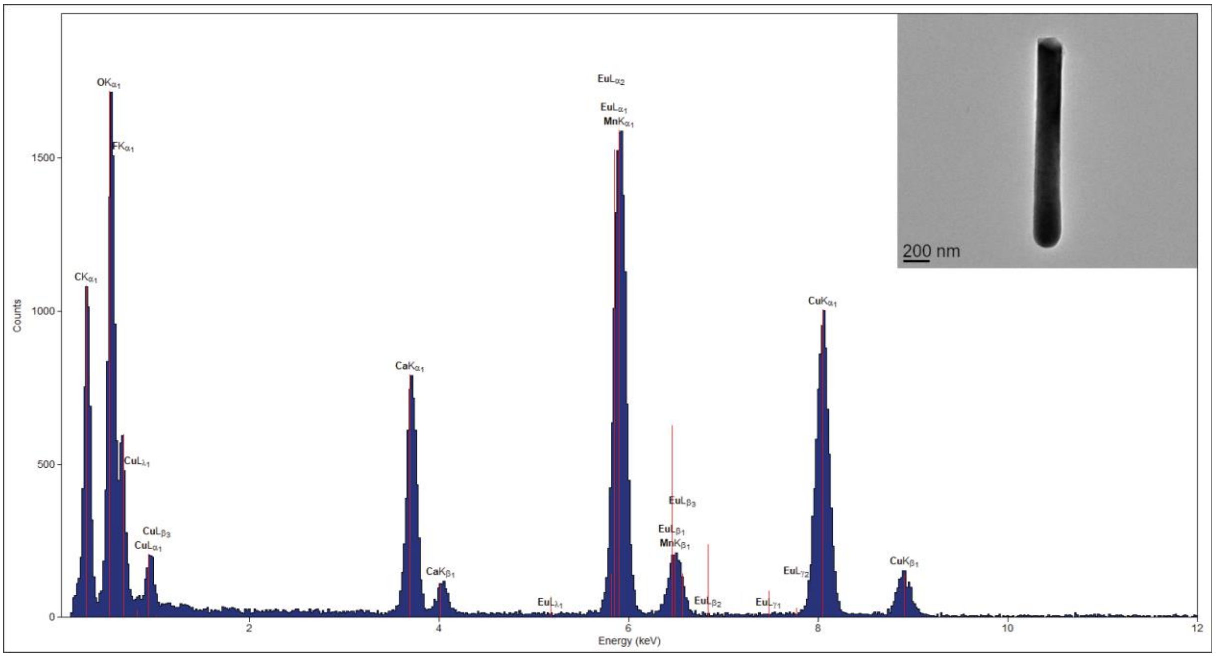

Figure S50. EDX analysis of $CaMn_2O_4$:Eu(III) crystallite. The copper and carbon elements come from the use of copper-carbon grids.

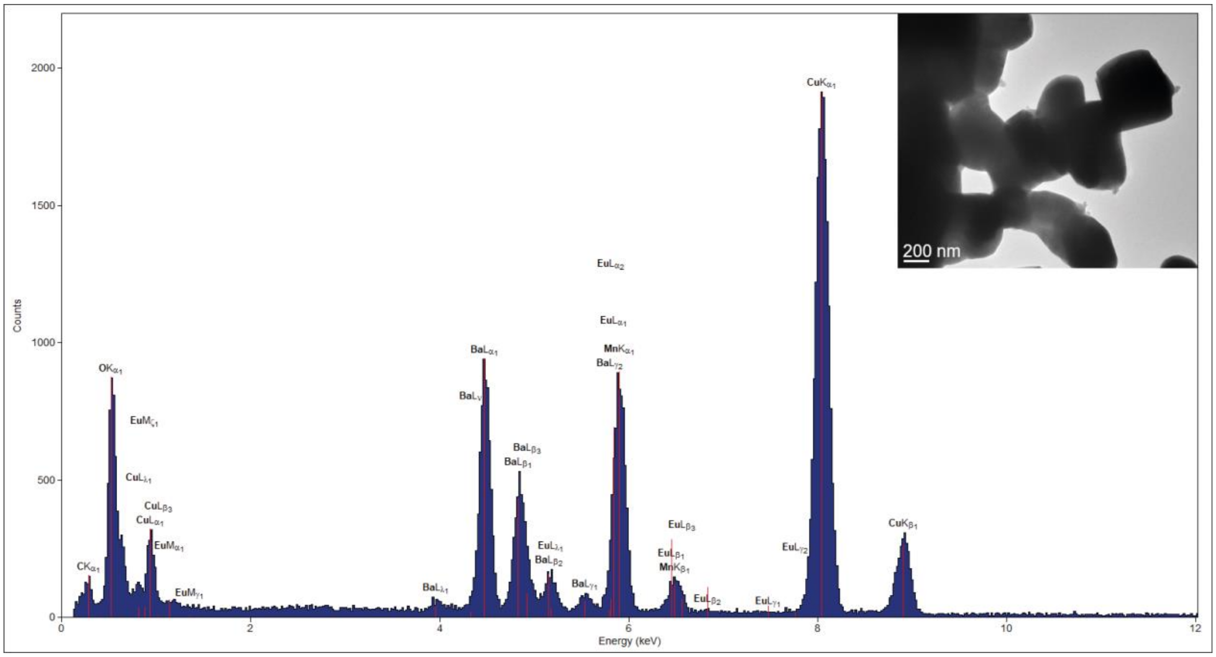

Figure S51. EDX analysis of $BaMnO_3$:Eu(III) crystallites. The copper and carbon elements come from the use of copper-carbon grids.

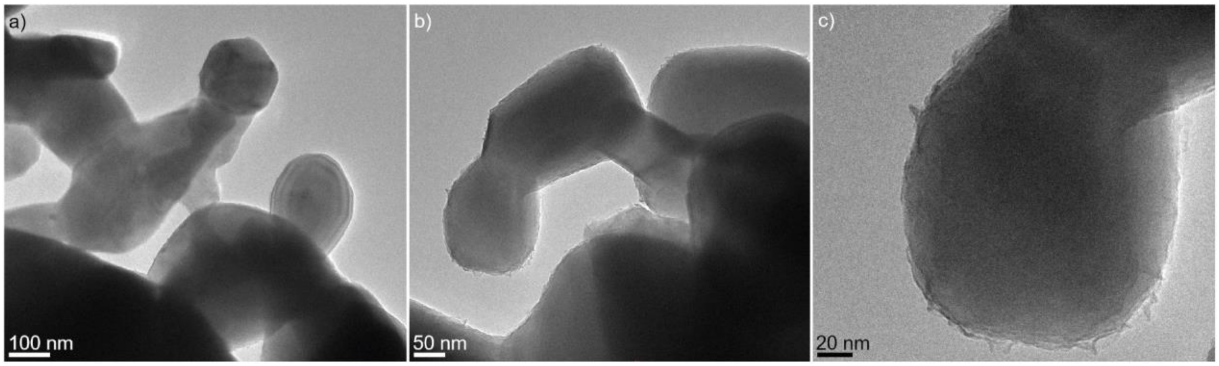

Figure S52. TEM images of oxide materials prepared by calcination of **7** with **9** in the presence of 0.22 mol % of **1** (a–c) at 1100 °C.

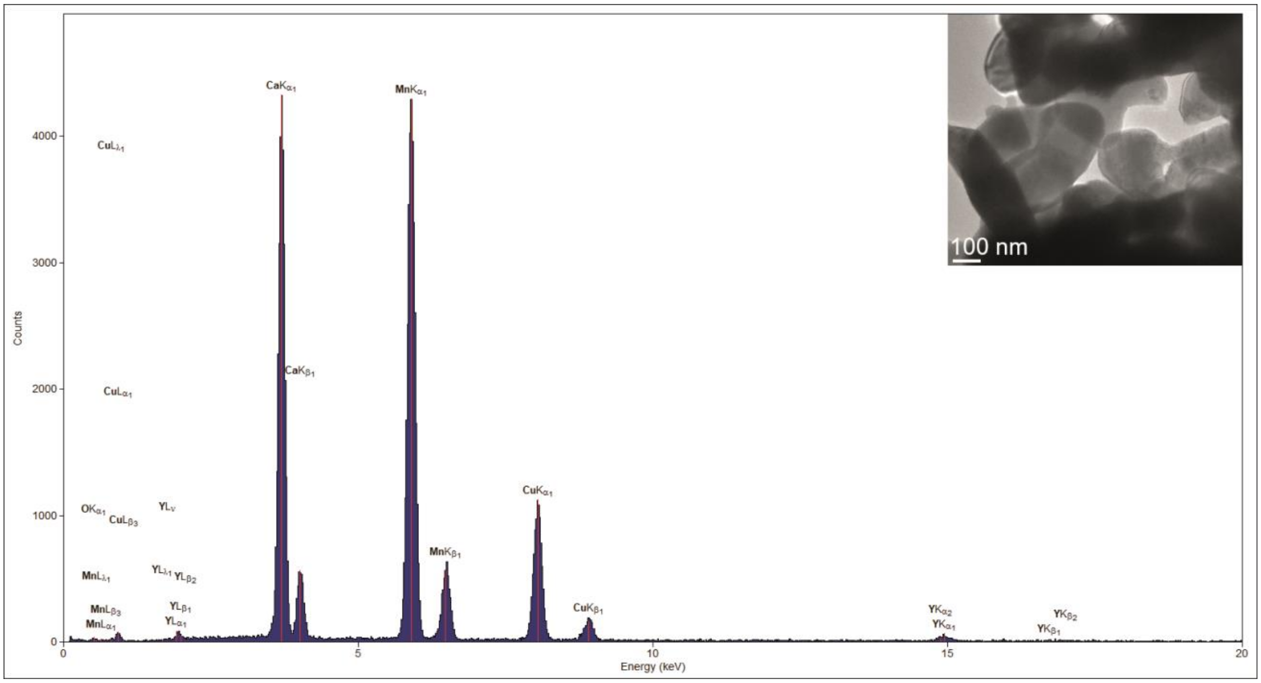

Figure S53. EDX analysis of $CaMnO_3$:Y(III) crystallites. The copper and carbon elements come from the use of copper-carbon grids.

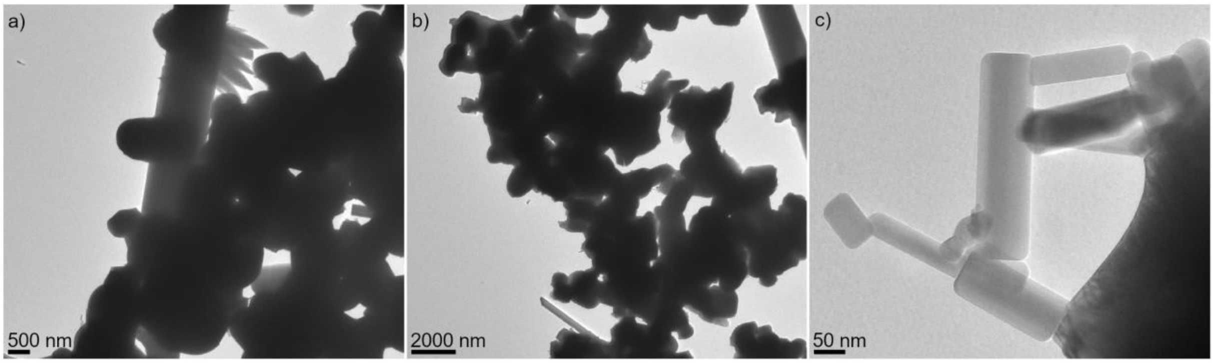

Figure S54. TEM images of oxide materials prepared by calcination of **8** with **9** in the presence of 0.22 mol % of **1** (a–c) at 1100 °C.

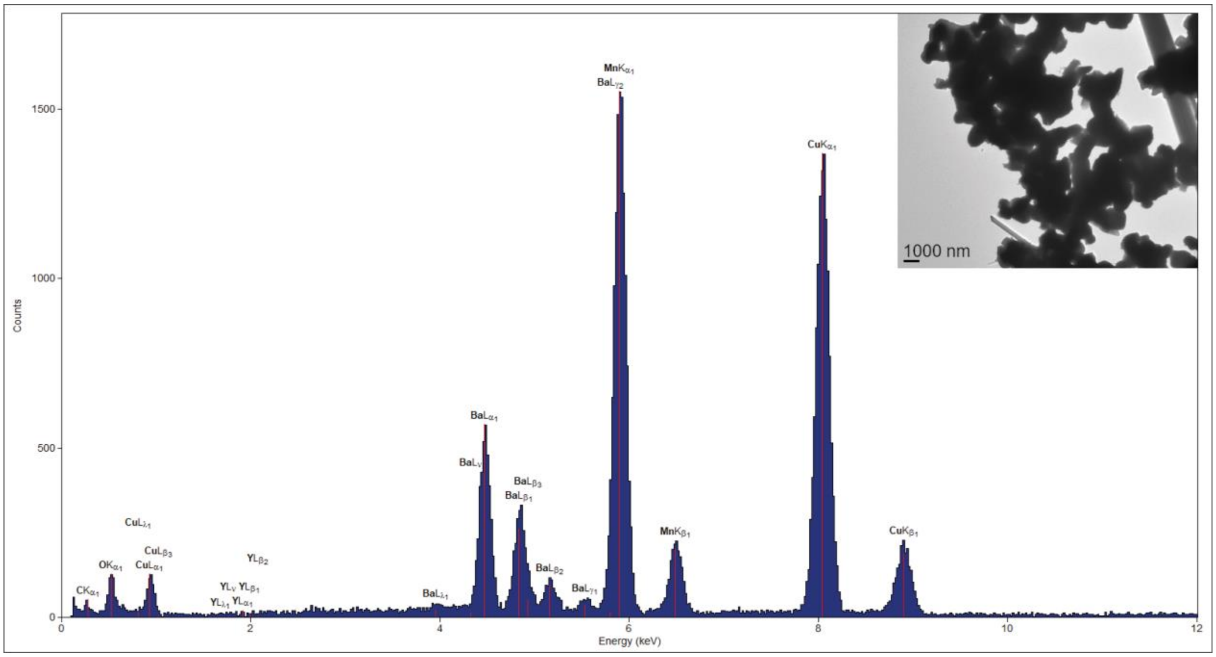

Figure S55. EDX analysis of $BaMnO_3$:Y(III) material. The copper and carbon elements come from the use of copper-carbon grids.

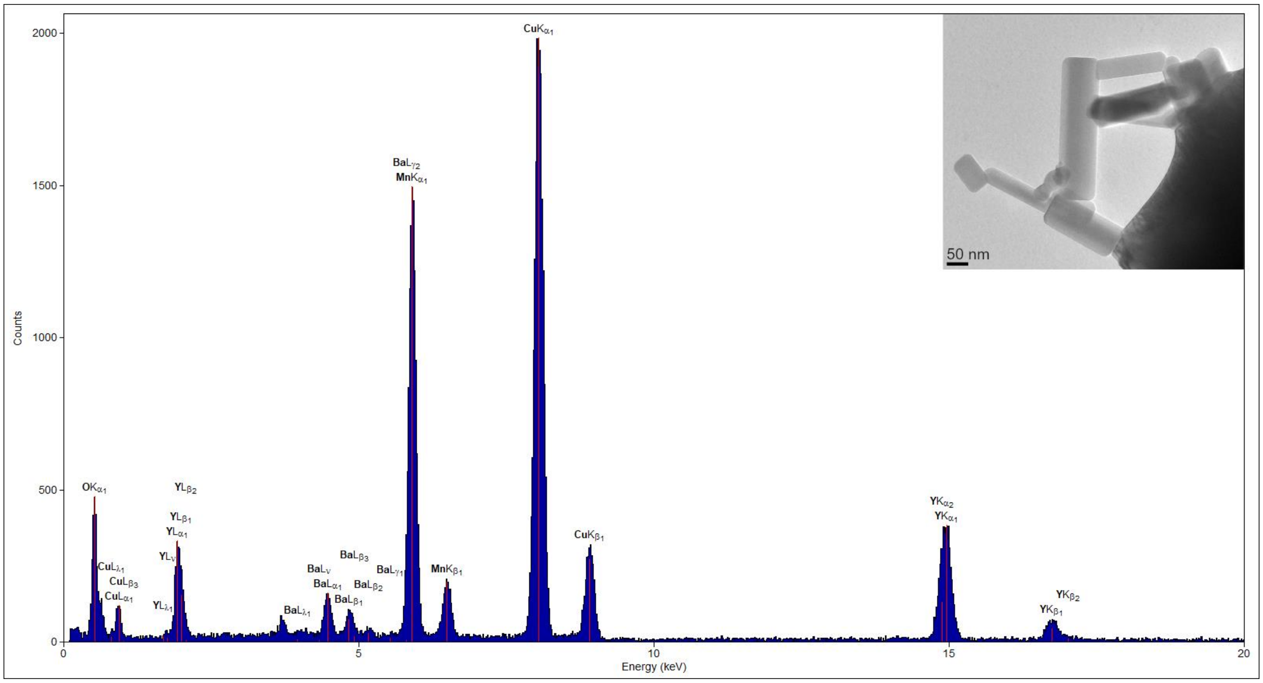

Figure S56. EDX analysis of $YMnO_3$ rod-like crystallites found in oxide materials prepared by calcination of **8** with **9** in the presence of 0.22 mol % of **1** at 1100 °C. The copper and carbon elements come from the use of copper-carbon grids.

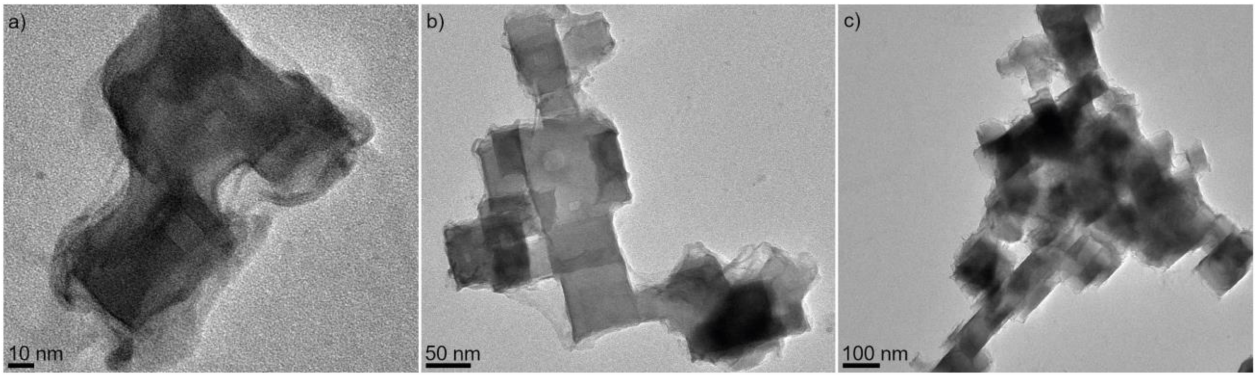

Figure S57. TEM images of oxide materials prepared by calcination of **7** with **9** in the presence of 0.22 mol % of **3** (a–c) at 1100 °C.

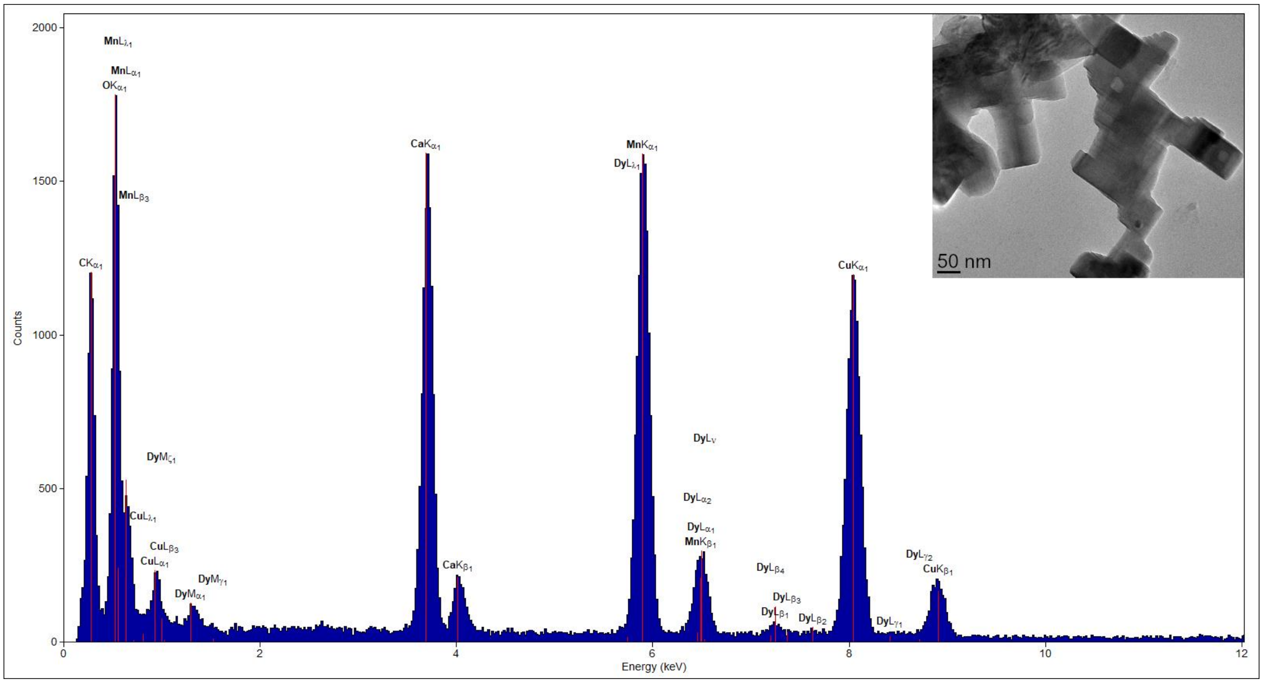

Figure S58. EDX analysis of $CaMnO_3$:Dy(III) crystallites. The copper and carbon elements come from the use of copper-carbon grids.

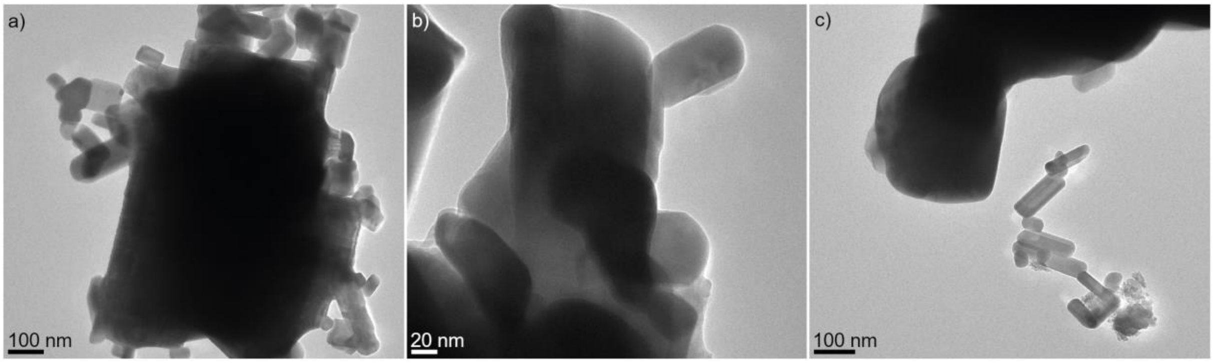

Figure S59. TEM images of oxide materials prepared by calcination of **8** with **9** in the presence of 0.22 mol % of **3** (a–c) at 1100 °C.

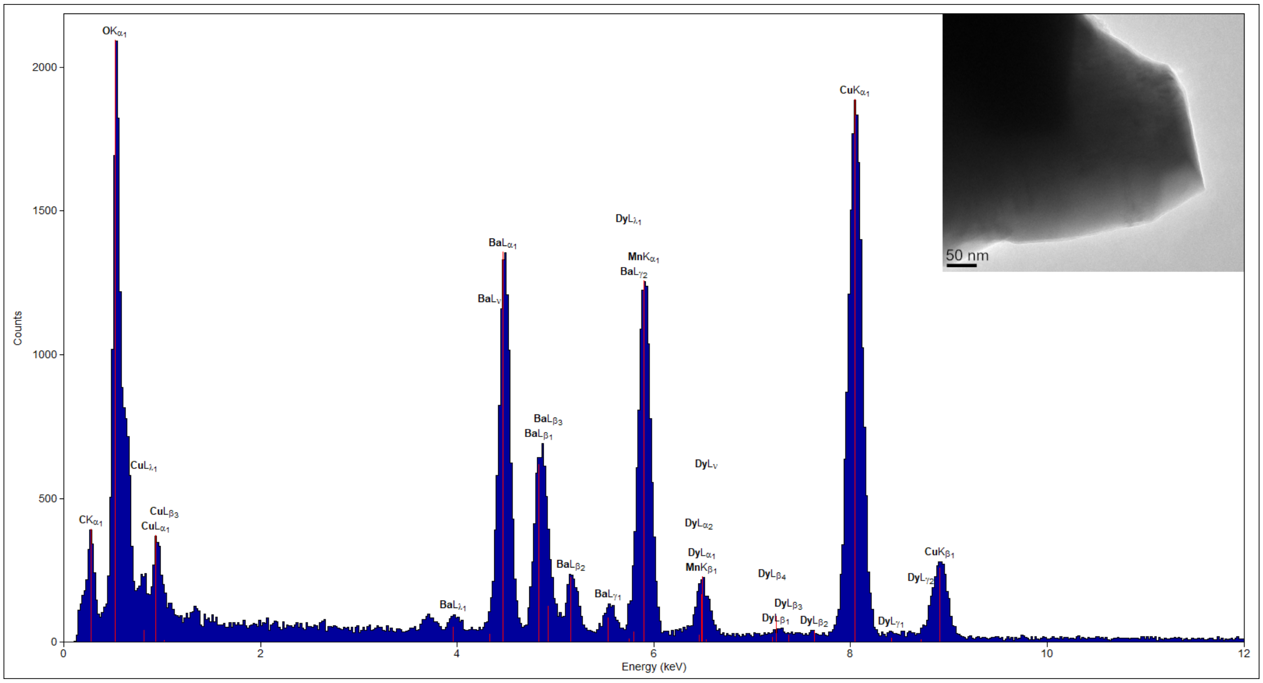

Figure S60. EDX analysis of $BaMnO_3$:Dy(III) crystallite. The copper and carbon elements come from the use of copper-carbon grids.

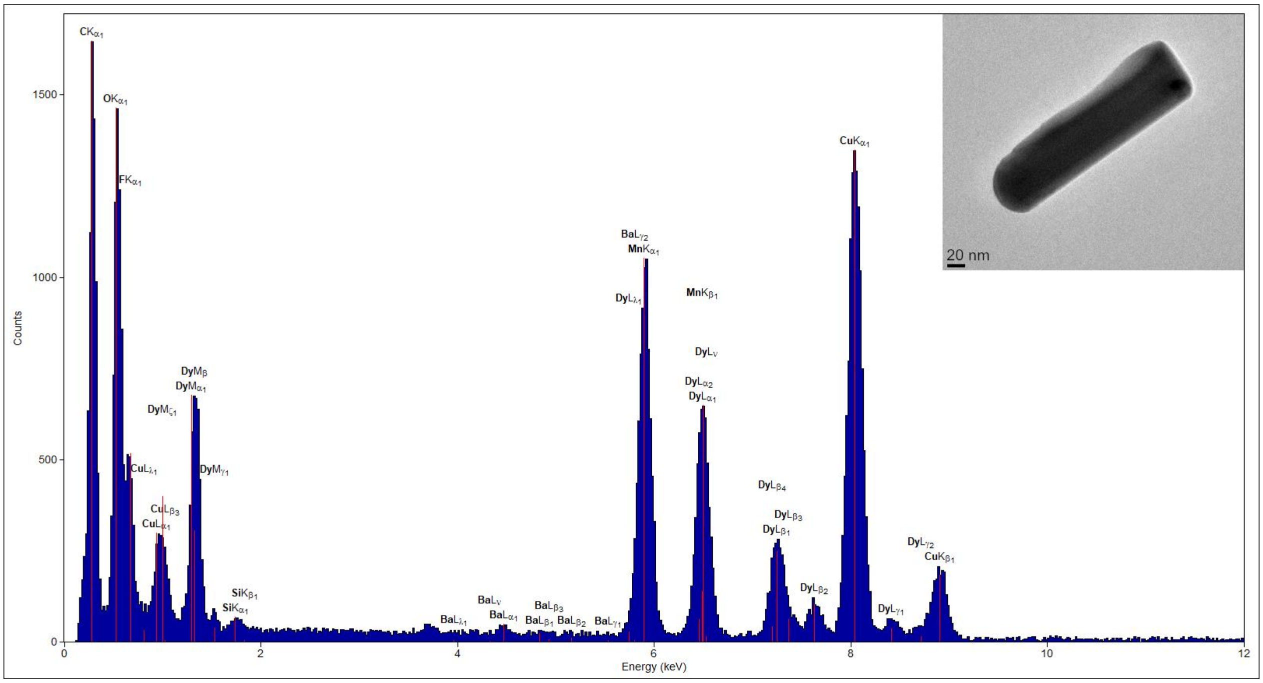

Figure S61. EDX analysis of $DyMnO_3$ rod-like crystallite found in oxide materials prepared by calcination of **8** with **9** in the presence of 0.22 mol % of **3** at 1100 °C. The copper and carbon elements come from the use of copper-carbon grids.

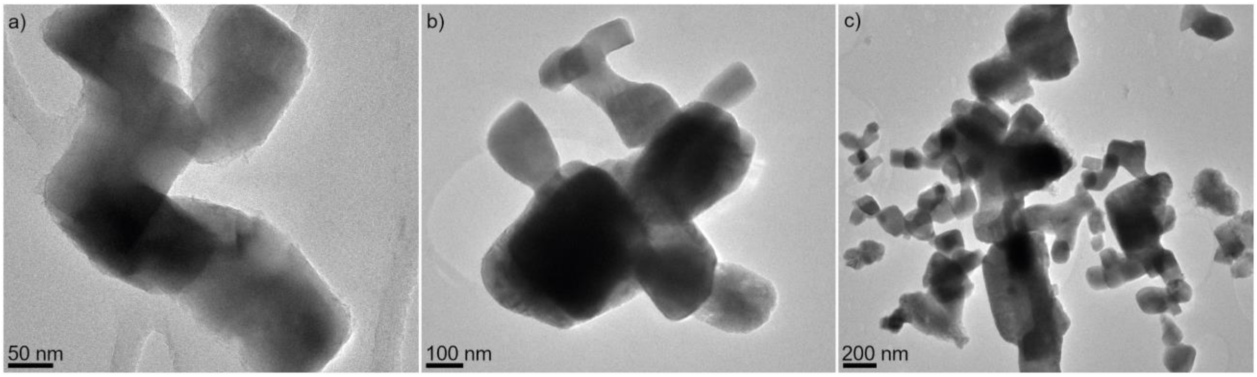

Figure S62. TEM images of oxide materials prepared by calcination of **7** with **9** in the presence of 0.22 mol % of **4** (a–c) at 1100 °C.

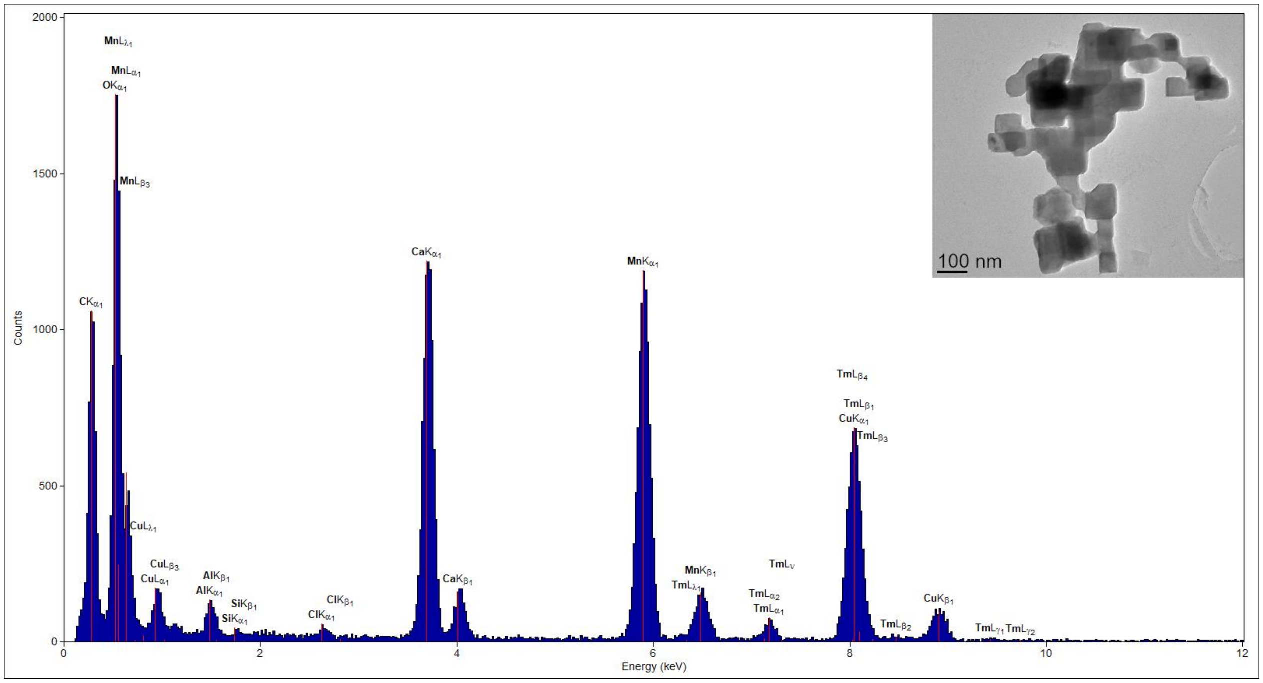

Figure S63. EDX analysis of $CaMnO_3$:Tm(III) crystallites. The copper and carbon elements come from the use of copper-carbon grids.

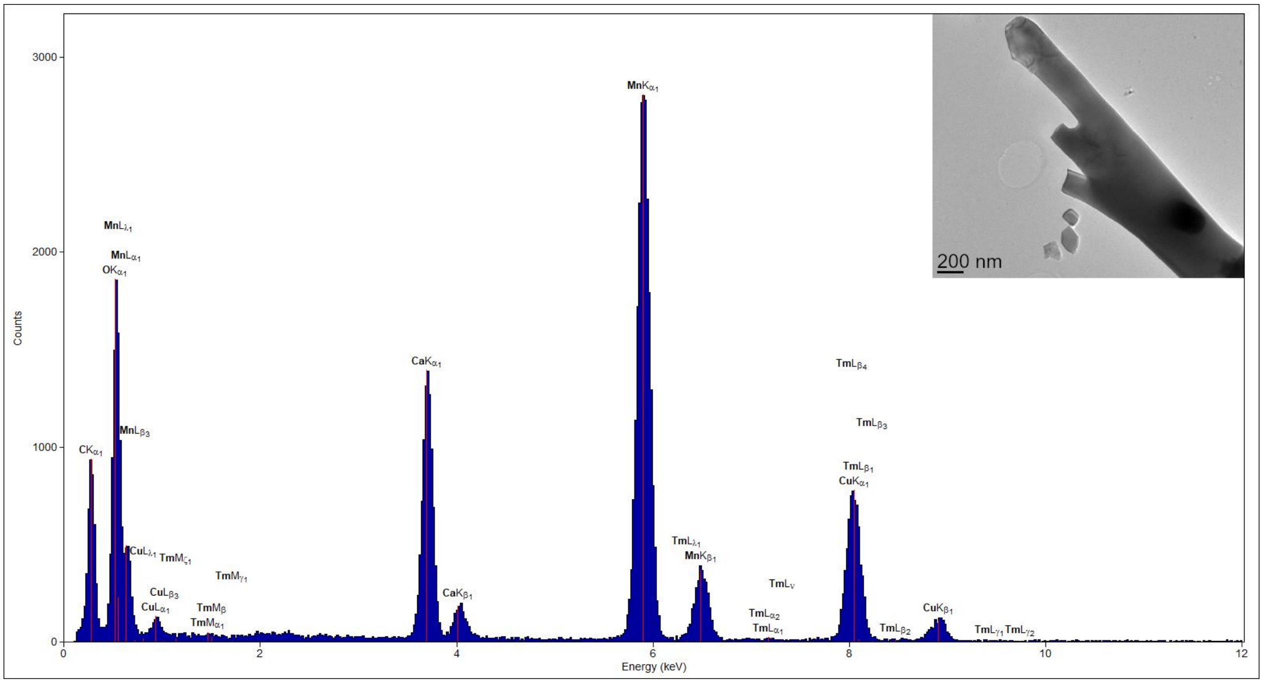

Figure S64. EDX analysis of $CaMn_2O_4$:Tm(III) crystallites. The copper and carbon elements come from the use of copper-carbon grids.

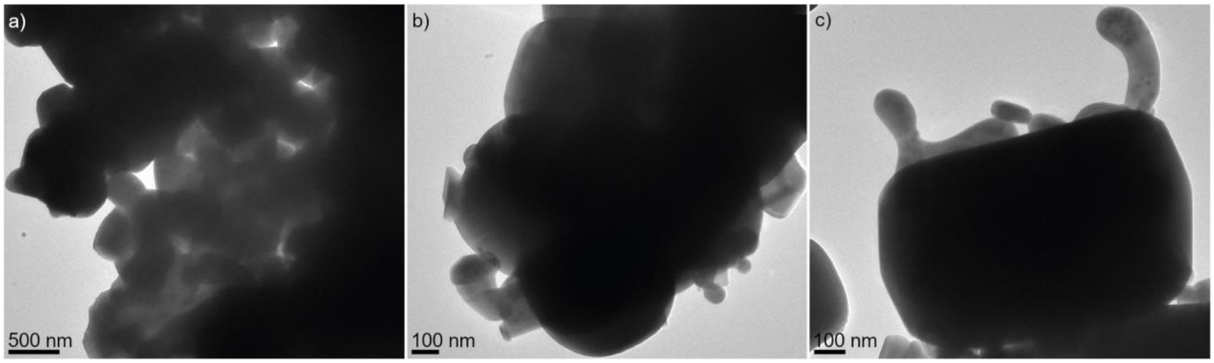

Figure S65. TEM images of oxide materials prepared by calcination of **8** with **9** in the presence of 0.22 mol % of **4** (a–c) at 1100 °C.

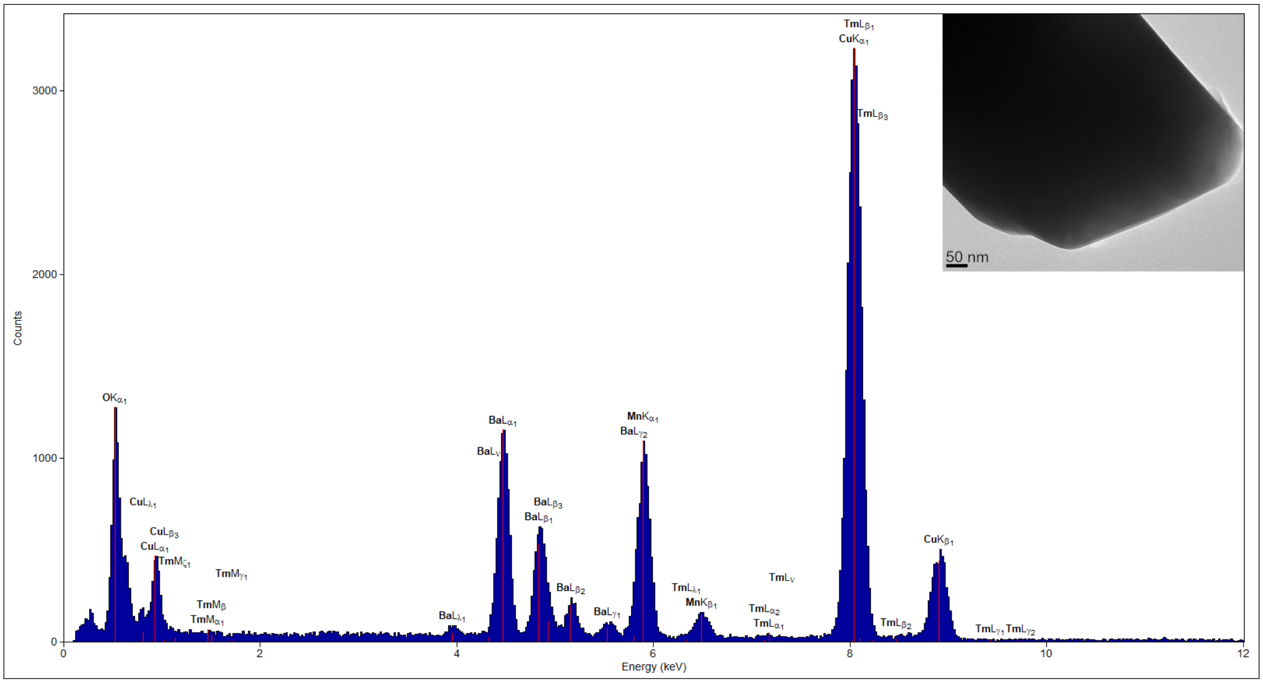

Figure S66. EDX analysis of $BaMnO_3$:Tm(III) crystallite. The copper and carbon elements come from the use of copper-carbon grids.

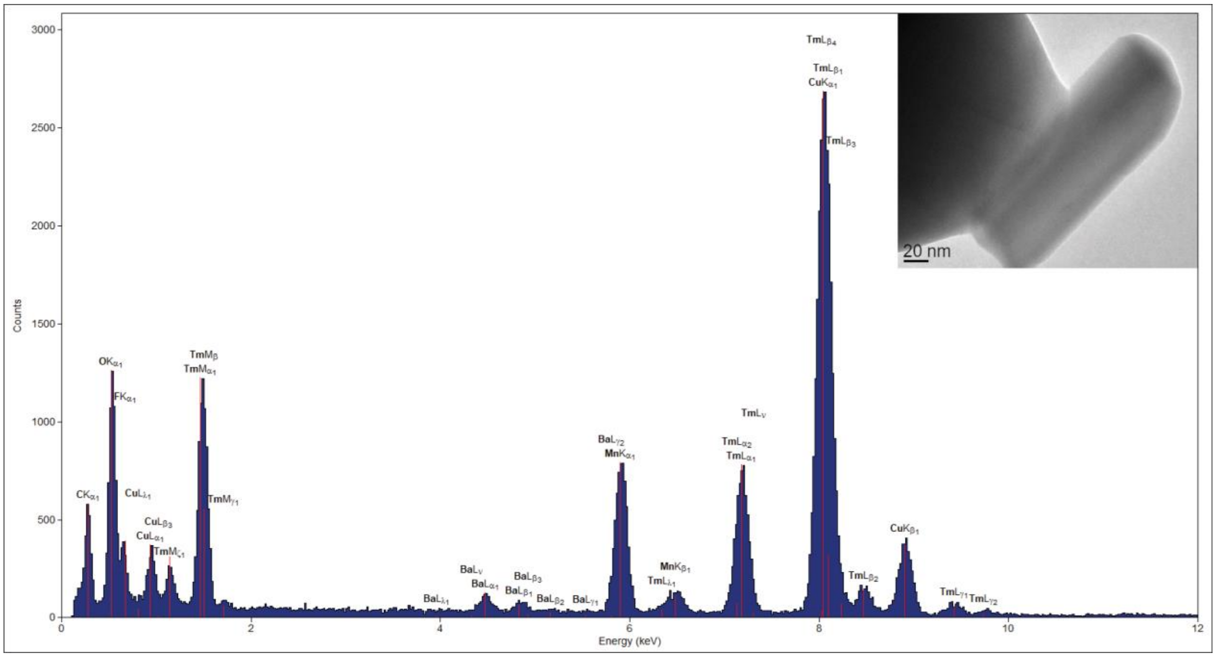

Figure S67. EDX analysis of $TmMnO_3$ rod-like crystallite found in oxide materials prepared by calcination of **8** with **9** in the presence of 0.22 mol % of **4** at 1100 °C. The copper and carbon elements come from the use of copper-carbon grids.

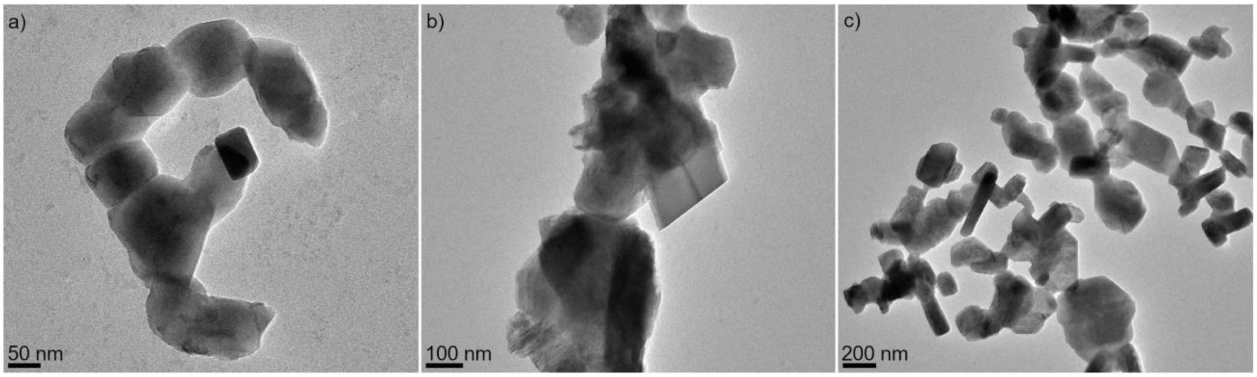

Figure S68. TEM images of oxide materials prepared by calcination of **7** with **9** in the presence of 0.22 mol % of **5** (a–c) at 1100 °C.

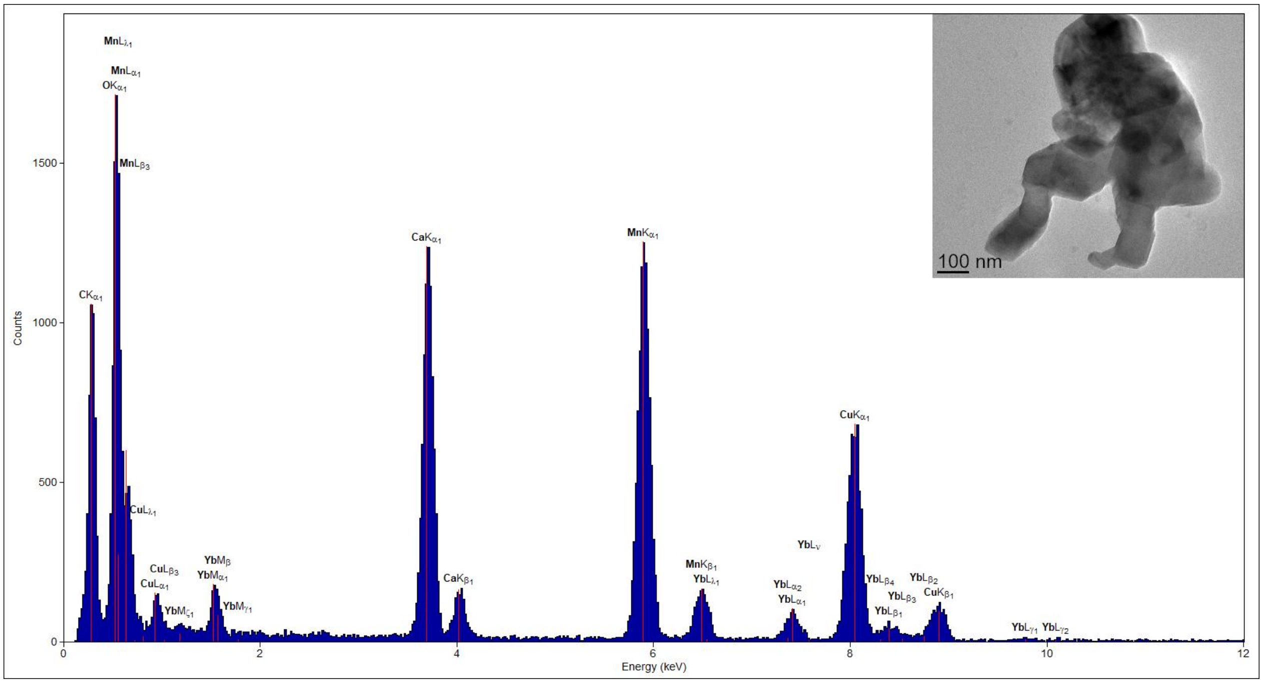

Figure S69. EDX analysis of $CaMnO_3$:Yb(III) crystallites. The copper and carbon elements come from the use of copper-carbon grids.

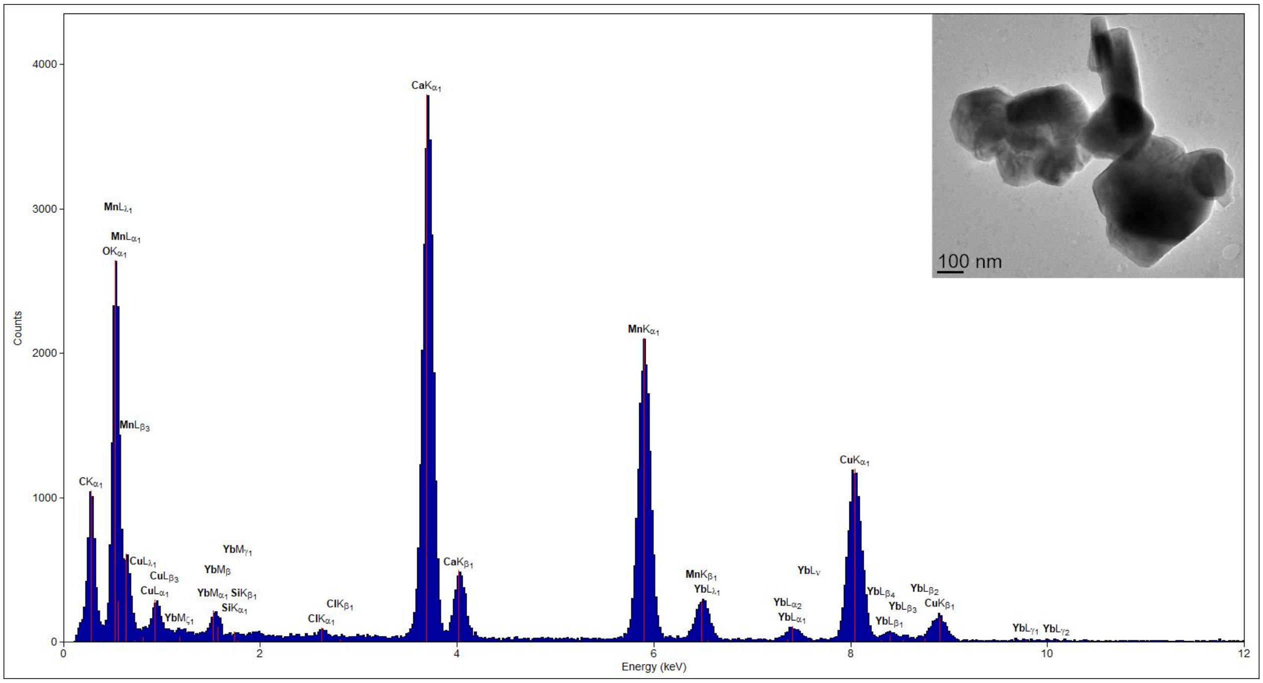

Figure S70. EDX analysis of Ca_2MnO_4:Yb(III) crystallites. The copper and carbon elements come from the use of copper-carbon grids.

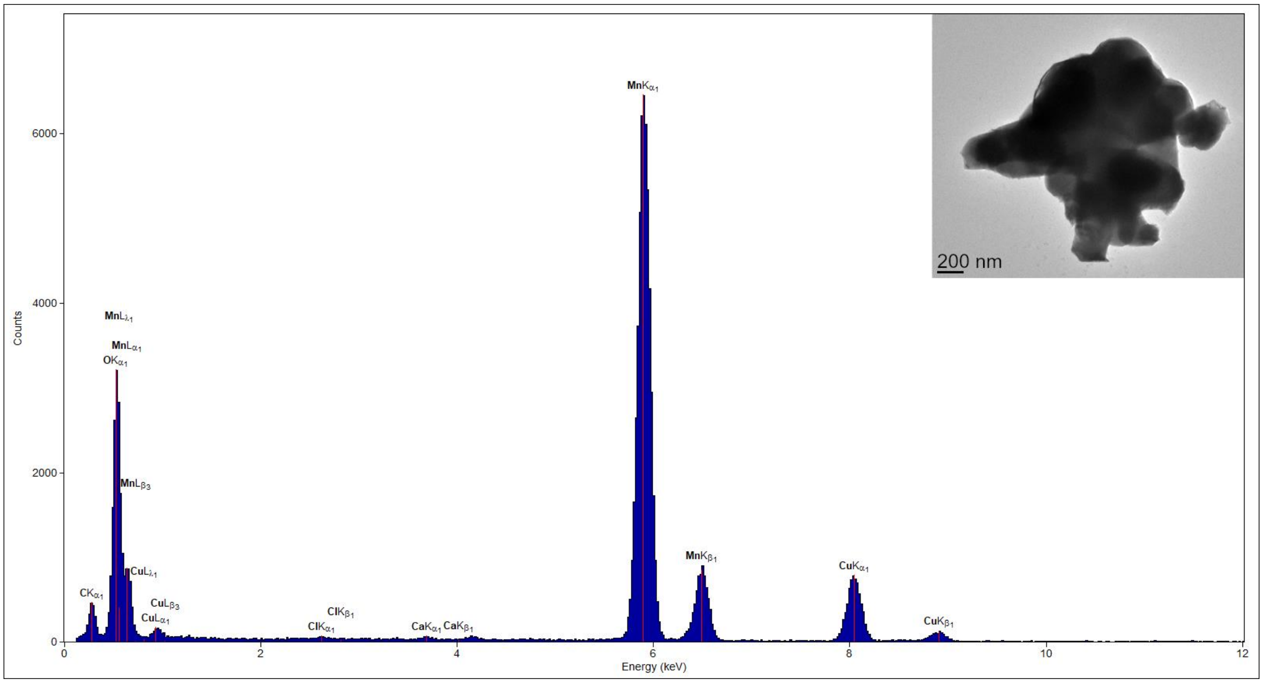

Figure S71. EDX analysis of Mn_3O_4 crystallites found in oxide materials prepared by calcination of **7** with **9** in the presence of 0.22 mol % of **5**. The copper and carbon elements come from the use of copper-carbon grids.

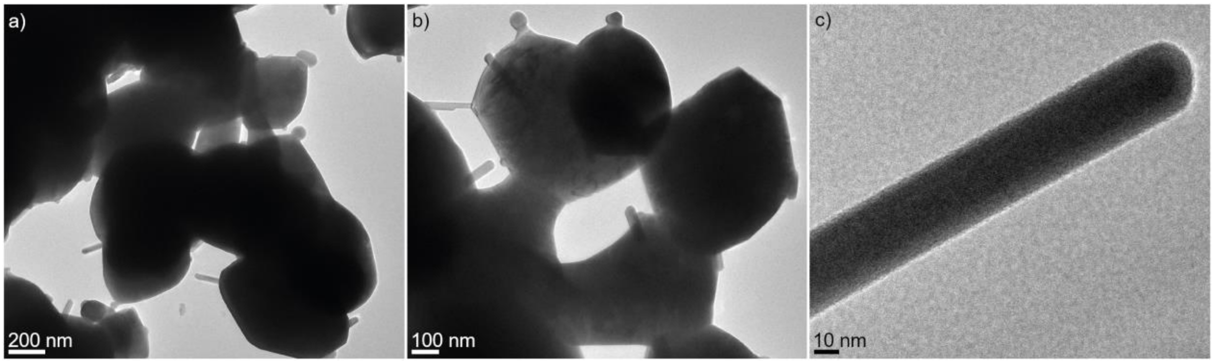

Figure S72. TEM images of oxide materials prepared by calcination of **8** with **9** in the presence of 0.22 mol % of **5** (a–c) at 1100 °C.

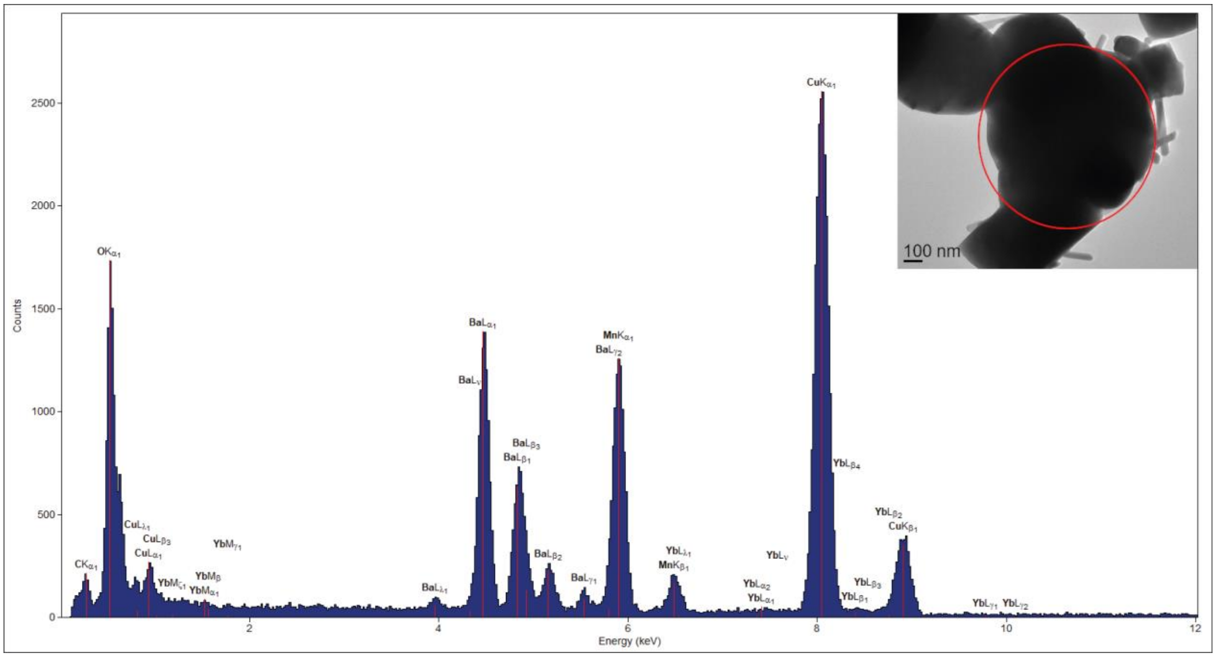

Figure S73. EDX analysis of $BaMnO_3$:Yb(III) crystallite. The copper and carbon elements come from the use of copper-carbon grids.

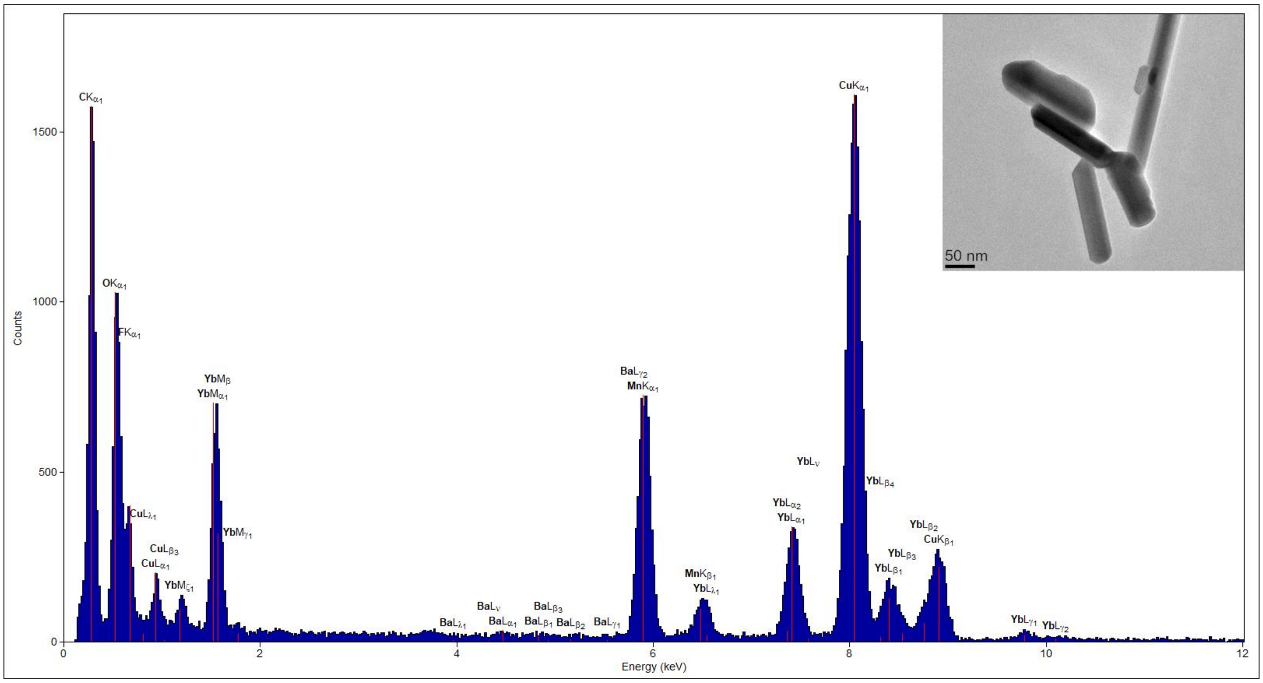

Figure S74. EDX analysis of $YbMnO_3$ rod-like crystallite found in oxide materials prepared by calcination of **8** with **9** in the presence of 0.22 mol % of **5** at 1100 °C. The copper and carbon elements come from the use of copper-carbon grids.

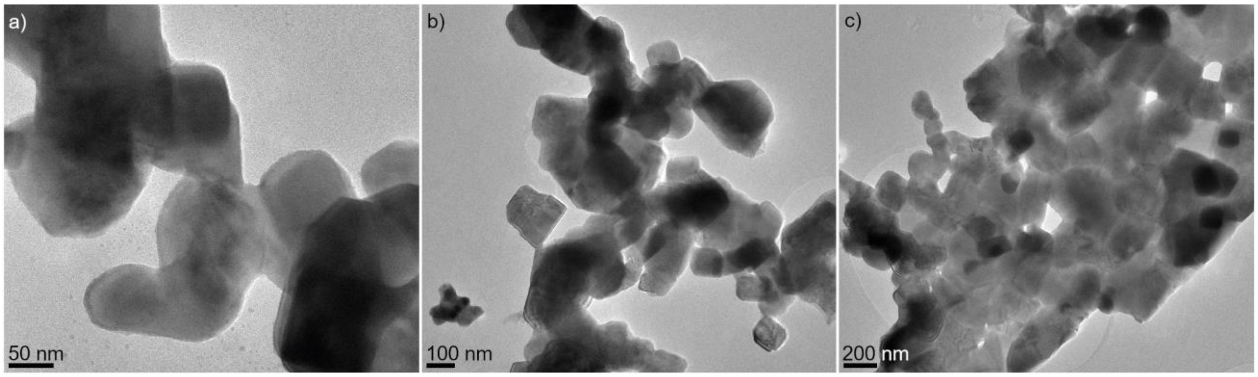

Figure S75. TEM images of oxide materials prepared by calcination of **7** with **9** in the presence of 0.22 mol % of **6** (a–c) at 1100 °C.

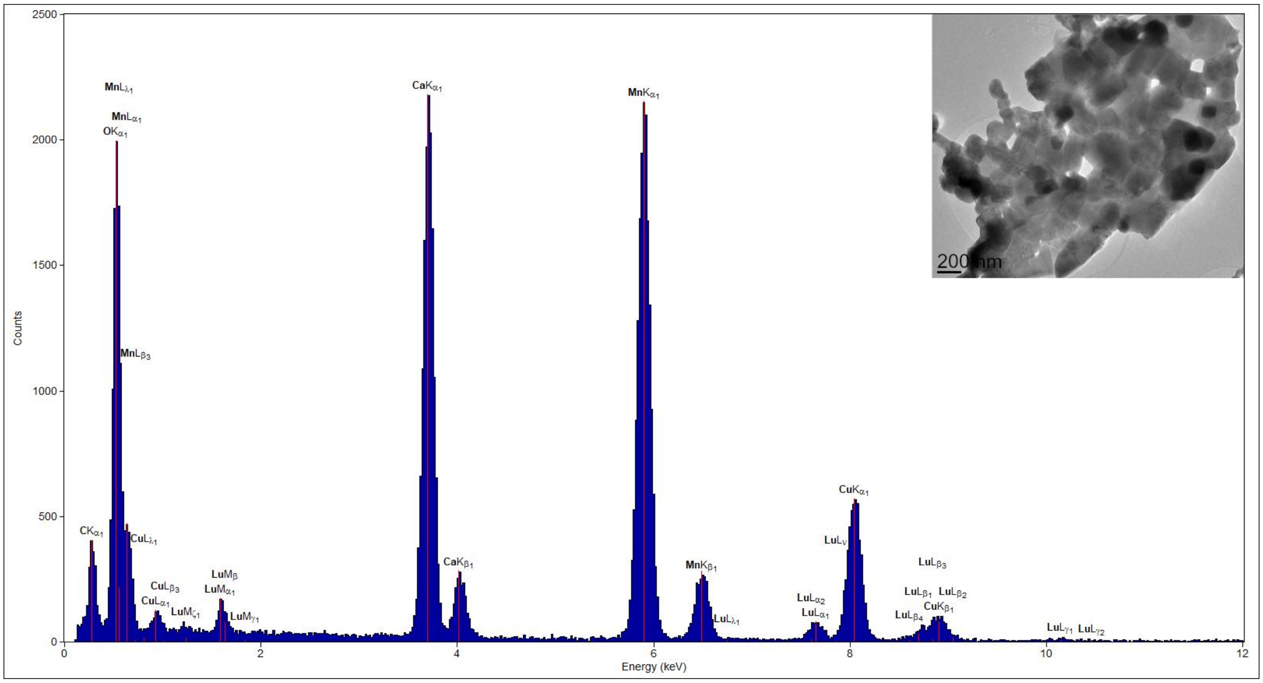

Figure S76. EDX analysis of $CaMnO_3$:Lu(III) crystallites. The copper and carbon elements come from the use of copper-carbon grids.

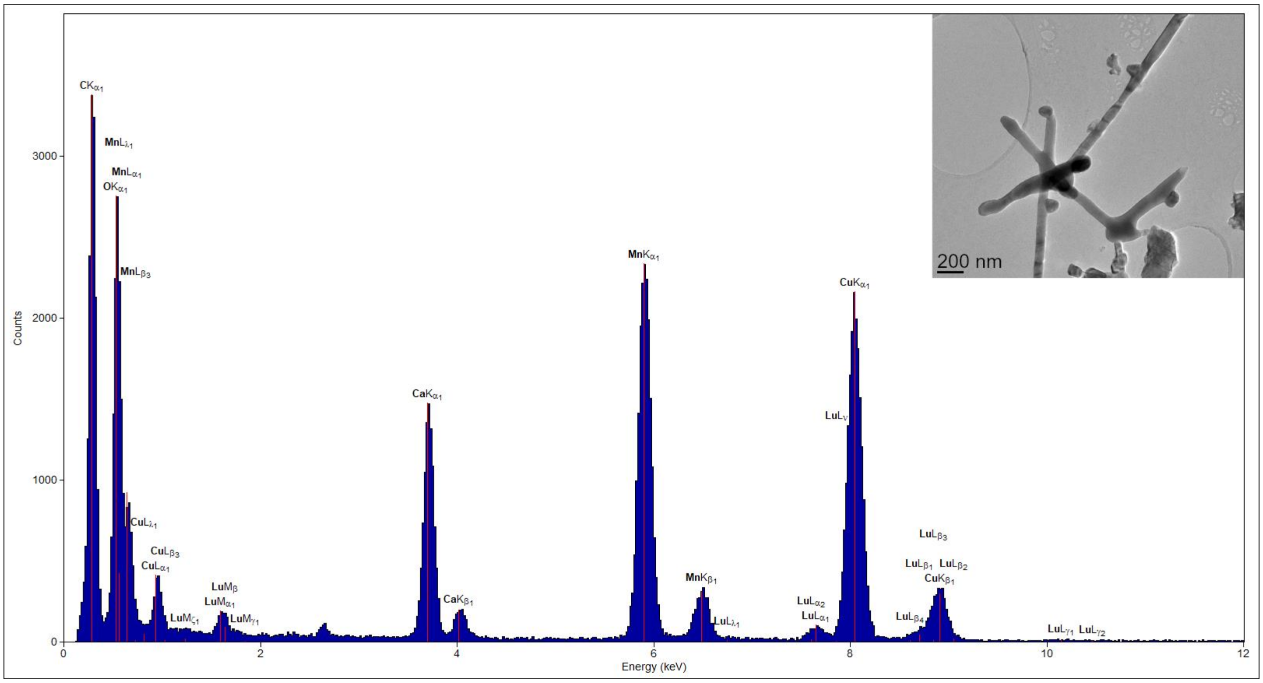

Figure S77. EDX analysis of $CaMn_2O_4$:Lu(III) crystallites. The copper and carbon elements come from the use of copper-carbon grids.

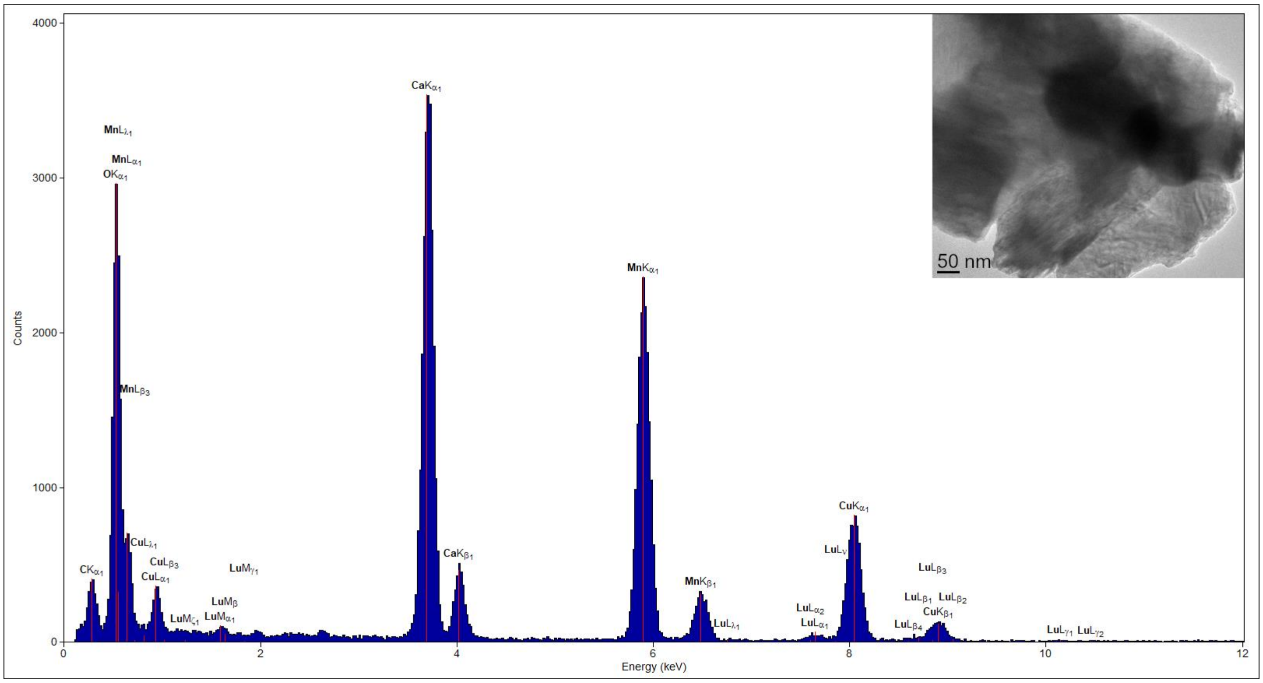

Figure S78. EDX analysis of Ca_2MnO_4:Lu(III) crystallites. The copper and carbon elements come from the use of copper-carbon grids.

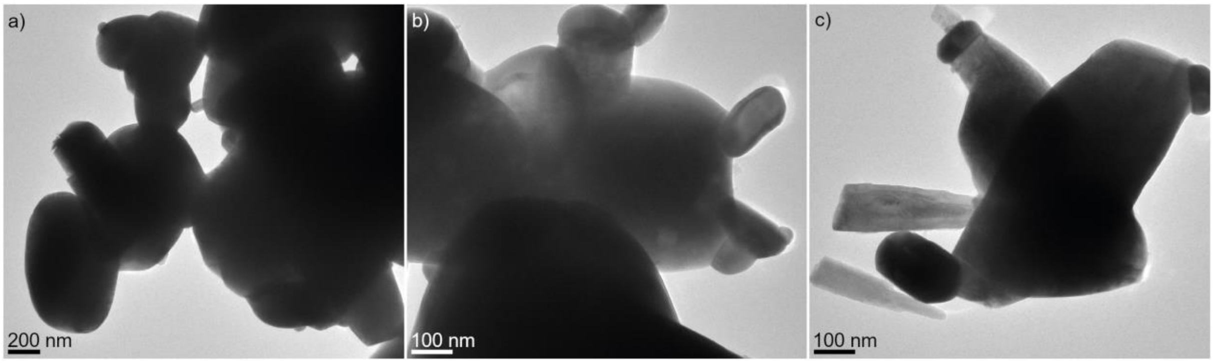

Figure S79. TEM images of oxide materials prepared by calcination of **8** with **9** in the presence of 0.22 mol % of **6** (a–c) at 1100 °C.

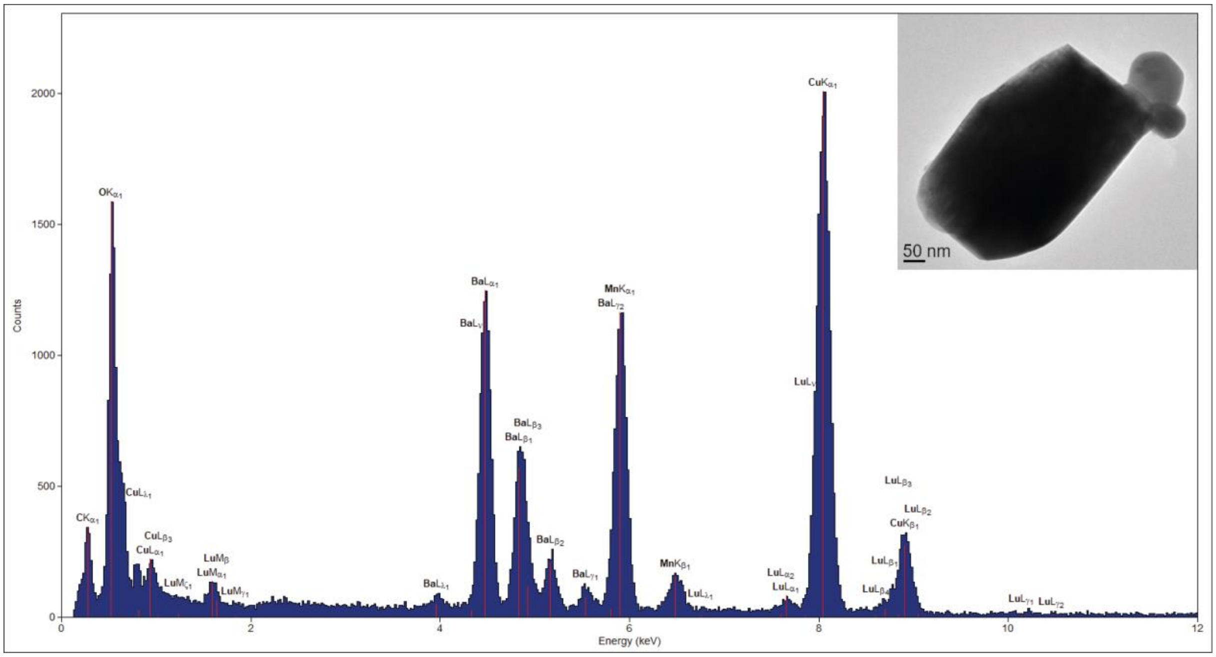

Figure S80. EDX analysis of $BaMnO_3$:Lu(III) crystallite. The copper and carbon elements come from the use of copper-carbon grids.

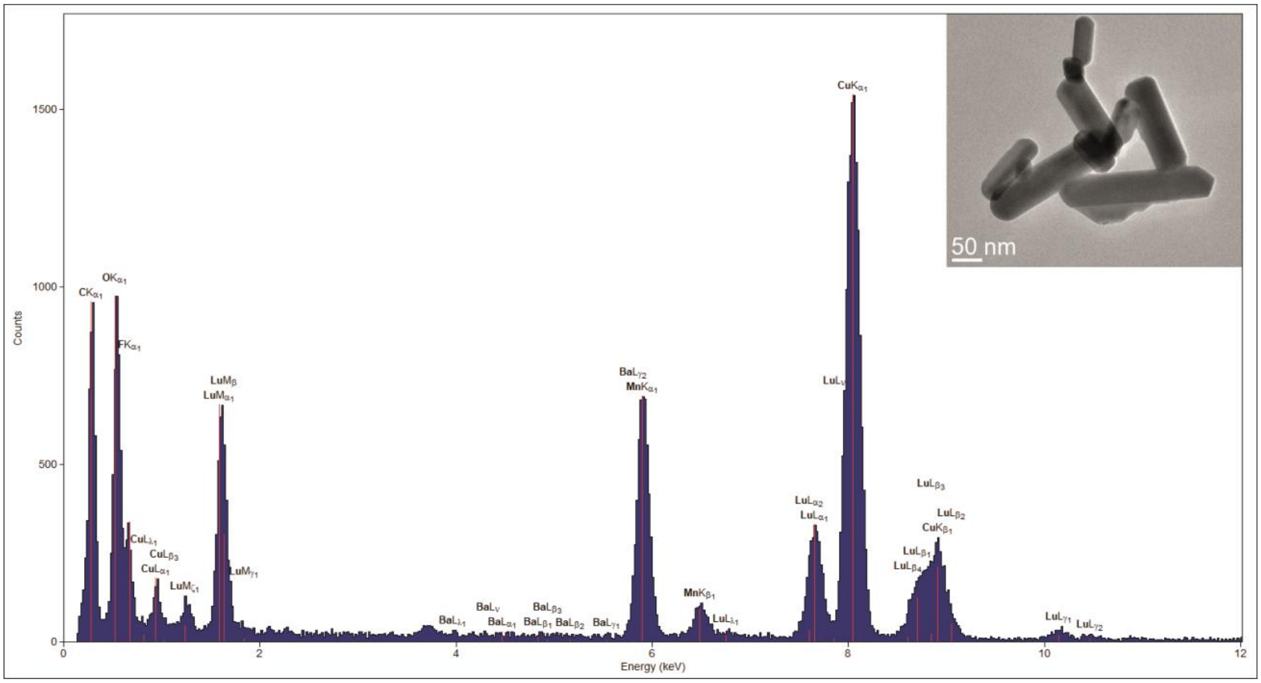

Figure S81. EDX analysis of $LuMnO_3$ rod-like crystallites found in oxide materials prepared by calcination of **8** with **9** in the presence of 0.22 mol % of **6** at 1100 °C. The copper and carbon elements come from the use of copper-carbon grids.
